# Total Synthesis of the Sex Pheromone of *Clania variegata* Snellen and Its Stereoisomers

**DOI:** 10.3390/ijms25094893

**Published:** 2024-04-30

**Authors:** Xueyang Wang, Jianwei Wu, Jianan Wang, Dan Liu, Qinghua Bian, Jiangchun Zhong

**Affiliations:** Department of Applied Chemistry, China Agricultural University, 2 West Yuanmingyuan Road, Beijing 100193, China; snowang@alu.cau.edu.cn (X.W.); w15103702532@163.com (J.W.); xxjnwang@163.com (J.W.); dliu@cau.edu.cn (D.L.); bianqinghua@cau.edu.cn (Q.B.)

**Keywords:** paulownia bagworm, chiral pool, sex pheromone, total synthesis

## Abstract

The paulownia bagworm, *Clania variegata* Snell, is an economically important pest of agriculture and forests. The sex pheromone of this pest and its stereoisomers were synthesized, and two of the stereoisomers were prepared for the first time. Our strategy was efficient and mainly included the ring-opening reaction of (*S*)-2-methyloxirane, the coupling of chiral sulfonate, the oxidative cleavage of olefin, and Yamaguchi esterification. Moreover, the overall yields of our synthesis were 23–29%, with eight steps in the longest route.

## 1. Introduction

The paulownia bagworm, *Clania variegata* Snell (synonyms for *Eumeta japonica* and *Eumeta variegata*), has become an economically important pest of agriculture and forests [1,2]. This pest is distributed in Australia, Korea [3], China, Indonesia [4], and Japan [5]. The larvae mainly defoliate various trees including apple, pear, peach, walnut, empress tree, gingko, and mulberry, and they also damage many agricultural crops, such as tea, citrus, coffee, loquat, maize, cotton, and soybean [6,7]. Due to it living within a self-enclosing bag coated with silk throughout all life stages [8,9], it is difficult to control the paulownia bagworm.

**Figure 1:**
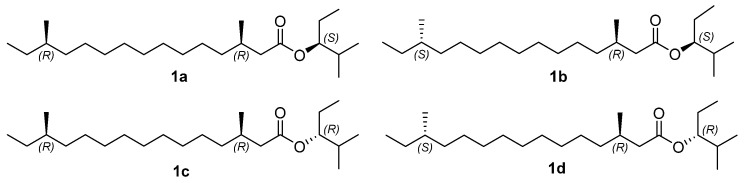
The sex pheromone of *Clania vartegata* Snell and its stereoisomers.

Sex pheromones have become an important component for the integrated pest management of agricultural pests, which has the advantages of being environmentally benign, highly efficient, and showing a lack of toxicity to mammals [10,11]. In 2006, Gries identified (*S*)-2-methylpentan-3-yl 3,13-dimethylpentadecanoate as a major component of the sex pheromone of *Clania variegata* Snellen by gas chromatographic electroantennographic detection (GC-EAD) analyses and field trapping experiments [7]. Later, Mori prepared four stereoisomers of (*S*)-2-methylpentan-3-yl 3,13-dimethylpentadecanoate via olefin cross metathesis, and their bioassay revealed the (3*R*,13*R*,3′*S*)-isomer (**1a**) as the bioactive one [12,13]. Other total syntheses of the sex pheromone are based on the S_N_2 reactions of secondary sulfonates and Evans template inductions [14,15]. To search for a more efficient synthesis method for the sex pheromone of *Clania vartegata* Snel, and provide stereoisomers for further biological research, herein, a new and concise synthesis of the sex pheromone of *Clania variegata* Snellen and its stereoisomers using Chiral Pool (Figure 1) has been developed.

## 2. Results and Discussion

### 2.1. Retrosynthetic Analysis of Sex Pheromone of Clania vartegata Snell ***1a***

**Figure 2:**
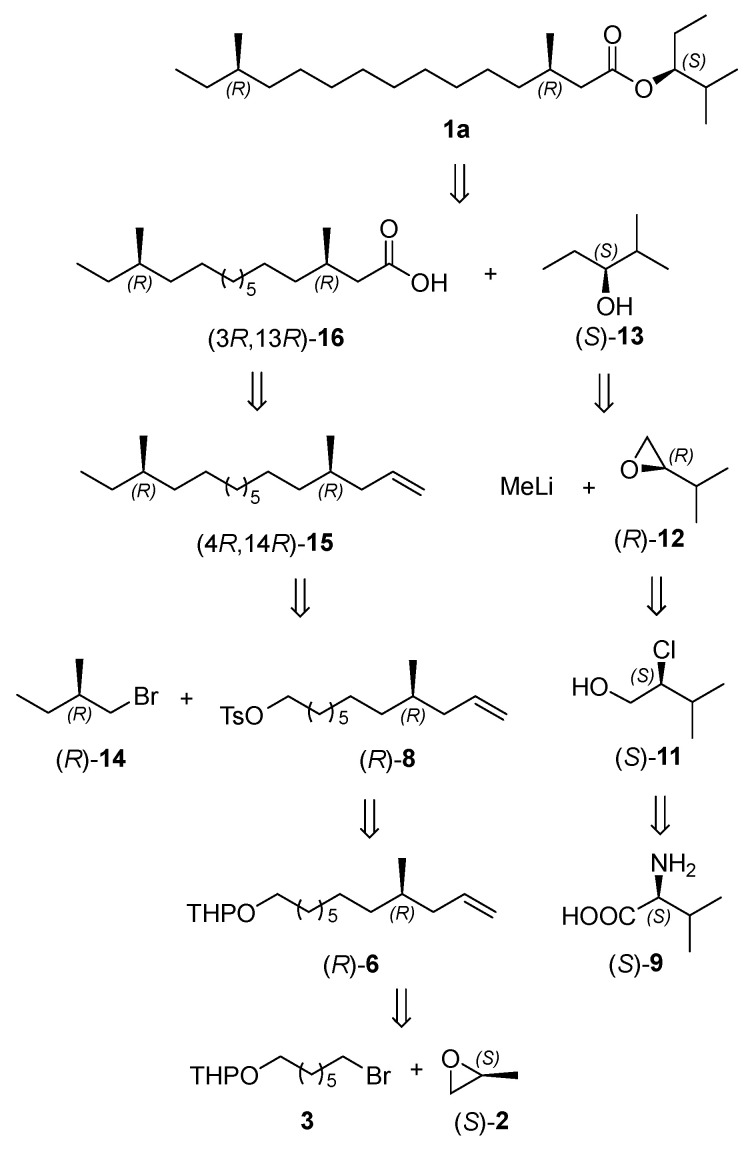
Retrosynthetic analysis of sex pheromone of *Clania vartegata* Snell **1a**.

Figure 2 shows a retrosynthetic analysis of the sex pheromone of *Clania vartegata* Snell **1a**. Obviously, the target sex pheromone **1a** could be prepared through the Yamaguchi esterification of (3*R*,13*R*)-3,13-dimethylpentadecanoic acid ((3*R*,13*R*)-**16**) with (*S*)-2-methylpentan-3-ol ((*S*)-**13**). Chiral dimethyl acid (3*R*,13*R*)-**16** was envisaged to be obtained from the oxidative cleavage of olefin (4*R*,14*R*)-**15**, which could be easily synthesized by the coupling of chiral sulfonate (*R*)-**8** with Grignard reagent derived from chiral bromide (*R*)-**14** and Mg. The chiral methyl of (*R*)-**8** would come from the ring-opening reaction of chiral-source (*S*)-2-methyloxirane ((*S*)-**2**). The key chiral subunit (*S*)-**13** could be generated from the addition of MeLi to (*R*)-2-isopropyloxirane ((*R*)-**12**). It was envisaged that the cyclization of chlorohydrin (*S*)-**11**, and diazotization and chlorination from (*S*)-valine ((*S*)-**9**) could provide epoxide (*R*)-**12**. Following a similar procedure as for **1a**, its stereoisomers **1c**–**1d** could be synthesized.

### 2.2. Synthesis of Chiral Tosylate (R)-***8***

**Scheme 1:**
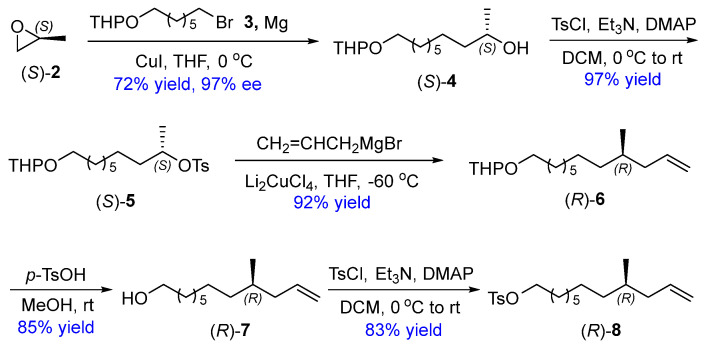
Synthesis of chiral sulfonate (*R*)-**8**.

Based on the retrosynthetic analysis, our synthesis commenced with the preparation of chiral tosylate (*R*)-**8** (Scheme 1). In the presence of CuI, the ring-opening reaction of (*S*)-2-methyloxirane ((*S*)-**2**) with Grignard reagent derived from 2-((7-bromoheptyl)oxy)tetrahydro-*2H*-pyran (**3**) and Mg afforded (*S*)-10-((tetrahydro-2*H*-pyran-2-yl)oxy)decan-2-ol ((*S*)-**4**) (72% yield, 97% ee, determined by ^1^H NMR spectrum of its Mosher ester) [16]. The subsequent tosylation of chiral alcohol (*S*)-**4** with *p*-TsCl gave (*S*)-10-((tetrahydro-2*H*-pyran-2-yl)oxy)decan-2-yl 4-methylbenzenesulfonate ((*S*)-**5**) in an excellent yield [17]. LiCuCl_4_ catalyzed coupling with allyl magnesium bromide and then converted the second tosylate (*S*)-**5** to 2-(((*R*)-9-methyldodec-11-en-1-yl)oxy)tetrahydro-*2H*-pyran ((*R*)-**6**) via S_N_2 nucleophilic substitution [18]. Finally, the deprotection of the primary hydroxy of (*R*)-**6** with *p*-TsOH and tosylation with *p*-TsCl provided the key chiral building block (*R*)-**8** [19].

### 2.3. Synthesis of Chiral Alcohols (R)-***13*** and (S)-***13***

**Scheme 2:**
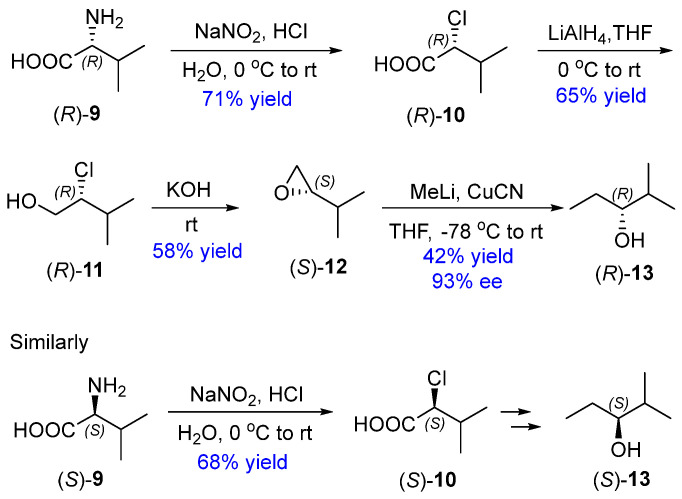
Synthesis of chiral alcohols (*R*)-**13** and (*S*)-**13**.

Having achieved the preparation of chiral sulfonate (*R*)-**8**, we next investigated the synthesis of another two key chiral subunits (*R*)-**13** and (*S*)-**13** (Scheme 2). Treating (*R*)-valine ((*R*)-**9**) with hydrochloric acid and NaNO_2_ resulted in the formation of (*R*)-2-chloro-3-methylbutanoic acid ((*R*)-**10**) [20], which was converted to (*R*)-2-chloro-3-methylbutan-1-ol ((*R*)-**11**) via reduction with LiAlH_4_ [21]. Chlorohydrin (*R*)-**11** was then converted into (*S*)-2-isopropyloxirane ((*S*)-**12**) by base-promoted cyclization [22]. The final CuCN- catalyzed the addition of MeLi to epoxide (*S*)-**12**, which afforded (*R*)-2-methylpentan-3-ol ((*R*)-**13**) with 93% ee, as determined by chiral HPLC of its 3,5-dinitrobenzoate [23]. Following a similar procedure as for chiral alcohol (*R*)-**13**, (*S*)-2-methylpentan-3-ol ((*S*)-**13**) (>99% ee) was prepared from (*S*)-valine ((*S*)-**9**).

### 2.4. Synthesis of Chiral Acids (3R,13R)-***16*** and (3R,13S)-***16***

**Scheme 3:**
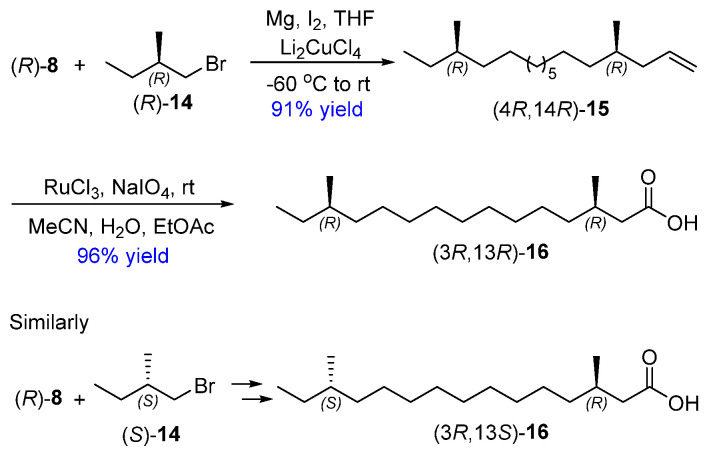
Synthesis of chiral acids (3*R*,13*R*)-**16** and (3*R*,13*S*)-**16**.

Scheme 3 shows the synthesis of the two key chiral acids (3*R*,13*R*)-**16** and (3*R*,13*S*)-**16**. (*R*)-1-Bromo-2-methylbutane ((*R*)-**14**) was reacted with Mg to yield Grignard reagent, which was coupled to chiral sulfonate (*R*)-**8** and afforded (4*R*,14*R*)-4,14-dimethylhexadec-1-ene ((4*R*,14*R*)-**15**) in 91% yield [24]. Then, RuCl_3_ catalyzed the oxidative cleavage of olefin (4*R*,14*R*)-**15** with NaIO_4_ to (3*R*,13*R*)-3,13-dimethylpentadecanoic acid ((3*R*,13*R*)-**16**), and this was conducted smoothly [25]. Similarly, the coupling of chiral sulfonate (*R*)-**8** with Grignard reagent, derived from (*S*)-1-bromo-2-methylbutane ((*S*)-**14**) and Mg, provided olefin (4*R*,14*S*)-**15**, followed by oxidation with NaIO_4_ which gave chiral acid (3*R*,13*S*)-**16**.

### 2.5. Synthesis of Sex Pheromone of Clania vartegata Snell ***1a*** and Its Stereoisomers ***1c***–***1d***

**Scheme 4:**
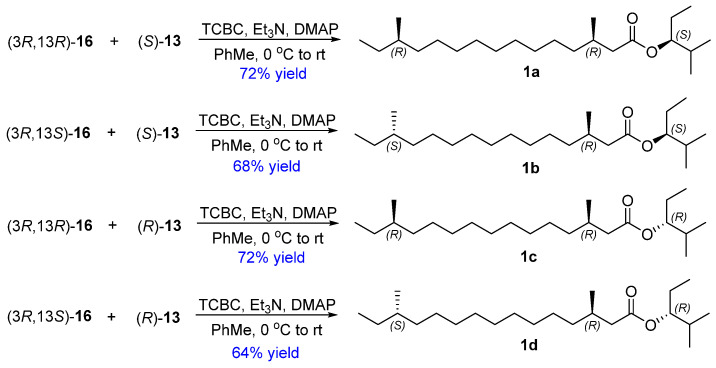
Synthesis of sex pheromone of *Clania vartegata* Snell **1a** and its stereoisomers **1b**–**1d**.

With four key chiral subunits in hand, we prepared the target sex pheromone **1a** and its stereoisomers **1c**–**1d** (Scheme 4). According to the Yamaguchi reaction (2,4,6-trichlorobenzoyl chloride, Et_3_N, DMAP) [26], the esterification of chiral dimethyl acid (3*R*,13*R*)-**16** with chiral alcohol (*S*)-**13** delivered (*S*)-2-methylpentan-3-yl (3*R*,13*R*)-3,13-dimethylpentadecanoate (**1a**), whereas the Yamaguchi esterification of chiral dimethyl acid (3*R*,13*S*)-**16** with chiral alcohol (*S*)-**13** provided (*S*)-2-methylpentan-3-yl (3*R*,13*S*)-3,13-dimethylpentadecanoate (**1b**). The specific rotation, NMR spectra, and HRMS of the target sex pheromone **1a** and its stereoisomer **1b** were consistent with the literature values [13,14]. Moreover, another two stereoisomers **1c** and **1d** were prepared, respectively, through Yamaguchi esterification of the chiral dimethyl acids (3*R*,13*R*)-**16** and (3*R*,13*S*)-**16** with chiral alcohol (*R*)-**13**, which were characterized with specific rotation, NMR spectra, and HRMS. The overall yields of **1a**–**1d** were 29%, 25%, 29%, and 23%, respectively, which were higher than those in the literature [12,13,14,15].

## 3. Materials and Methods

### 3.1. Synthesis of Chiral Acid (3R,13R)-***16***

#### 3.1.1. Synthesis of (4*R*,14*R*)-4,14-dimethylhexadec-1-ene ((4*R*,14*R*)-**15**)

Under an argon atmosphere, Mg (0.26 g, 10.70 mmol) was added to a 50 mL three-neck flask equipped with a condenser at room temperature. Dry THF (7 mL) and a few pellets of I_2_ were then added, followed by the addition of (*R*)-1-bromo-2-methylbutane ((*R*)-**14**) (0.20 g, 1.32 mmol). The resulting mixture was heated cautiously to initiate the reaction, and additional chiral bromide (*R*)-**14** (0.86 g, 5.70 mmol) was then added dropwise via a syringe. The reaction mixture was refluxed for 1 h while the continuous bubbles were formed. After being cooled to room temperature, a solution of (*R*)-(2-methylbutyl)magnesium bromide in THF (7 mL) was prepared.

Under an argon atmosphere, previously prepared (*R*)-(2-methylbutyl)magnesium bromide in THF (7 mL) was added to a separate 100 mL Schlenk flask at room temperature. After being cooled to −60 °C, Li_2_CuCl_4_ (0.7 mL, 0.1 M in THF, 0.07 mmol) and chiral tosylate (*R*)-**8** (0.49 g, 1.39 mmol) in THF (4 mL) were then added slowly. The reaction mixture was allowed to warm to room temperature and stirred for 8 h, and it was then quenched with saturated NH_4_Cl solution (5 mL). The aqueous phase was separated and extracted with EtOAc (3 × 10 mL). The extracts were combined with the organic phase and washed with saturated brine (15 mL), and then dried over anhydrous Na_2_SO_4_. The solvent was evaporated under reduced pressure and the residue was purified by column chromatography on silica gel (petroleum ether) to afford (4*R*,14*R*)-4,14-dimethylhexadec-1-ene ((4*R*,14*R*)-**15**) (0.32 g, 91% yield) as a colorless oil. [α]_D_^22^ = −3.98 (c = 1.41, CHCl_3_). ^1^H NMR (500 MHz, CDCl_3_) δ 5.77 (ddt, *J* = 17.2, 10.1, 7.1 Hz, 1H), 5.01–4.95 (m, 2H), 2.08–2.03 (m, 1H), 1.90–1.85 (m, 1H), 1.49–1.47 (m, 1H), 1.33–1.24 (m, 17H), 1.15–1.07 (m, 4H), 0.87 (d, *J* = 3.0 Hz, 3H), 0.85 (d, *J* = 3.0 Hz, 3H), 0.84 (t, *J* = 3.0 Hz, 3H). ^13^C NMR (126 MHz, CDCl_3_) δ 137.98, 115.50, 41.62, 36.82, 36.75, 34.57, 32.95, 30.21, 30.11, 29.90, 29.88, 29.87, 29.67, 27.29, 27.26, 19.61, 19.38, 11.57. HRMS (ESI) *m*/*z*: calcd for C_18_H_37_ [M+H]^+^ 253.2890, found 253.2882.

#### 3.1.2. Synthesis of (3*R*,13*R*)-3,13-dimethylpentadecanoic Acid ((3*R*,13*R*)-**16**)

NaIO_4_ (0.88 g, 4.11 mmol), H_2_O (3 mL), EtOAc (2 mL), and MeCN (2 mL) were added sequentially to a 50 mL three-neck flask and stirred for 10 min at room temperature. Chiral olefin (3*R*,13*R*)-**15** (0.25 g, 1.00 mmol) and RuCl_3_ (4.0 mg, 0.02 mmol) were then added. The reaction mixture was maintained for 1 h at room temperature and was diluted with H_2_O (10 mL). The aqueous phase was separated and extracted with EtOAc (3 × 10 mL). The extracts were combined with the organic phase and washed with saturated brine (30 mL), and then dried over anhydrous Na_2_SO_4_. The solvent was evaporated under reduced pressure to afford (3*R*,13*R*)-3,13-dimethylpentadecanoic acid ((3*R*,13*R*)-**16**) (0.26 g, 96% yield) as a colorless oil. [α]_D_^25^ = −7.18 (c = 1.73, CHCl_3_). Lit. [14] [α]_D_^25^ = −4.91 (c = 0.63, CHCl_3_). ^1^H NMR (500 MHz, CDCl_3_) δ 8.43 (br s, 1H), 2.31 (dd, *J* = 14.7, 5.7 Hz, 1H), 2.07 (dd, *J* = 14.6, 8.3 Hz, 1H), 1.93–1.89 (m, 1H), 1.33–1.26 (m, 18H), 1.15–1.06 (m, 3H), 0.93 (d, *J* = 6.7 Hz, 3H), 0.86 (d, *J* = 7.0 Hz, 3H), 0.84 (t, *J* = 3.0 Hz, 3H). ^13^C NMR (126 MHz, CDCl_3_) δ 179.78, 42.44, 36.99, 36.81, 34.56, 30.45, 30.21, 29.99, 29.91, 29.87, 29.65, 27.29, 27.13, 19.82, 19.37, 11.56. HRMS (ESI) *m*/*z*: calcd for C_17_H_35_O_2_ [M+H]^+^ 271.2632, found 271.2634.

### 3.2. Synthesis of Chiral Acid (3R,13S)-***16***

#### 3.2.1. Synthesis of (4*R*,14*S*)-4,14-dimethylhexadec-1-ene ((4*R*,14*S*)-**15**)

Following a similar procedure as for olefin (3*R*,13*R*)-**15**, the coupling of chiral tosylate (*R*)-**8** (0.49 g, 1.39 mmol) with Grignard reagent derived from (*S*)-1-bromo-2-methylbutane ((*S*)-**14**) (1.06 g, 7.01 mmol), catalyzed by Li_2_CuCl_4_ (0.7 mL, 0.1 M THF solution, 0.07 mmol), provided (4*R*,14*S*)-4,14-dimethylhexadec-1-ene ((4*R*,14*S*)-**15**) (0.29 g, 83% yield) as a colorless oil. [α]_D_^22^ = +8.02 (c =1.75, CHCl_3_). ^1^H NMR (500 MHz, CDCl_3_) δ 5.78 (ddt, *J* = 17.2, 10.1, 7.1 Hz, 1H), 5.00–4.96 (m, 2H), 2.08–2.03 (m, 1H), 1.90–1.84 (m, 1H), 1.49–1.46 (m, 1H), 1.31–1.24 (m, 17H), 1.16–1.06 (m, 4H), 0.87 (d, *J* = 3.0 Hz, 3H), 0.85 (d, *J* = 2.5 Hz, 3H), 0.84 (t, *J* = 3.0 Hz, 3H). ^13^C NMR (126 MHz, CDCl_3_) δ 136.99, 114.50, 40.61, 35.81, 35.74, 33.56, 31.94, 29.20, 29.10, 28.90, 28.87, 28.86, 28.66, 26.28, 26.25, 18.61, 18.38, 10.57. HRMS (ESI) *m*/*z*: calcd for C_18_H_37_ [M+H]^+^ 253.2890, found 253.2883.

#### 3.2.2. Synthesis of (3*R*,13*S*)-3,13-dimethylpentadecanoic Acid ((3*R*,13*S*)-**16**)

Following a similar procedure as for chiral dimethyl acid (3*R*,13*R*)-**16**, the oxidation of (4*R*,14*S*)-4,14-dimethylhexadec-1-ene ((4*R*,14*S*)-**15**) (252 mg, 1.00 mmol) with NaIO_4_ (0.88 g, 4.11 mmol) and RuCl_3_ (4.0 mg, 0.02 mmol) provided (3*R*,13*S*)-3,13-dimethylpentadecanoic acid ((3*R*,13*S*)-**16**) (0.26 g, 96% yield) as a colorless oil. [α]_D_^25^ = +8.63 (c = 1.07, CHCl_3_). Lit. [13] [α]_D_^24^ = +9.46 (c = 1.36, CHCl_3_). ^1^H NMR (500 MHz, CDCl_3_) δ 11.31 (br s, 1H), 2.35 (dd, *J* = 15.0, 5.5 Hz, 1H), 2.14 (dd, *J* = 15.0, 8.2 Hz, 1H), 1.98–1.92 (m, 1H), 1.27 (d, *J* = 14.3 Hz, 18H), 1.17–1.03 (m, 3H), 0.96 (d, *J* = 6.6 Hz, 3H), 0.86 (d, *J* = 7.0 Hz, 3H), 0.84 (t, *J* = 3.5 Hz, 3H). ^13^C NMR (126 MHz, CDCl_3_) δ 179.64, 41.70, 36.82, 36.79, 34.55, 30.30, 30.17, 29.86, 29.80, 29.78, 29.65, 27.26, 27.04, 19.83, 19.37, 11.56. HRMS (ESI) *m*/*z*: calcd for C_17_H_35_O_2_ [M+H]^+^ 271.2632, found 271.2634.

### 3.3. Synthesis of Sex Pheromone of Clania vartegata Snell ***1a*** and Its Stereoisomers ***1c***–***1d***

#### 3.3.1. Synthesis of (*S*)-2-methylpentan-3-yl (3*R*,13*R*)-3,13-dimethylpentadecanoate (**1a**)

Under an argon atmosphere, chiral dimethyl acid (3*R*,13*R*)-**16** (30.0 mg, 0.11 mmol), Et_3_N (33.0 mg, 0.33 mmol), and toluene (0.5 mL) were added sequentially to a 10 mL Schlenk tube at 0 °C. The resulting mixture was stirred at the same temperature for 30 min, and 2,4,6-trichlorobenzoyl chloride (54.0 mg, 0.22 mmol) was then added via a syringe. The reaction mixture was allowed to warm to room temperature and stirred for 1 h. After being cooled t to 0 °C, DMAP (40.0 mg, 0.33 mmol) in toluene (0.5 mL) and chiral alcohol (*S*)-**13** (7.0 mg, 0.07 mmol) were added slowly via a syringe. The reaction solution was allowed to warm to room temperature and maintained for 2.5 h. Saturated NH_4_Cl solution (2 mL) was added to quench the reaction. The aqueous phase was separated and extracted with Et_2_O (3 × 210 mL). The extracts were combined with the organic phase and washed with saturated brine (30 mL), and then dried over anhydrous Na_2_SO_4_. The solvent was evaporated under reduced pressure and the residue was purified by column chromatography on silica gel (petroleum ether/EtOAc 20:1) to afford (*S*)-2-methylpentan-3-yl (3*R*,13*R*)-3,13-dimethylpentadecanoate (**1a**) (18.0 mg, 72% yield) as a colorless oil. [α]_D_^25^ = −1.59 (c = 1.01, CHCl_3_). Lit. [13] [α]_D_^25^ = −4.61 (c = 2.52, CHCl_3_). ^1^H NMR (500 MHz, CDCl_3_) δ 4.68 (ddd, *J* = 8.0, 5.5, 4.5 Hz, 1H), 2.31 (dd, *J* = 14.5, 6.0 Hz, 1H), 2.11 (dd, *J* = 14.5, 8.1 Hz, 1H), 1.99–1.94 (m, 1H), 1.85–1.80 (m, 1H), 1.59–1.52 (m, 3H), 1.35–1.26 (m, 18H), 1.16–1.12 (m, 1H), 1.10–1.07 (m, 1H), 0.94 (d, *J* = 6.6 Hz, 3H), 0.89 (d, *J* = 6.7 Hz, 6H), 0.88–0.84 (m, 6H), 0.83 (d, *J* = 3.5 Hz, 3H). ^13^C NMR (126 MHz, CDCl_3_) δ 173.51, 79.59, 42.42, 36.90, 36.80, 34.56, 31.01, 30.55, 30.18, 29.95, 29.86, 29.80, 29.65, 27.26, 27.08, 24.14, 19.90, 19.38, 18.76, 17.79, 11.56, 10.04. The spectroscopic data of ^1^H and ^13^C NMR are consistent with the literature values [13]. HRMS (ESI) *m*/*z*: calcd for C_23_H_46_O_2_K [M+K]^+^ 393.3129, found 393.3142.

#### 3.3.2. Synthesis of (*S*)-2-methylpentan-3-yl (3*R*,13*S*)-3,13-dimethylpentadecanoate (**1b**)

Following a similar procedure as for the sex pheromone **1a**, the Yamaguchi esterification of chiral dimethyl acid (3*R*,13*S*)-**16** (30.0 mg, 0.11 mmol) with chiral alcohol (*S*)-**13** (7.0 mg, 0.07 mmol) provided (*S*)-2-methylpentan-3-yl (3*R*,13*S*)-3,13-dimethylpentadecanoate (**1b**) (17.0 mg, 68% yield) as a pale yellow oil. [α]_D_^25^ = +3.08 (c = 0.78, CHCl_3_). Lit. [14] [α]_D_^25^ = +2.06 (c = 1.96, CHCl_3_). ^1^H NMR (500 MHz, CDCl_3_) δ 4.68 (dt, *J* = 8.0, 5.0, 5.0 Hz, 1H), 2.32–2.29 (m, 1H), 2.13–2.07 (m, 1H), 1.96–1.95 (m, 1H), 1.85–1.80 (m, 1H), 1.58–1.55 (m, 3H), 1.30–1.26 (m, 18H), 1.13–1.10 (m, 1H), 1.09–1.06 (m, 1H), 0.94 (d, *J* = 6.7 Hz, 3H), 0.89 (d, *J* = 6.6 Hz, 6H), 0.87–0.84 (m, 6H), 0.84 (d, *J* = 3.4 Hz, 3H). ^13^C NMR (126 MHz, CDCl_3_) δ 173.51, 79.58, 42.42, 36.92, 36.80, 34.55, 31.02, 30.59, 30.55, 30.18, 29.94, 29.86, 29.80, 29.65, 27.26, 27.07, 24.13,19.90, 19.38, 18.76, 17.79, 11.56, 10.04. The spectroscopic data of ^1^H and ^13^C NMR are consistent with the literature values [14]. HRMS (ESI) *m*/*z*: calcd for C_23_H_46_O_2_K [M+K]^+^ 393.3129, found 393.3139.

#### 3.3.3. Synthesis of (*R*)-2-methylpentan-3-yl (3*R*,13*R*)-3,13-dimethylpentadecanoate (**1c**)

Following a similar procedure as for the sex pheromone **1a**, the Yamaguchi esterification of chiral dimethyl acid (3*R*,13*R*)-**16** (30.0 mg, 0.11 mmol) with chiral alcohol (*R*)-**13** (7.0 mg, 0.07 mmol) provided (*R*)-2-methylpentan-3-yl (3*R*,13*R*)-3,13-dimethylpentadecanoate (**1c**) (18.0 mg, 72% yield) as a colorless oil. [α]_D_^25^ = +0.85 (c =0.47, CHCl_3_). Lit. [13] The enantiomer of **1c**, [α]_D_^23^ = −3.42 (c =1.20, CHCl_3_). ^1^H NMR (500 MHz, CDCl_3_) δ 4.70–4.66 (m, 1H), 2.33–2.28 (m, 1H), 2.13–2.09 (m, 1H), 1.97–1.93 (m, 1H), 1.85–1.80 (m, 1H), 1.57–1.54 (m, 3H), 1.34–1.25 (m, 18H), 1.13–1.11 (m, 1H), 1.09–1.06 (m, 1H), 0.94 (d, *J* = 6.5 Hz, 3H), 0.89 (d, *J* = 5.6 Hz, 6H), 0.87–0.84 (m, 6H), 0.84 (d, *J* = 3.4 Hz, 3H). ^13^C NMR (126 MHz, CDCl_3_) δ 173.51, 79.59, 42.42, 36.90, 36.79, 34.55, 31.00, 30.54, 30.17, 29.95, 29.86, 29.80, 29.65, 27.26, 27.08, 24.13, 19.92, 19.38, 18.75, 17.79, 11.56, 10.03. HRMS (ESI) *m*/*z*: calcd for C_23_H_46_O_2_K [M+K]^+^ 393.3129, found 393.3146.

#### 3.3.4. Synthesis of (*R*)-2-methylpentan-3-yl (3*R*,13*S*)-3,13-dimethylpentadecanoate (**1d**)

Following a similar procedure as for the sex pheromone **1a**, the Yamaguchi esterification of chiral dimethyl acid (3*R*,13*S*)-**16** (30.0 mg, 0.11 mmol) with chiral alcohol (*R*)-**13** (7.0 mg, 0.07 mmol) provided (*R*)-2-methylpentan-3-yl (3*R*,13*S*)-3,13-dimethylpentadecanoate (**1d**) (16.0 mg, 64% yield) as a colorless oil. [α]_D_^25^ = +10.68 (c = 0.49, CHCl_3_). Lit. [13] The enantiomer of **1d**, [α]_D_^23^ = −10.8 (c =1.31, CHCl_3_). ^1^H NMR (500 MHz, CDCl_3_) δ 4.68 (dt, *J* = 8.0, 5.0 Hz, 1H), 2.32–2.28 (m, 1H), 2.13–2.09 (m, 1H), 1.96–1.95 (s, 1H), 1.86–1.80 (m, 1H), 1.58–1.54 (m, 3H), 1.29–1.25 (m, 18H), 1.13–1.10 (m, 1H), 1.09–1.06 (m, 1H), 0.94 (d, *J* = 6.7 Hz, 3H), 0.89 (d, *J* = 6.6 Hz, 6H), 0.87–0.84 (m, 6H), 0.83 (d, *J* = 3.4 Hz, 3H). ^13^C NMR (126 MHz, CDCl_3_) δ 173.51, 79.58, 42.42, 36.89, 36.80, 34.55, 31.02, 30.55, 30.18, 29.94, 29.86, 29.80, 29.65, 27.26, 27.07, 24.13, 19.90, 19.38, 18.76, 17.79, 11.56, 10.04. HRMS (ESI) *m*/*z*: calcd for C_23_H_46_O_2_K [M+K]^+^ 393.3129, found 393.3147.

## 4. Conclusions

In summary, we have achieved a novel and efficient total synthesis method for the sex pheromone of *Clania variegata* Snellen and its stereoisomers. Furthermore, (*R*)-2-methylpentan-3-yl (3*R*,13*R*)-3,13-dimethylpentadecanoate (**1c**) and (*R*)-2-methylpentan-3-yl (3*R*,13*S*)-3,13-dimethylpentadecanoate (**1d**) were prepared for the first time. The core synthetic strategy involved the ring-opening reaction of (*S*)-2-methyloxirane, the coupling of chiral sulfonate, the oxidative cleavage of olefin, and Yamaguchi esterification. Compared with previous approaches, our synthesis method has the advantages of being more concise (eight steps in the longest route) and resulting in a higher overall yield (23–29%). Moreover, this synthetic sex pheromone and its stereoisomers would be valuable for control of the paulownia bagworm.

## Data Availability

The data presented in this article are available in the Supplementary Materials.

## Supplementary Materials

The following supporting information can be downloaded at: https://www.mdpi.com/article/10.3390/ijms25094893/s1. The syntheses of compounds **4**–**13**, the research on the enantiomeric purity of chiral alcohols **4** and **13**, the copies of ^1^H, ^13^C and ^19^F NMR spectra, and HPLC chromatograms.
