# Supplementary material for: Total Synthesis of the Sex Pheromone of Clania variegata Snellen and Its Stereoisomers
Source: Int J Mol Sci. 2024 Apr 30;25(9):4893. doi: 10.3390/ijms25094893 (PMC11084484; doi:10.3390/ijms25094893)
Supplement: Supplementary file 1 [file ijms-25-04893-s001.zip › ijms-2936475-supplementary.pdf]

SUPPORTING INFORMATION

Total Synthesis of Sex Pheromone of *Clania variegata* Snellen and Its Stereoisomers

Xueyang Wang[+], Jianwei Wu[+], Jianan Wang, Dan Liu, Qinghua Bian, and Jiangchun Zhong*

Department of Applied Chemistry, China Agricultural University, 2 West Yuanmingyuan Road, Beijing 100193, P. R. China;
* Correspondence: zhong@cau.edu.cn; Tel.: +8601062731356
[+] These authors contributed equally to this work.

Table of Contents

1. General Information……………………………………………………………….……S2

2. Synthesis of sulfonate (*R*)-**8**……………..……………..……………..……………..………..S2

3. Synthesis of chiral alcohol (*R*)-**13** and (*S*)-**13**………….…………..……………..………..S6

4. Research on the Enantiomeric Purity of Chiral Alcohols…………………………….....S10

5. ^{1}H, ^{13}C NMR Spectra of the Products…………………………………………………..S15

6. HPLC Chromatography of the Compounds…………………………………….……...S42

7. References……………………………………………………………………………….S43

1. General Information

Unless otherwise noted, all reactions were carried out in a Schlenk system under an argon atmosphere. Dichloromethane, acetonitrile, triethyl amine and tetrahydrofuran were purified by distillation from calcium hydride before use. All commercial reagents and starting materials were reagent grade and used without further purification. Optical rotations were measured on a Rudolph AUTOPOL-IV polarimeter. Enantiomeric excesses were determined by an Agilent 1200 HPLC equipped with a Daicel Chiralcel OJ-H column, and *n*-hexane and isopropanol as eluents. ^{1}H and ^{13}C NMR spectra were recorded in $CDCl_3$ on a Bruker Ascend™ 500 MHz spectrometer. Chemical shift values (δ) were reported in ppm and referenced to tetramethylsilane (0.00 ppm) for ^{1}H NMR and residual chloroform (77.16 ppm) for ^{13}C NMR. Exact masses were obtained by high resolution mass spectroscopy (HRMS) using a Waters LCT Premier™ spectrometer.

2.Synthesis of sulfonate (*R*)-8

Scheme S1. Synthesis of chiral sulfonate (*R*)-**8**.

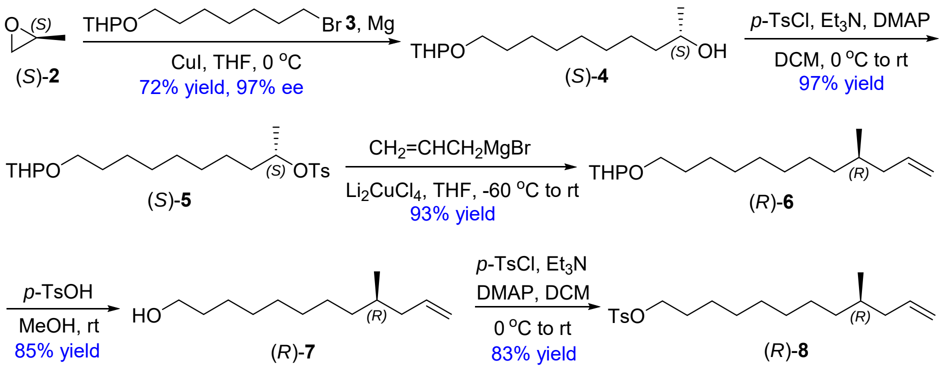

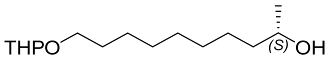

(*S*)-10-((tetrahydro-2*H*-pyran-2-yl)oxy)decan-2-ol ((*S*)-**4**) (CAS 161971-26-2) [1]

Under an argon atmosphere, Mg (0.52 g, 21.40 mmol) was added to a 50 mL three-neck flask

equipped with a condenser at room temperature. Dry THF (20 mL) and a few pellets of I_2 were then added, followed by the addition of 2-((7-bromoheptyl)oxy)tetrahydro-2*H*-pyran (**3**) (0.80 g, 2.87 mmol). The resulting mixture was heated cautiously to initiate the reaction, and additional bromide **3** (3.20 g, 11.46 mmol) was then added dropwise via a syringe. The reaction mixture was refluxed for 1 h while the continuous bubbles were formed. After being cooled to room temperature, a solution of (7-((tetrahydro-2*H*-pyran-2-yl)oxy)heptyl) magnesium bromide in THF (20 mL) was prepared.

Under an argon atmosphere, CuI (0.14 g, 0.74 mmol), THF (20 mL) and (*S*)-2-methyloxirane ((*S*)-**2**) (1.66 g, 28.58 mmol) were added to a separate 100 mL Schlenk flask at room temperature. The resulting mixture was cooled to 0 °C, and (7-((tetrahydro-2*H*-pyran-2-yl)oxy)heptyl) magnesium bromide in THF (20 mL) was then added slowly over 2 h. The reaction mixture was maintained at the same temperature for 10 h, and quenched with saturated NH_4Cl solution (20 mL) at 0 °C. After the resulting mixture was allowed to warm to room temperature, the aqueous phase was separated and extracted with EtOAc (3 ×20 mL). The extracts were combined with the organic phase and washed with saturated brine (20 mL), then dried over anhydrous Na_2SO_4. The solvent was evaporated under the reduce pressure and the residue was purified by column chromatography on silica gel (petroleum ether/EtOAc 5:1) to afford (*S*)-10-((tetrahydro-2*H*-pyran-2-yl)oxy)decan-2-ol ((*S*)-**4**) (2.66 g, 72% yield, 97% ee, determined by ^{1}H NMR spectrum of its Mosher ester) as a colorless oil. $[\alpha]_D^{22}$ = +4.91 (c = 5.70, $CHCl_3$). Lit.[2] $[\alpha]_D^{20}$ = +5.0 (c = 1.30, $CHCl_3$). ^{1}H NMR (500 MHz, $CDCl_3$) δ 4.57 (dd, *J* = 4.5, 2.9 Hz, 1H), 3.89 – 3.80 (m, 1H), 3.79 – 3.75 (m, 1H), 3.74 – 3.70 (m, 1H), 3.52 – 3.48 (m, 1H), 3.40 – 3.36 (m, 1H), 1.84 – 1.80 (m, 1H), 1.74 – 1.69 (m, 1H), 1.61 – 1.51 (m, 6H), 1.42 – 1.39 (m, 4H), 1.35 – 1.30 (m, 8H), 1.18 (d, *J* = 6.2 Hz, 3H). ^{13}C NMR (126 MHz, $CDCl_3$) δ 98.98, 68.28, 67.80, 62.48, 39.49, 30.92, 29.87, 29.71, 29.66, 29.54, 26.35, 25.87, 25.64, 23.62, 19.83. HRMS (ESI) *m*/*z*: calcd for $C_{15}H_{30}O_3Na$ $[M+Na]^+$ 281.2087, found 281.2090.

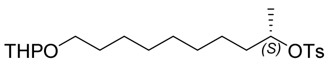

(*S*)-10-((tetrahydro-2*H*-pyran-2-yl)oxy)decan-2-yl 4-methylbenzenesulfonate ((*S*)-**5**) (new compound) [3]

Under an argon atmosphere, DMAP (1.46 g, 11.95 mmol), DCM (50 mL), Et_3N (1.45 g, 14.35 mmol) and *p*-TsCl (2.74 g, 14.37 mmol) were added to a 100 mL Schlenk flask at room temperature. The resulting solution was cooled to 0 °C, and chiral alcohol (*S*)-**4** (3.09 g, 11.96 mmol) in DCM (3 mL) was added slowly. The reaction solution was warmed to room temperature and stirred for 10 h, followed by the quenching with saturated NH_4Cl solution (20 mL). The aqueous phase was separated and extracted with EtOAc (3 × 30 mL). The extracts were combined with the organic phase and washed with saturated brine (50 mL), then dried over anhydrous Na_2SO_4. The solvent was evaporated under the reduce pressure and the residue was purified by column chromatography on silica gel (petroleum ether/Et_2OAc 10:1) to afford (*S*)-10-((tetrahydro-2*H*-pyran-2-yl)oxy)decan-2-yl 4-methylbenzenesulfonate ((*S*)-**5**) (4.78 g, 97% yield) as a pale yellow oil. $[\alpha]_D^{22}$ = –0.50 (c = 1.59, $CHCl_3$). ^1H NMR (500 MHz, $CDCl_3$) δ 7.79 (dd, *J* = 8.4, 1.5 Hz, 2H), 7.33 (dd, *J* = 8.0, 2.0 Hz, 2H), 4.62 – 4.56 (m, 2H), 3.89 – 3.85 (m, 1H), 3.75 – 3.70 (m, 1H), 3.52 – 3.48 (m, 1H), 3.40 – 3.35 (m, 1H), 2.44 (s, 3H), 1.85 – 1.82 (m, 1H), 1.74 – 1.69 (m, 1H), 1.59 – 1.51 (m, 6H), 1.32 – 1.16 (m, 15H). ^{13}C NMR (126 MHz, $CDCl_3$) δ 143.48, 133.80, 128.82, 126.85, 98.03, 79.80, 66.79, 61.53, 35.62, 29.94, 28.87, 28.45, 28.22, 25.33, 24.64, 23.99, 20.74, 19.99, 18.87. HRMS (ESI) m/z: calcd for $C_{22}H_{37}O_5S$ $[M+H]^+$ 413.2356, found 413.2354.

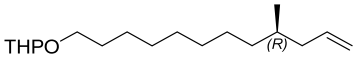

2-(((*R*)-9-methyldodec-11-en-1-yl)oxy)tetrahydro-2*H*-pyran ((*R*)-**6**) (new compound) [4]

Under an argon atmosphere, allyl magnesium bromide (7.20 mL, 1.0 M in THF, 7.20 mmol) and THF (7 mL) were added to a 50 mL Schlenk flask at room temperature. After being cooled to -60 °C, Li_2CuCl_4 (0.90 mL, 0.1 M in THF, 0.09 mmol) was added and stirred for 10 min. Chiral tosylate (*S*)-**5** (0.74 g, 1.79 mmol) in THF (6 mL) was then add over 1 h at the same temperature. The reaction mixture was allowed to warm to room temperature and stirred for 8 h, and quenched with saturated NH_4Cl solution (5 mL). The aqueous phase was separated and extracted with EtOAc (3 × 10 mL). The extracts were combined with the organic phase and washed with saturated brine (10 mL), then dried over anhydrous Na_2SO_4. The solvent was evaporated under the reduce pressure and the residue was purified by column chromatography on silica gel (petroleum ether/ethyl acetate 20:1) to afford 2-(((*R*)-9-

methyldodec-11-en-1-yl)oxy)tetrahydro-2*H*-pyran ((*R*)-**6**) (0.47 g, 93% yield) as a colorless oil. $[\alpha]_D^{22}$ = +2.70 (c =1.78, $CHCl_3$). ^1H NMR (500 MHz, $CDCl_3$) δ 5.78 (ddt, *J* = 17.3, 10.3, 7.1 Hz, 1H), 5.00 – 4.96 (m, 2H), 4.57 (dd, *J* = 4.5, 2.8 Hz, 1H), 3.87 (td, *J* = 7.9, 3.9 Hz, 1H), 3.73 (dt, *J* = 9.6, 7.0 Hz, 1H), 3.51 – 3.48 (m, 1H), 3.38 (dt, *J* = 9.6, 6.7 Hz, 1H), 2.07 – 2.03 (m, 1H), 1.90 – 1.81 (m, 2H), 1.74 – 1.69 (m, 1H), 1.58 – 1.50 (m, 6H), 1.31 – 1.24 (m, 12H), 1.11 – 1.07 (m, 1H), 0.86 (d, *J* = 6.6 Hz, 3H). ^{13}C NMR (126 MHz, $CDCl_3$) δ 136.97, 114.50, 98.00, 66.85, 61.50, 40.59, 35.70, 31.92, 29.95, 29.01, 28.91, 28.76, 28.64, 26.20, 25.40, 24.67, 18.86, 18.60. HRMS (ESI) m/z: calcd for $C_{18}H_{35}O_2$ $[M+H]^+$ 283.2632, found 283.2631.

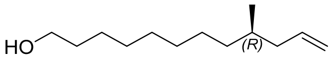

(*R*)-9-methyldodec-11-en-1-ol ((*R*)-**7**) (new compound) [5]

Chiral olefin (*R*)-**6** (0.94 g, 3.33 mmol) in MeOH (8 mL) was added to a 50 mL Schlenk tube at room temperature. *p*-TsOH (0.57 g, 3.33 mmol) was then added, and the reaction solution was maintained for 24 h at room temperature. The solvent was evaporated under the reduce pressure and the residue was purified by column chromatography on silica gel (petroleum ether/ethyl acetate 5:1) to afford (*R*)-9-methyldodec-11-en-1-ol ((*R*)-**7**) (0.56 g, 85% yield) as a colorless oil. $[\alpha]_D^{22}$ = +4.33 (c = 3.61, $CHCl_3$). ^1H NMR (500 MHz, $CDCl_3$) δ 5.78 (ddt, *J* = 17.2, 10.2, 7.1 Hz, 1H), 5.01 – 4.96 (m, 2H), 3.64 (t, *J* = 6.6 Hz, 2H), 2.09 – 2.03 (m, 1H), 1.91 – 1.85 (m, 1H), 1.58 – 1.54 (m, 2H), 1.50 – 1.44 (m, 1H), 1.36 – 1.24 (m, 12H), 1.12 – 1.07 (m, 1H), 0.86 (d, *J* = 6.6 Hz, 3H). ^{13}C NMR (126 MHz, $CDCl_3$) δ 136.96, 114.52, 62.26, 40.59, 35.69, 31.96, 31.91, 28.99, 28.77, 28.57, 26.19, 24.88, 18.60. HRMS (ESI) m/z: calcd for $C_{13}H_{26}OK$ $[M+K]^+$ 237.1615, found 237.1610.

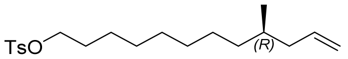

(*R*)-9-methyldodec-11-en-1-yl 4-methylbenzenesulfonate ((*R*)-**8**) (new compound)

Following the similar procedure for chiral tosylate (*S*)-**5**, the tosylation of chiral alcohol (*R*)-**7** (0.50 g, 2.50 mmol) with *p*-TsCl (0.57 g, 3.00 mmol) provided (*R*)-9-methyldodec-11-en-1-yl 4-methylbenzenesulfonate ((*R*)-**8**) (0.73 g, 83% yield) as a pale yellow oil. $[\alpha]_D^{22}$ = +2.17 (c = 2.39, $CHCl_3$). ^1H NMR (500 MHz, $CDCl_3$) δ 7.79 (d, *J* = 8.3 Hz, 2H), 7.34 (d, *J* = 8.0 Hz, 2H), 5.77 (ddt,

J = 17.3, 10.3, 7.2 Hz, 1H), 4.98 – 4.96 (m, 2H), 4.02 (t, J = 6.5 Hz, 2H), 2.45 (s, 3H), 2.07 – 2.02 (m, 1H), 1.90 – 1.84 (m, 1H), 1.66 – 1.59 (m, 2H), 1.48 – 1.44 (m, 1H), 1.30 – 1.21 (m, 11H), 1.10 – 1.07 (m, 1H), 0.85 (d, J = 6.7 Hz, 3H). ^{13}C NMR (126 MHz, $CDCl_3$) δ 143.74, 136.88, 132.37, 128.92, 127.01, 114.53, 69.83, 40.55, 35.62, 31.87, 28.86, 28.52, 28.05, 27.93, 26.12, 24.45, 20.76, 18.57. HRMS (ESI) m/z: calcd for $C_{20}H_{32}O_3SNa$ $[M+Na]^+$ 375.1964, found 375.1974.

3. Synthesis of chiral alcohol (*R*)-13 and (*S*)-13

Scheme S2. Synthesis of chiral alcohol (*R*)-**13**.

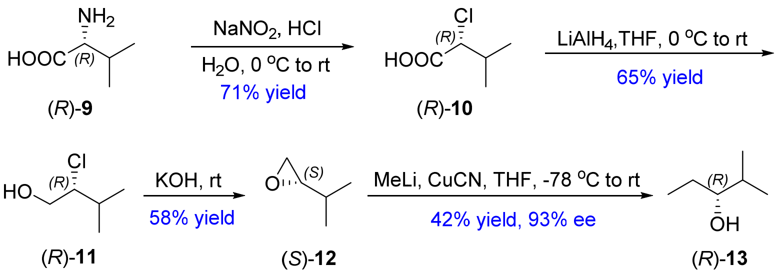

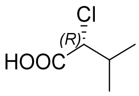

(*R*)-2-chloro-3-methylbutanoic acid ((*R*)-**10**) (CAS 84918-96-7) [6]

Hydrochloric acid (100 mL, 6 M in H_2O, 0.60 mol) and (*R*)-valine ((*R*)-**9**) (10.00 g, 85.36 mmol) were added to a 250 mL three-neck flask at room temperature. The resulting mixture was cooled to 0 °C, $NaNO_2$ (9.42 g, 136.65 mmol) in H_2O (30 mL) was then added dropwise via a syringe over 30 min. After the reaction mixture was allowed to warm to room temperature and stirred for 14 h, it was extracted with EtOAc (3 × 80 mL). The extracts were combined, and dried over anhydrous Na_2SO_4. The solvent was evaporated under the reduce pressure, and the residue was purified by column chromatography on silica gel (toluene/EtOAc = 4:1) to afford (*R*)-2-chloro-3-methylbutanoic acid ((*R*)-**10**) (8.30 g, 71% yield) as a pale yellow oil. $[\alpha]_D^{25}$ = +2.34 (c = 2.91, $CHCl_3$). Lit.[7] $[\alpha]_D^{20}$ = +2.6 (c = 10.0, CH_2Cl_2). ^1H NMR (500 MHz, $CDCl_3$) δ 11.06 (br s, 1H), 4.19 (d, J = 6.1 Hz, 1H), 2.38 – 2.34 (m, 1H), 1.08 (d, J = 7.0 Hz, 3H), 1.07 (d, J = 7.0 Hz, 3H).

^{13}C NMR (126 MHz, $CDCl_3$) δ 175.70, 64.06, 32.59, 19.71, 17.89. HRMS (ESI) m/z: calcd for $C_5H_{10}O_2Cl$ $[M+H]^+$ 137.0364, found 137.0361.

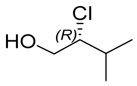

(*R*)-2-chloro-3-methylbutan-1-ol ((*R*)-**11**) (CAS 140388-27-8) [8]

Under an argon atmosphere, $LiAlH_4$ (1.25 g, 32.94 mmol) and THF (30 mL) were added to a 200 mL Schlenk flask at room temperature. After being cooled to 0 °C, chiral acid (*R*)-**10** (3.00 g, 21.97 mmol) in THF (15 mL) was added dropwise over 30 min. The reaction mixture was allowed to warm to room temperature and stirred for 8 h, and then neutralized with hydrochloric acid (2.5 M). The aqueous phase was separated and extracted with Et_2O (3 × 60 mL). The extracts were combined with the organic phase and washed with saturated $NaHCO_3$ solution (30 mL) and brine (30 mL) sequentially, then dried over anhydrous Na_2SO_4. The solvent was evaporated under the reduce pressure and the residue was purified by column chromatography on silica gel (*n*-pentane/Et_2O = 4:1) to afford (*R*)-2-chloro-3-methylbutan-1-ol ((*R*)-**11**) (1.76 g, 65% yield) as a pale yellow liquid. $[\alpha]_D^{25} = -1.63$ (c = 3.19, $CHCl_3$). ^1H NMR (500 MHz, $CDCl_3$) δ 3.94 – 3.91 (m, 1H), 3.84 – 3.79 (m, 1H), 3.76 – 3.71 (m, 1H), 2.19 – 2.13 (m, 1H), 2.10 – 2.03 (m, 1H), 1.04 (d, *J* = 6.9 Hz, 3H), 1.02 (d, *J* = 6.6 Hz, 3H). ^{13}C NMR (126 MHz, $CDCl_3$) δ 71.94, 65.54, 31.54, 20.09, 18.20. HRMS (ESI) m/z: calcd for $C_5H_{12}OCl$ $[M+H]^+$ 123.0571, found 123.0560.

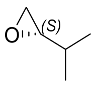

(*S*)-2-isopropyloxirane ((*S*)-**12**) (CAS 55123-01-8) [9]

At room temperature, powdered KOH (1.16 g, 20.67 mmol) was added to a 50 mL narrow-necked flask connected to a cooling trap, which was cooled to –78 °C. (*R*)-2-Chloro-3-methylbutan-1-ol ((*R*)-**11**) (1.15 g, 9.38 mmol) was then add under reduced pressure and stirred for 20 min at room temperature. The cooling trap was allowed to warm to room temperature and afforded (*S*)-2-isopropyloxirane ((*S*)-**12**) (0.47 g, 58% yield) as a colorless liquid. $[\alpha]_D^{25}$ = +1.98 (c = 0.81, $CHCl_3$). ^1H NMR (500 MHz, $CDCl_3$) δ 2.73 – 2.69 (m, 2H), 2.52 (dt, *J* = 3.9, 1.9 Hz,

1H), 1.51 – 1.46 (m, 1H), 1.04 (d, *J* = 6.7 Hz, 3H), 0.96 (d, *J* = 6.9 Hz, 3H). ^{13}C NMR (126 MHz, $CDCl_3$) δ 57.79, 46.26, 30.94, 19.18, 18.29. HRMS (ESI) m/z: calcd for $C_5H_{10}OK$ $[M+K]^+$ 125.0363, found 125.0355.

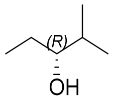

(*R*)-2-methylpentan-3-ol ((*R*)-**13**) (CAS 63814-74-4) [10]

Under an argon atmosphere, CuCN (0.37 g, 4.16 mmol) and THF (7 mL) were added to a 25 mL Schlenk tube. The resulting suspension was cooled to –78 °C, and MeLi (5.2 mL, 1.6 M in Et_2O, 8.32 mmol) was added slowly. The reaction mixture was allowed to warm to room temperature and stirred for 2 h. After being cooled –78 °C again, (*S*)-2-isopropyloxirane ((*S*)-**12**) (0.30 g, 3.48 mmol) was added dropwise via a syringe and stirred for 2 h. The reaction mixture was allowed to room temperature and maintained for additional 6 h. Saturated NH_4Cl solution (10 mL) was added at 0 °C to quench the reaction. The aqueous phase was separated and extracted with Et_2O (3 ×10 mL). The extracts were combined with the organic phase and washed with saturated brine (20 mL), then dried over anhydrous Na_2SO_4. The solvent was evaporated under reduce pressure and the residue was purified by column chromatography on silica gel (*n*-pentane/Et_2O = 5:1) (0.15 g, 42% yield, 93% ee, determined by chiral HPLC of its 3,5-dinitrobenzoate). ^1H NMR (500 MHz, $CDCl_3$) δ 3.30 – 3.27 (m, 1H), 1.69 – 1.63 (m, 1H), 1.57 – 1.49 (m, 1H), 1.44 – 1.37 (m, 1H), 1.33 (br s, 1H), 0.96 (t, *J* = 7.4 Hz, 3H), 0.92 (d, *J* = 4.5, 3H), 0.90 (d, *J* = 4.5, 3H). ^{13}C NMR (126 MHz, $CDCl_3$) δ 78.37, 33.20, 27.08, 19.07, 17.21, 10.43. HRMS (ESI) m/z: calcd for $C_6H_{14}OK$ $[M+K]^+$ 141.0676, found 141.0670.

Scheme S3. Synthesis of chiral alcohol (*S*)-**13**.

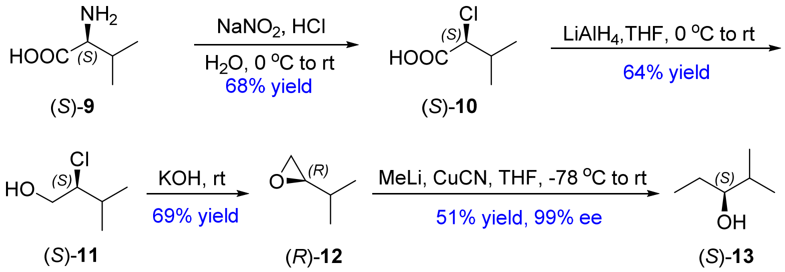

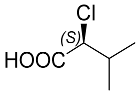

(S)-2-chloro-3-methylbutanoic acid ((S)-**10**) (CAS 26782-74-1)

Following the similar procedure for chiral acid (R)-**10**, the (S)-valine ((S)-**9**) (10.00 g, 85.36 mmol), $NaNO_2$ (9.42 g, 136.65 mmol) and hydrochloric acid (100 mL, 6 M in H_2O, 0.60 mol) provided (S)-2-chloro-3-methylbutanoic acid ((S)-**10**) (7.95 g, 68% yield) as a pale yellow oil. $[\alpha]_D^{25}$ = –2.06 (c = 3.69, $CHCl_3$), Lit.[11] $[\alpha]_D^{23}$ = –2.45 (c = 1.62, $CHCl_3$). ^1H NMR (500 MHz, $CDCl_3$) δ 11.14 (br s, 1H), 4.20 (d, J = 6.1 Hz, 1H), 2.40 – 2.33 (m, 1H), 1.09 (d, J = 6.5 Hz, 3H), 1.07 (d, J = 7.0 Hz, 3H). ^{13}C NMR (126 MHz, $CDCl_3$) δ 175.87, 64.07, 32.61, 19.75, 17.92. HRMS (ESI) m/z: calcd for $C_5H_{10}O_2Cl$ $[M+H]^+$ 137.0364, found 137.0363.

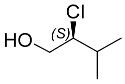

(S)-2-chloro-3-methylbutan-1-ol ((S)-**11**) (CAS 82378-45-8)

Following the similar procedure for (R) -2-chloro-3-methylbutan-1-ol ((R)-**11**), the reduction of (S)-2-chloro-3-methylbutanoic acid ((S)-**10**) (2.00 g, 14.64 mmol) with $LiAlH_4$ (0.83 g, 21.96 mmol) provided (S)-2-chloro-3-methylbutan-1-ol ((S)-**11**) (1.15 g, 64% yield) as a pale yellow liquid. $[\alpha]_D^{25}$ = +0.83 (c = 2.42, $CHCl_3$). Lit.[11] $[\alpha]_D^{23}$ = +0.45 (c = 1.80, $CHCl_3$). ^1H NMR (500 MHz, $CDCl_3$) δ 3.94 – 3.91 (m, 1H), 3.84 – 3.79 (m, 1H), 3.76 – 3.71 (m, 1H), 2.09 – 2.03 (m, 1H), 2.02 – 1.99 (m, 1H), 1.04 (d, J = 6.8 Hz, 3H), 1.02 (d, J = 6.7 Hz, 3H). ^{13}C NMR (126 MHz, $CDCl_3$) δ 72.03, 65.57, 31.60, 20.10, 18.28. HRMS (ESI) m/z: calcd for $C_5H_{12}OCl$ $[M+H]^+$ 123.0571, found 123.0562.

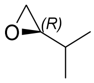

(R)-2-isopropyloxirane ((R)-**12**) (CAS 82378-47-0)

Following the similar procedure for (S)-2-isopropyloxirane ((S)-**12**), the cyclization of (R)-2-chloro-3-methylbutan-1-ol ((R)-**11**) (1.28 g, 10.44 mmol) with powdered KOH (1.29 g, 22.99 mmol) provided (R)-2-isopropyloxirane ((R)-**12**) (0.62 g, 69% yield) as a colorless liquid. $[\alpha]_D^{25}$= –3.36 (c = 2.14, $CHCl_3$), Lit.[8] $[\alpha]_D^{26}$ = –6.2 (c = 1.05, $CHCl_3$). ^1H NMR (500 MHz, $CDCl_3$) δ 2.73 – 2.69 (m, 2H), 2.51 (dt, J = 4.8, 3.0 Hz, 1H), 1.51 – 1.47 (m, 1H), 1.04 (d, J = 6.8 Hz, 3H), 0.96 (d, J =

6.9 Hz, 3H). ^{13}C NMR (126 MHz, $CDCl_3$) δ 57.76, 46.21, 30.91, 19.13, 18.25. HRMS (ESI) m/z: calcd for $C_5H_{10}OK$ $[M+K]^+$ 125.0363, found 125.0356.

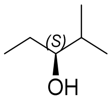

(*S*)-2-methylpentan-3-ol ((*S*)-**13**) (CAS 70492-65-8)

Following the similar procedure for (*R*)-2-methylpentan-3-ol ((*R*)-**13**), the ring opening of (*R*)-2-isopropyloxirane ((*R*)-**12**) (0.20 g, 2.32 mmol) with MeLi (3.5 mL, 1.6 M in Et_2O, 5.6 mmol) catalyzed by CuCN (0.25 g, 2.79 mmol) provided (*S*)-2-methylpentan-3-ol ((*S*)-**13**) (0.12 g, 51% yield, >99% ee, determined by chiral HPLC of its 3,5-dinitrobenzoate) as a pale yellow liquid. ^1H NMR (500 MHz, $CDCl_3$) δ 3.30 – 3.26 (m, 1H), 1.72 – 1.62 (m, 1H), 1.56 – 1.50 (m, 1H), 1.44 – 1.35 (m, 1H), 1.28 (br s, 1H), 0.96 (t, *J* = 7.4 Hz, 3H), 0.92 (d, *J* = 4.5 Hz, 3H), 0.91 (d, *J* = 4.5 Hz, 3H). ^{13}C NMR (126 MHz, $CDCl_3$) δ 78.38, 33.20, 27.09, 19.08, 17.22, 10.44. HRMS (ESI) m/z: calcd for $C_6H_{14}OK$ $[M+K]^+$ 141.0676, found 141.0665.

4. Research on the Enantiomeric Purity of Chiral Alcohols

Scheme S4. Synthesis of Mosher esters **18**.

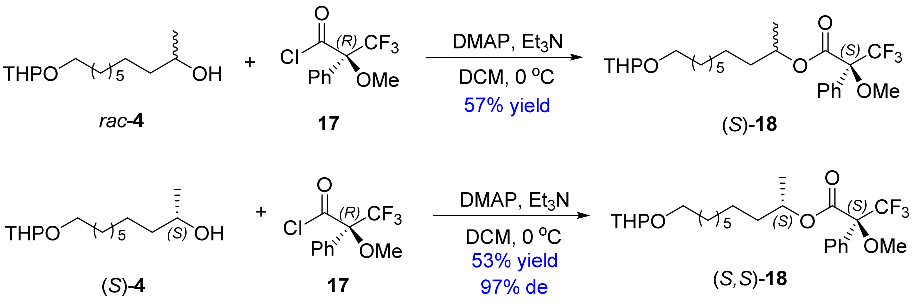

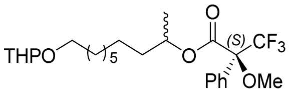

10-((tetrahydro-2*H*-pyran-2-yl)oxy)decan-2-yl (2*S*)-3,3,3-trifluoro-2-methoxy-2-phenylpropanoate ((*S*)-**18**) (new compound) [12]

Under argon atmosphere, DMAP (23.0 mg, 0.19 mmol) was added to a 10 mL Schlenk tube at room temperature, followed by the addition of alcohol *rac*-**4** (50.0 mg, 0.19 mmol), DCM (2 mL) and Et_3N (96.0 mg, 0.95 mmol). The resulting mixture was stirred for 5 min and cooled to 0 ^oC, then (*R*)-3,3,3-trifluoro-2-methoxy-2-phenylpropanoyl chloride (**17**) (48.0 mg, 0.19 mmol) was added. After the reaction solution was allowed to warm to room temperature and maintained for 1 h, it was quenched with saturated NH_4Cl solution (5 mL). The aqueous phase was separated and extracted with Et_2O (3 ×10 mL). The extracts were combined with the organic phase and washed with saturated brine (20 mL), then dried over anhydrous Na_2SO_4. The solvent was evaporated under the reduce pressure and the residue was purified by column chromatography on silica gel (petroleum ether/EtOAc = 10:1) to afford 10-((tetrahydro-2*H*-pyran-2-yl)oxy)decan-2-yl (2*S*)-3,3,3-trifluoro-2-methoxy-2-phenylpropanoate ((*S*)-**18**) (51.0 mg, 57% yield) as a colorless oil. ^1H NMR (500 MHz, $CDCl_3$) δ 7.56 – 7.53 (m, 2H), 7.41 – 7.39 (m, 3H), 5.15 – 5.13 (m, 1H), 4.57 (dd, *J* = 4.6, 2.8 Hz, 1H), 3.89 – 3.85 (m, 1H), 3.75 – 3.71 (m, 1H), 3.57 (d, *J* = 1.4 Hz, 1.5H), 3.55 (d, *J* = 1.4 Hz, 1.5H), 3.51 – 3.49 (m, 1H), 3.38 (dt, *J* = 9.6, 6.7 Hz, 1H), 1.85 – 1.82 (m, 1H), 1.74 – 1.69 (m, 2H), 1.61 – 1.50 (m, 7H), 1.37 – 1.20 (m, 13H). ^{13}C NMR (126 MHz, $CDCl_3$) δ 166.29, 132.74, 132.57, 129.62, 128.46, 128.43, 127.50, 127.35, 123.52 (q, *J* = 289.0 Hz), 123.50 (q, *J* = 289.0 Hz), 99.01, 84.60 (q, *J* = 24.8 Hz), 74.31, 74.12, 67.78, 62.50, 55.50, 35.71, 35.68, 30.92, 29.86, 29.53, 29.47, 29.37, 26.33, 25.63, 25.44, 19.98, 19.85, 19.60. ^{19}F NMR (471 MHz, $CDCl_3$) δ -71.43, -71.48. HRMS (ESI) m/z: calcd for $C_{25}H_{37}O_5F_3Na$ $[M+Na]^+$ 497.2485, found 497.2496.

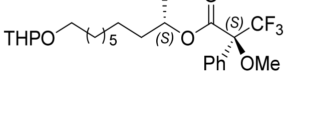

10-((tetrahydro-2*H*-pyran-2-yl)oxy)decan-2-yl (2*S*)-3,3,3-trifluoro-2-methoxy-2-phenylpropanoate ((*S*, *S*)-**18**) (new compound)

Following the similar procedure for Mosher ester (*S*)-**18**, the esterification of chiral alcohol (*S*)-**4** (50.0 mg, 0.19 mmol) with (*R*)-3,3,3-trifluoro-2-methoxy-2-phenylpropanoyl chloride (**17**) (48.0 mg, 0.19 mmol) provided 10-((tetrahydro-2*H*-pyran-2-yl)oxy)decan-2-yl (2*S*)-3,3,3-trifluoro-2-methoxy-2-phenylpropanoate ((*S*, *S*)-**18**) (48.0 mg, 53% yield, 97 % de, determined

by its ^{1}H NMR spectrum) as a colorless oil. $[\alpha]_D^{22}$ = –14.82 (c = 2.24, $CHCl_3$). ^{1}H NMR (500 MHz, $CDCl_3$) δ 7.53 (dd, *J* = 6.9, 3.0 Hz, 2H), 7.42 – 7.38 (m, 3H), 5.15 – 5.12 (m, 1H), 4.57 (dd, *J* = 4.5, 2.8 Hz, 1H), 3.87 (ddd, *J* = 11.0, 7.5, 3.2 Hz, 1H), 3.73 (dt, *J* = 9.6, 6.9 Hz, 1H), 3.55 (d, *J* = 1.4 Hz, 3H), 3.51 – 3.48 (m, 1H), 3.40 – 3.36 (m, 1H), 1.84 – 1.82 (m, 1H), 1.72 – 1.67 (m, 2H), 1.60 – 1.50 (m, 7H), 1.35 – 1.24 (m, 13H). ^{13}C NMR (126 MHz, $CDCl_3$) δ 166.29, 132.57, 129.62, 128.46, 127.50, 123.49 (q, *J* = 289.2 Hz), 99.01, 84.70 (q, *J* = 27.5 Hz), 74.32, 67.78, 62.51, 55.50, 35.71, 30.92, 29.86, 29.53, 29.47, 29.37, 26.33, 25.63, 25.44, 19.85, 19.61. ^{19}F NMR (471 MHz, $CDCl_3$) δ -71.43. HRMS (ESI) m/z: calcd for $C_{25}H_{37}O_5F_3Na$ $[M+Na]^+$ 497.2485, found 497.2494.

Scheme S5. Synthesis of 3,5-dinitrobenzoates **20**.

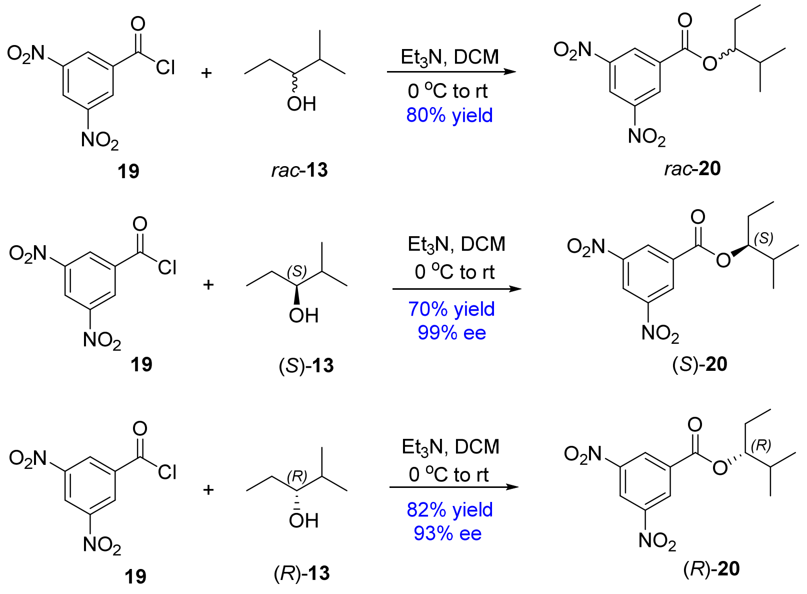

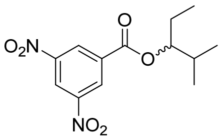

2-methylpentan-3-yl 3,5-dinitrobenzoate (*rac*-**20**) [13]

Under an argon atmosphere, alcohol *rac*-**13** (25.0 mg, 0.24 mmol) and DCM (2 mL) were added to a 10 mL Schlenk tube at room temperature, followed by the addition of Et_3N (54.0 mg, 0.53 mmol). The resulting solution was stirred for 5 min and cooled to 0 °C, then 3,5-dinitrobenzoyl chloride (67.0 mg, 0.29 mmol) was added. After the reaction solution was

warmed to room temperature and stirred for 3 h, it was quenched with saturated NH_4Cl solution (2 mL). The aqueous phase was separated and extracted with Et_2O (3 × 2 mL). The extracts were combined with the organic phase and washed with saturated brine (5 mL), then dried over anhydrous Na_2SO_4. The solvent was evaporated under the reduce pressure and the residue was purified by column chromatography on silica gel (petroleum ether/ethyl acetate = 10:1) to afford 2-methylpentan-3-yl 3,5-dinitrobenzoate (*rac*-**20**) (57.0 mg, 80% yield) as a pale yellow solid. Mp 86.6 – 87.1 ^{o}C. ^{1}H NMR (500 MHz, $CDCl_3$) δ 9.23 (t, *J* = 2.2 Hz, 1H), 9.16 (d, *J* = 2.2 Hz, 2H), 5.06 – 5.03 (m, 1H), 2.07 – 2.02 (m, 1H), 1.81 – 1.76 (m, 2H), 1.00 (dd, *J* = 6.9, 4.6 Hz, 6H), 0.96 (t, *J* = 7.4 Hz, 3H). ^{13}C NMR (126 MHz, $CDCl_3$) δ 162.53, 148.85, 134.60, 129.51, 122.38, 83.56, 31.32, 24.32, 18.83, 17.90, 10.11. HRMS (ESI) m/z: calcd for $C_{13}H_{17}O_6N_2$ $[M+H]^+$ 297.1081, found 297.1086.

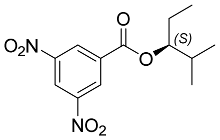

(*S*)-2-methylpentan-3-yl 3,5-dinitrobenzoate ((*S*)-**20**)（CAS 89117-70-4）

Following the similar procedure for 3,5-dinitrobenzoate *rac*-**20**, the esterification of chiral alcohol (*S*)-**13** (25.0 mg, 0.24 mmol) with 3,5-dinitrobenzoyl chloride (67.0 mg, 0.29 mmol) provided (*S*)-2-methylpentan-3-yl 3,5-dinitrobenzoate ((*S*)-**20**) (50.0 mg, 70% yield, >99% ee) as a pale yellow solid. Ee was determined by chiral HPLC (Daicel Chiralcel OJ-H column, 254 nm, *n*-hexane/2-propanol = 9 9.5:0.5, 1.0 mL/min, t_r = 20.36 min (*S*)). Mp 86.6 – 87.1 ^{o}C. $[\alpha]_D^{25}$ = +4.68 (c =1.37, $CHCl_3$). ^{1}H NMR (500 MHz, $CDCl_3$) δ 9.23 (t, *J* = 2.1 Hz, 1H), 9.16 (d, *J* = 2.1 Hz, 2H), 5.06 – 5.03 (m, 1H), 2.06 – 2.03 (m, 1H), 1.80 – 1.76 (m, 2H), 1.00 (dd, *J* = 6.8, 4.7 Hz, 6H), 0.96 (t, *J* = 7.4 Hz, 3H). ^{13}C NMR (126 MHz, $CDCl_3$) δ 162.53, 148.85, 134.60, 129.51, 122.38, 83.56, 31.32, 24.32, 18.83, 17.90, 10.11. HRMS (ESI) m/z: calcd for $C_{13}H_{17}O_6N_2$ $[M+H]^+$ 297.1081, found 297.1080.

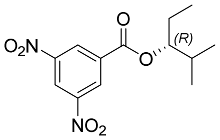

(*R*)-2-methylpentan-3-yl 3,5-dinitrobenzoate ((*R*)-**20**)（CAS 16551-30-7）

Following the similar procedure for 3,5-dinitrobenzoate *rac*-**20**, the esterification of chiral alcohol (*R*)-**13** (25.0 mg, 0.24 mmol) with 3,5-dinitrobenzoyl chloride (67.0 mg, 0.29 mmol) provided (*R*)-2-methylpentan-3-yl 3,5-dinitrobenzoate ((*R*)-**20**) (58.0 mg, 82% yield, 93% ee) as a pale yellow solid. Ee was determined by chiral HPLC (Daicel Chiralcel OJ-H column, 254 nm, *n*-hexane/2-propanol = 99.5:0.5, 1.0 mL/min, minor t_r = 20.49 min (*S*), major t_r = 22.65 min (*R*)). Mp 86.6 – 87.1 ^oC. ^1H NMR (500 MHz, $CDCl_3$) δ 9.23 (t, *J* = 1.9 Hz, 1H), 9.16 (d, *J* = 2.3 Hz, 2H), 5.07 – 5.03 (m, 1H), 2.09 – 2.04 (m, 1H), 1.78 – 1.75 (m, 2H), 1.01 (dd, *J* = 6.7, 4.6 Hz, 6H), 0.95 (t, *J* = 7.4, 3H). ^{13}C NMR (126 MHz, $CDCl_3$) δ 162.52, 148.83, 134.58, 129.48, 122.35, 83.53, 31.30, 24.30, 18.80, 17.87, 10.08. HRMS (ESI) m/z: calcd for $C_{13}H_{16}O_6N_2Na$ $[M+Na]^+$ 319.0901, found 319.0887.

5. ^{1}H, ^{13}C, ^{19}F NMR Spectra of the Products

Figure S1. ^{1}H NMR Spectrum of (*S*)-10-((tetrahydro-2H-pyran-2-yl)oxy)decan-2-ol ((*S*)-**4**) (500 MHz, $CDCl_3$)

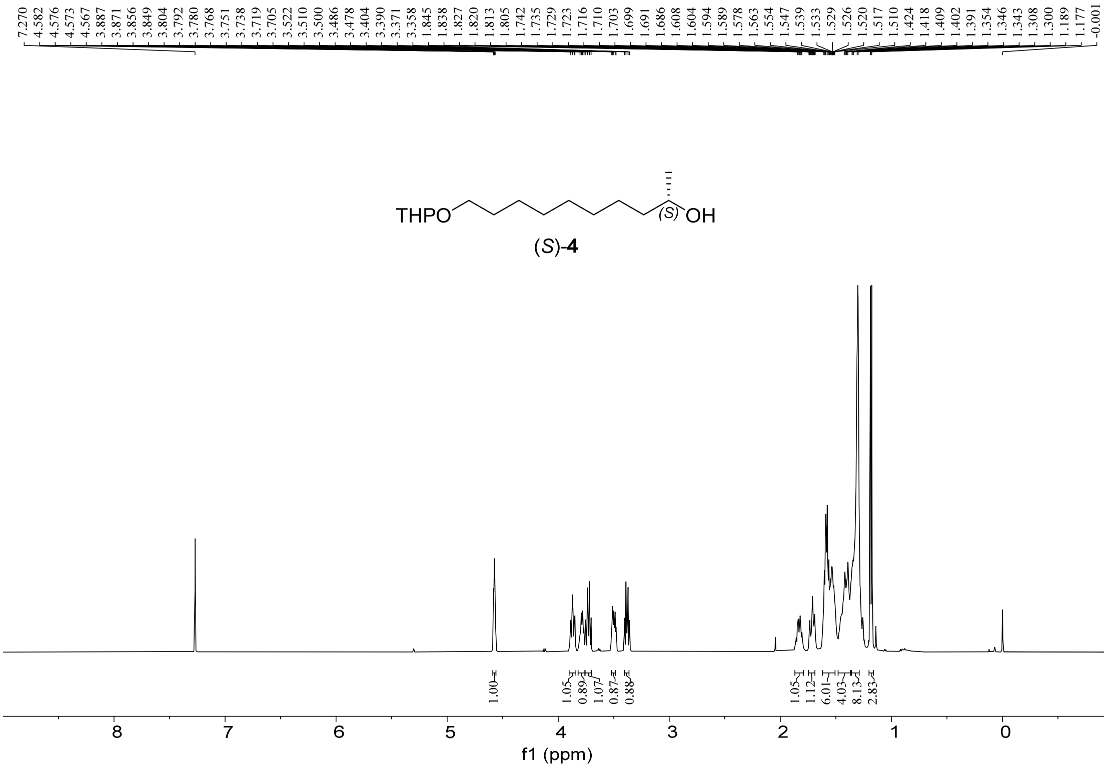

Figure S2. ^{13}C NMR Spectrum of (*S*)-10-((tetrahydro-2H-pyran-2-yl)oxy)decan-2-ol ((*S*)-**4**) (126 MHz, $CDCl_3$)

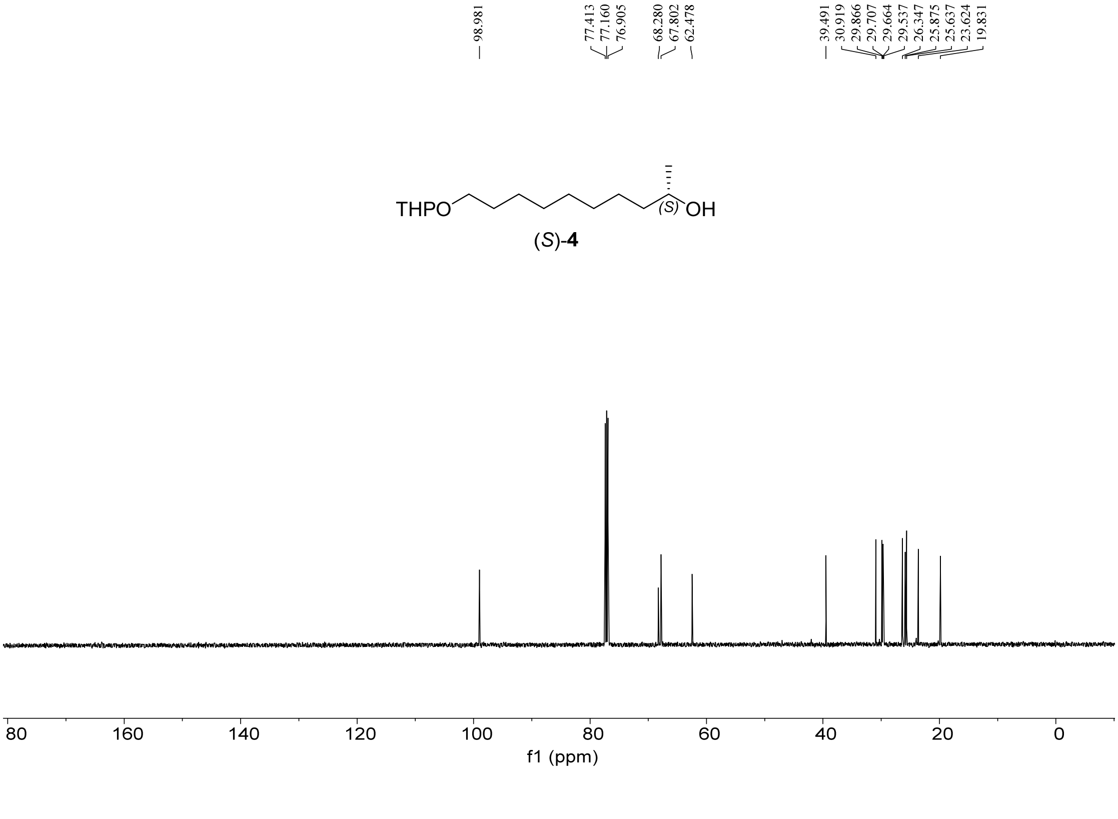

Figure S3. ^{1}H NMR Spectrum of (*S*)-10-((tetrahydro-2H-pyran-2-yl)oxy)decan-2-yl 4-methylbenzenesulfonate ((*S*)-**5**) (500 MHz, $CDCl_3$)

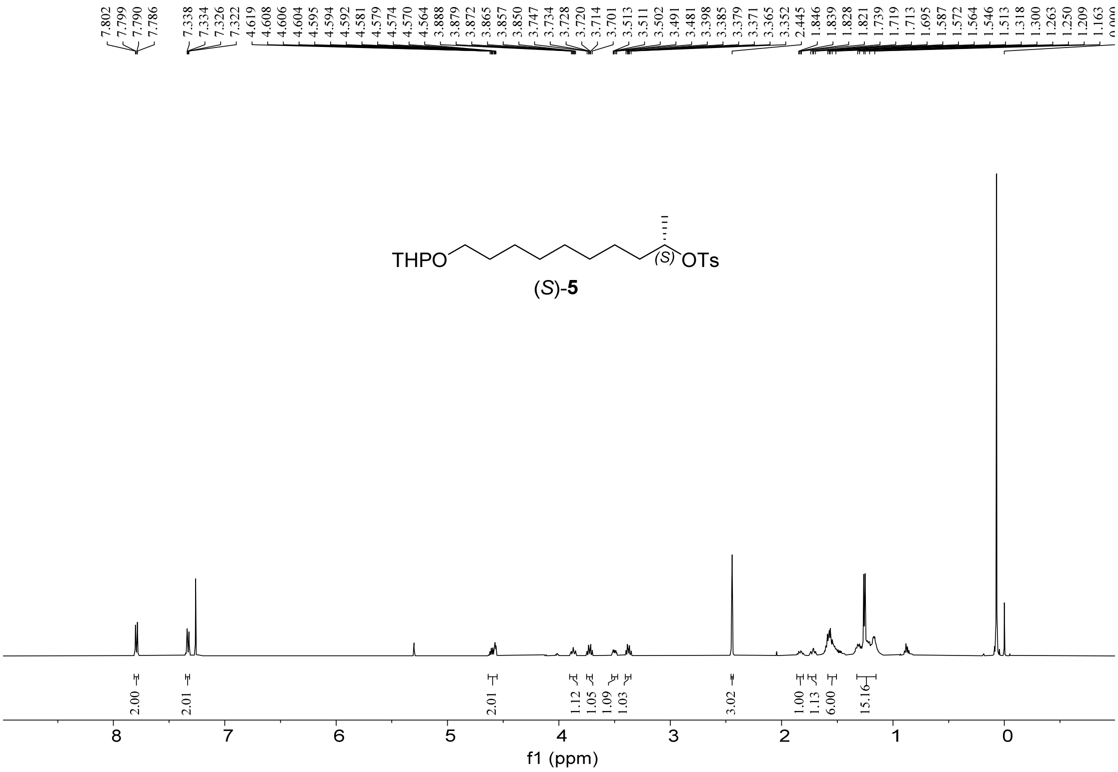

Figure S4. ^{13}C NMR Spectrum of (*S*)-10-((tetrahydro-2H-pyran-2-yl)oxy)decan-2-yl 4-methylbenzenesulfonate ((*S*)-**5**) (126 MHz, $CDCl_3$)

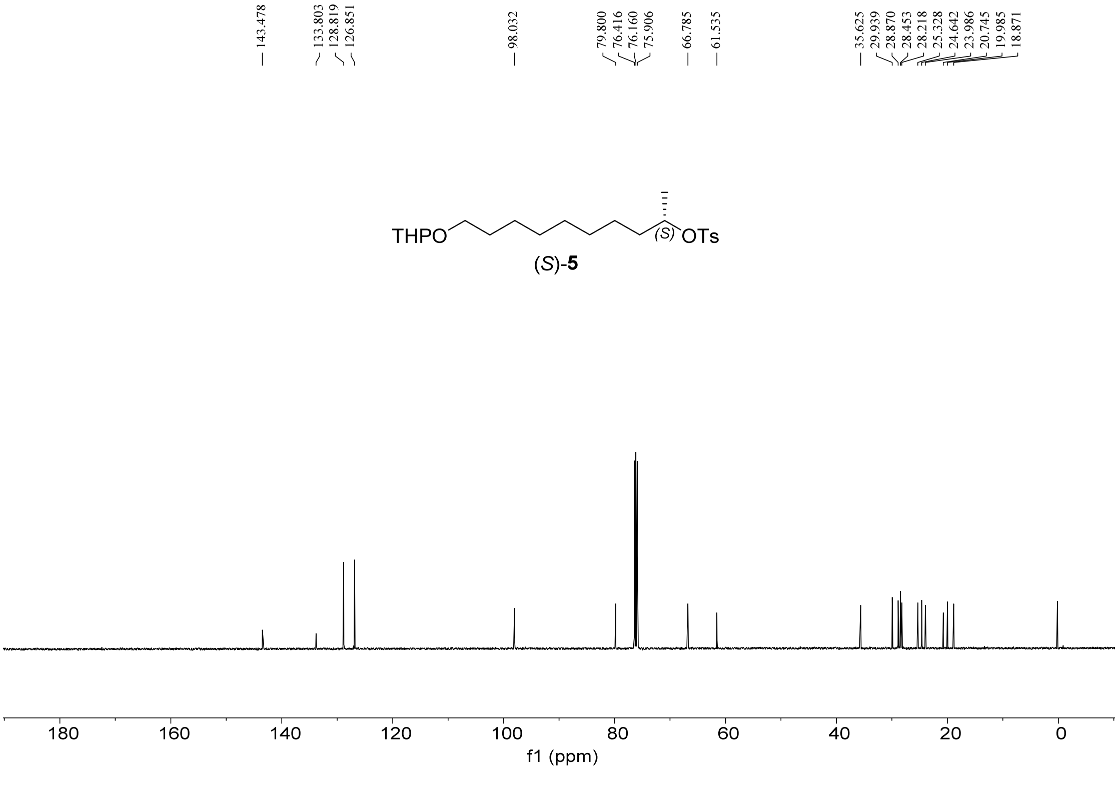

Figure S5. ^{1}H NMR Spectrum of 2-(((*R*)-9-methyldodec-11-en-1-yl)oxy)tetrahydro-2H-pyran ((*R*)-**6**) (500 MHz, $CDCl_3$)

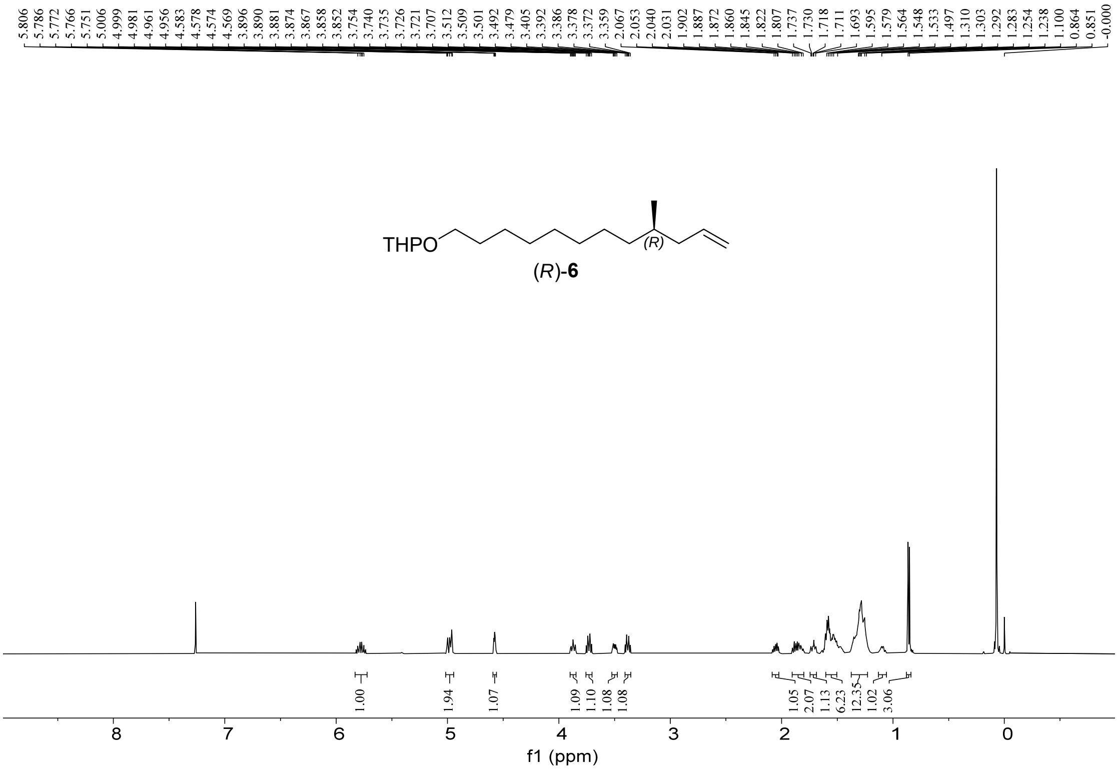

Figure S6. ^{13}C NMR Spectrum of 2-(((*R*)-9-methyldodec-11-en-1-yl)oxy)tetrahydro-2H-pyran ((*R*)-**6**) (126 MHz, $CDCl_3$)

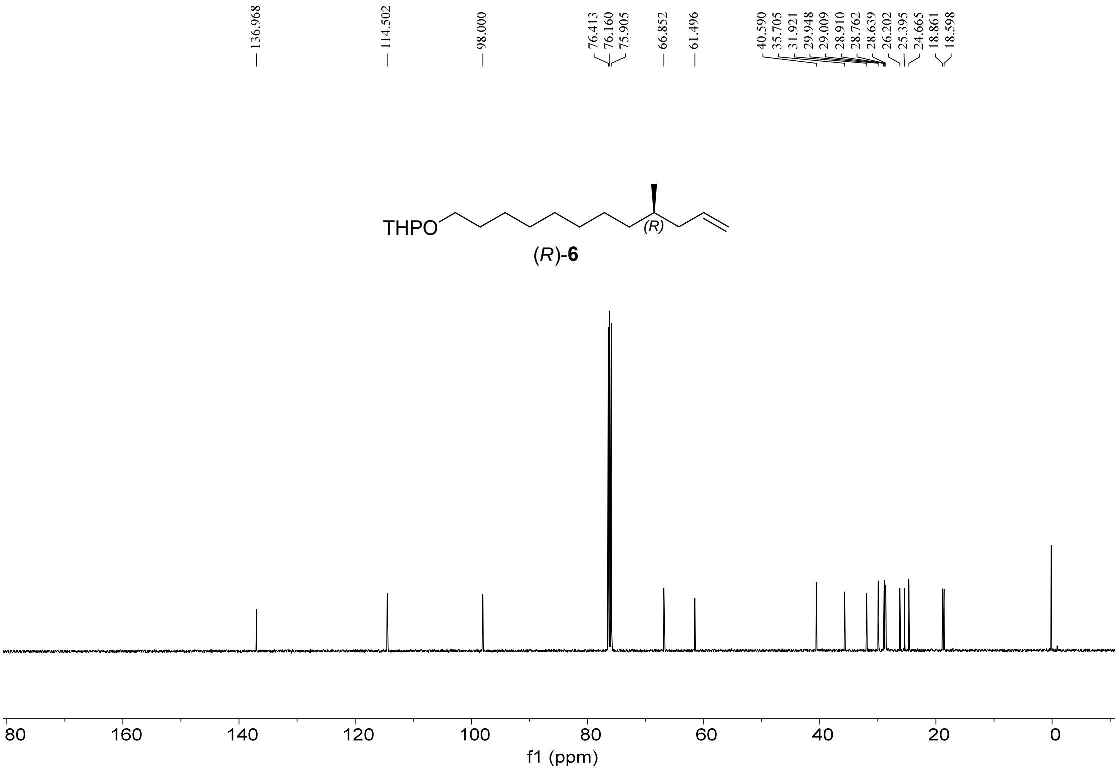

Figure S7. ^{1}H NMR Spectrum of (*R*)-9-methyldodec-11-en-1-ol ((*R*)-**7**) (500 MHz, $CDCl_3$)

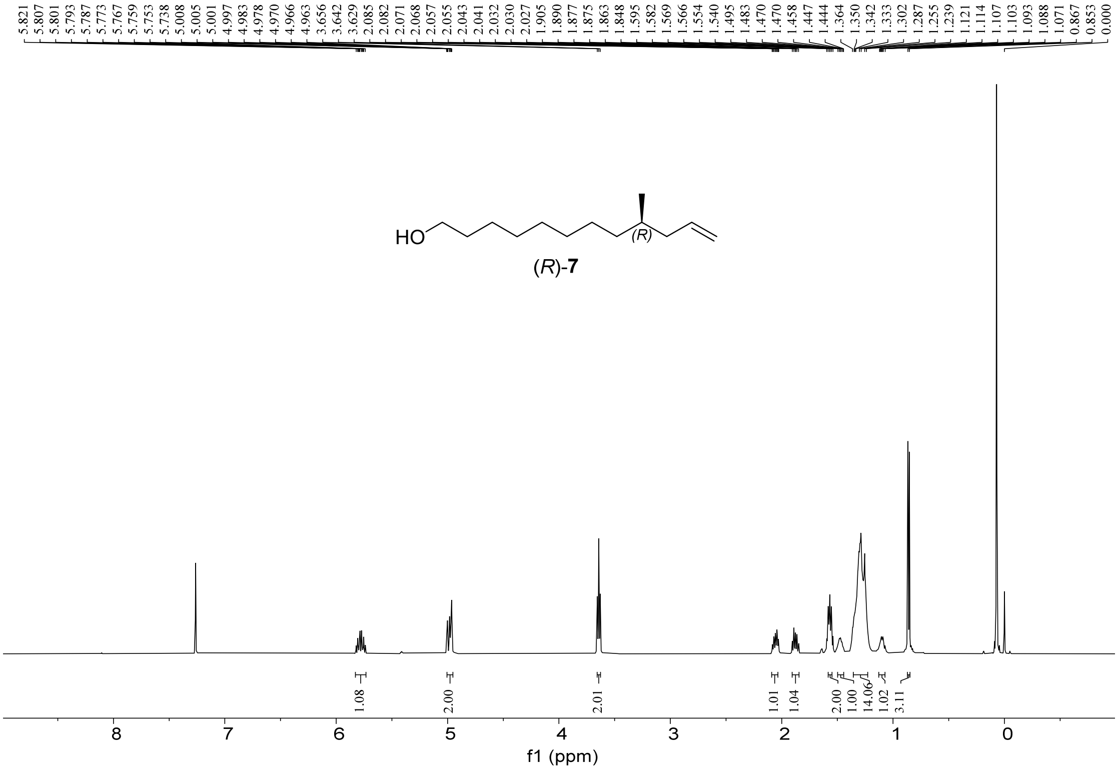

Figure S8. ^{13}C NMR Spectrum of (*R*)-9-methyldodec-11-en-1-ol ((*R*)-**7**) (126 MHz, $CDCl_3$)

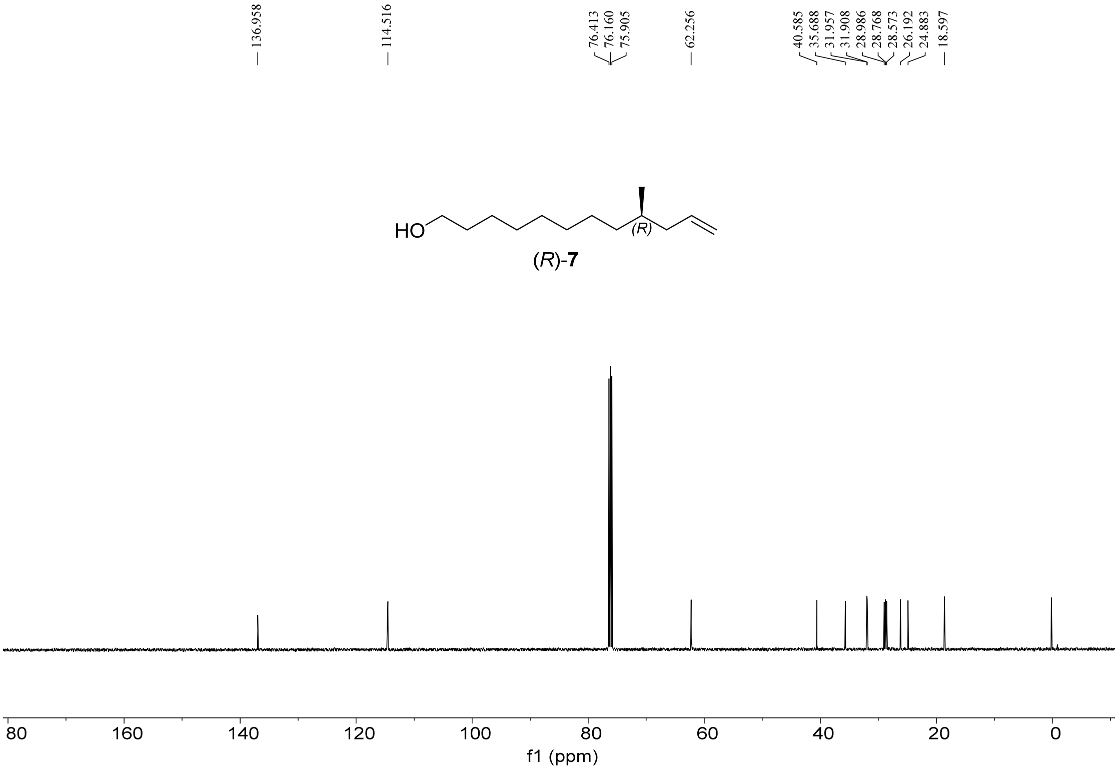

Figure S9. ^{1}H NMR Spectrum of (*R*)-9-methyldodec-11-en-1-yl 4-methylbenzenesulfonate ((*R*)-**8**) (500 MHz, CDCl3)

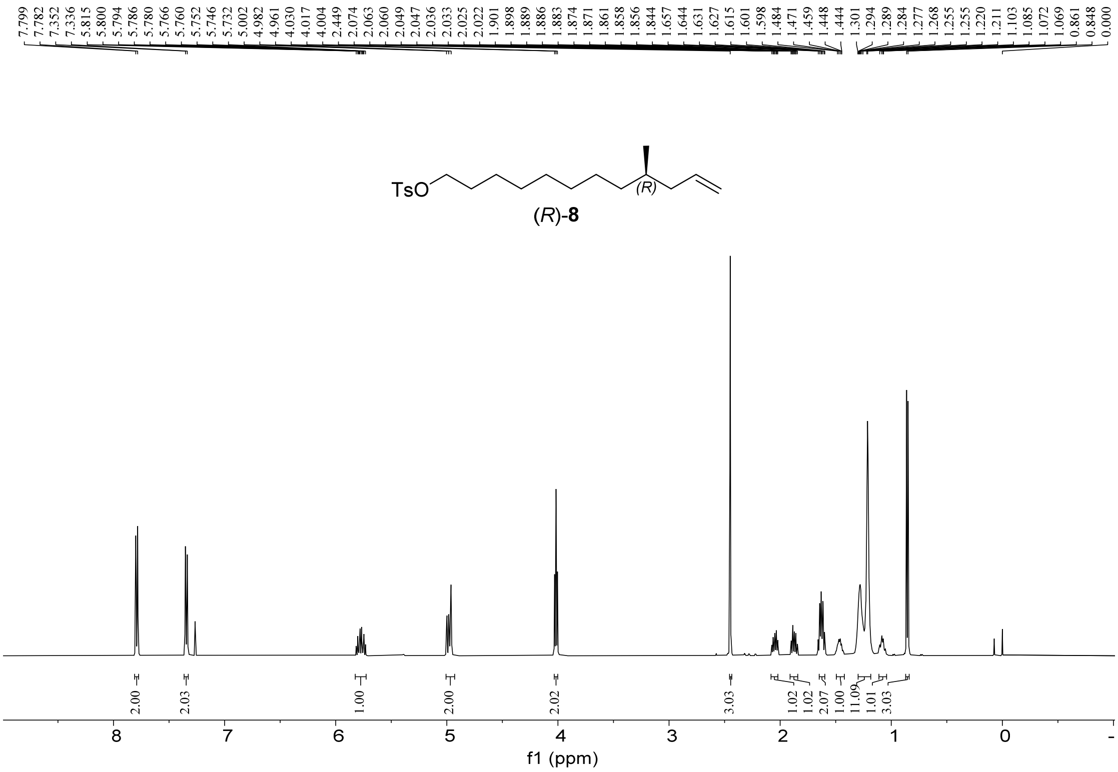

Figure S10. ^{13}C NMR Spectrum of (*R*)-9-methyldodec-11-en-1-yl 4-methylbenzenesulfonate ((*R*)-**8**) (126 MHz, CDCl3)

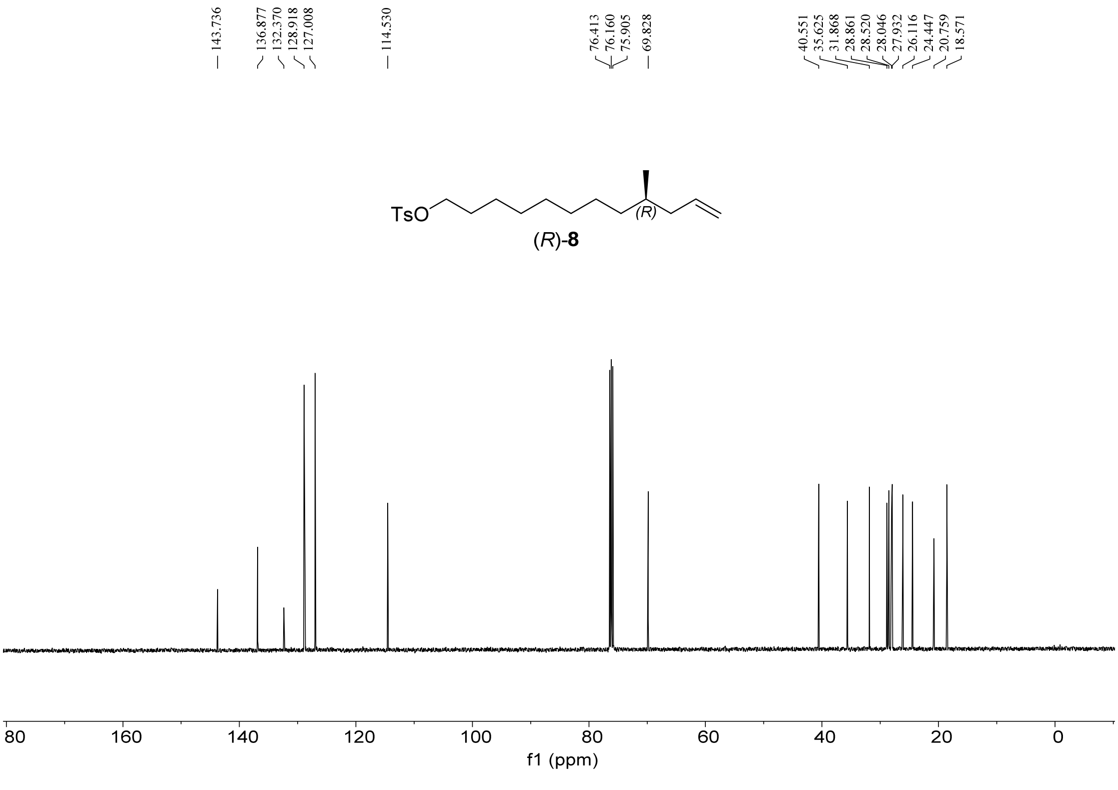

Figure S11. ^{1}H NMR Spectrum of (*R*)-2-chloro-3-methylbutanoic acid ((*R*)-**10**) (500 MHz, $CDCl_3$)

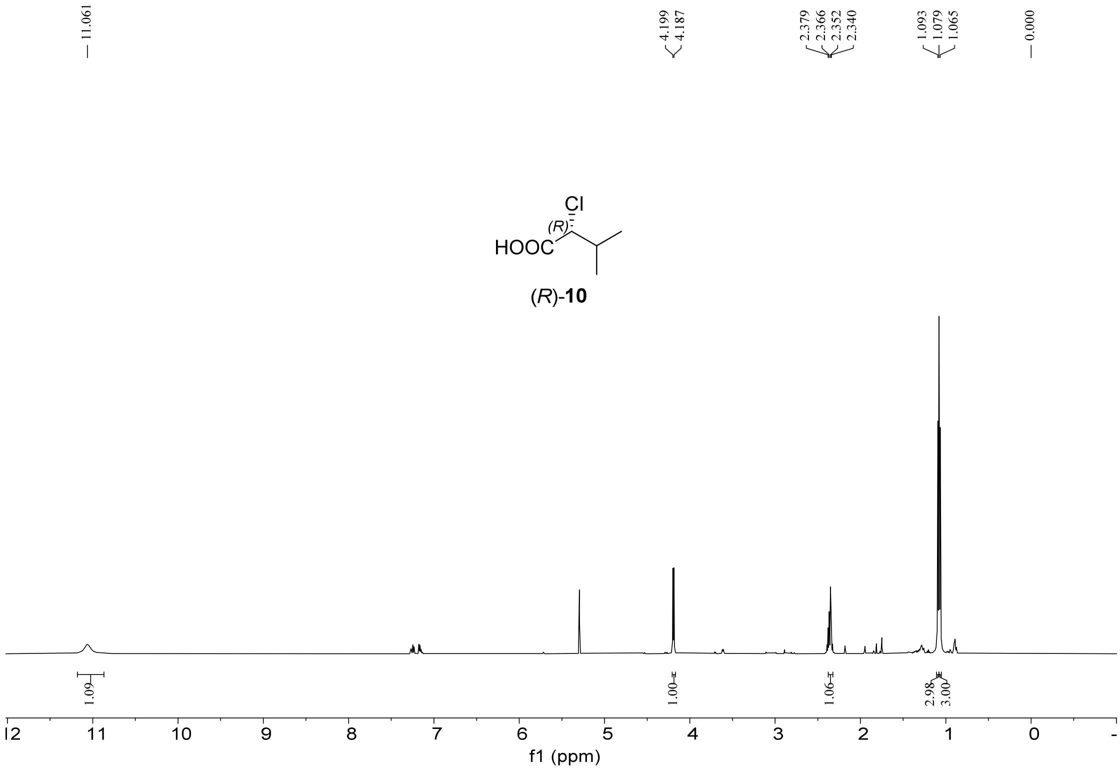

Figure S12. ^{13}C NMR Spectrum of (*R*)-2-chloro-3-methylbutanoic acid ((*R*)-**10**) (126 MHz, $CDCl_3$)

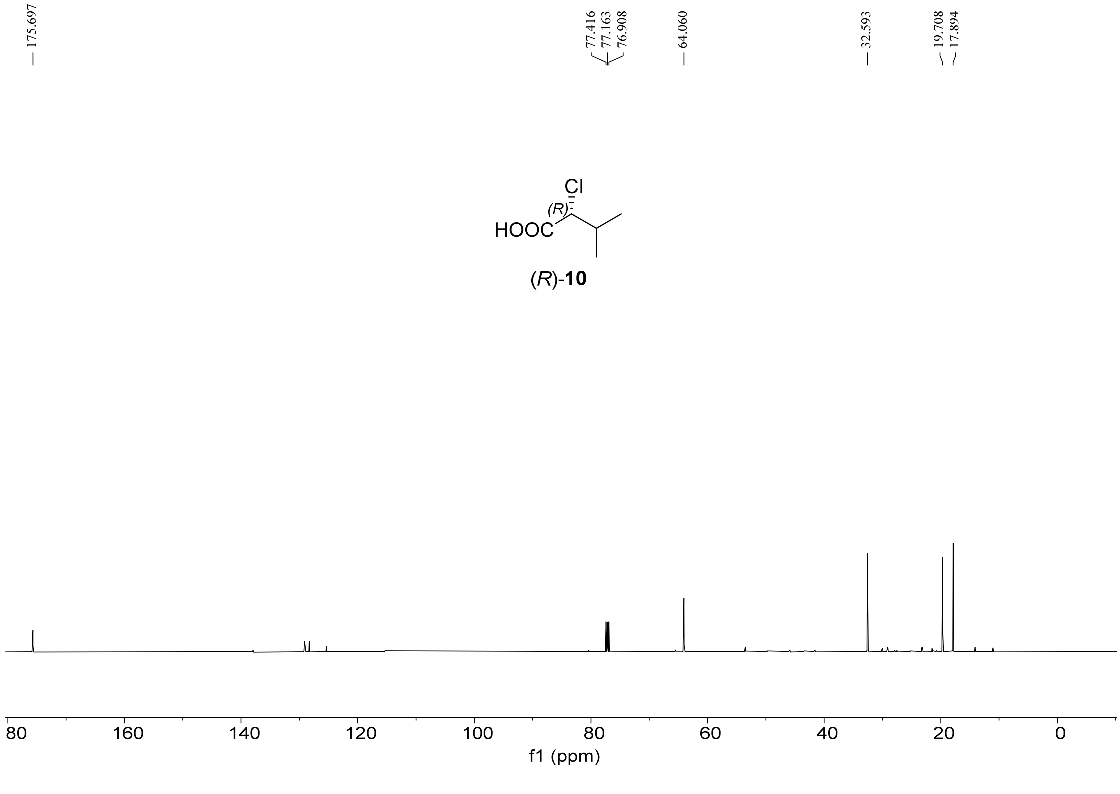

Figure S13. ^{1}H NMR Spectrum of (*R*)-2-chloro-3-methylbutan-1-ol ((*R*)-**11**) (500 MHz, CDCl3)

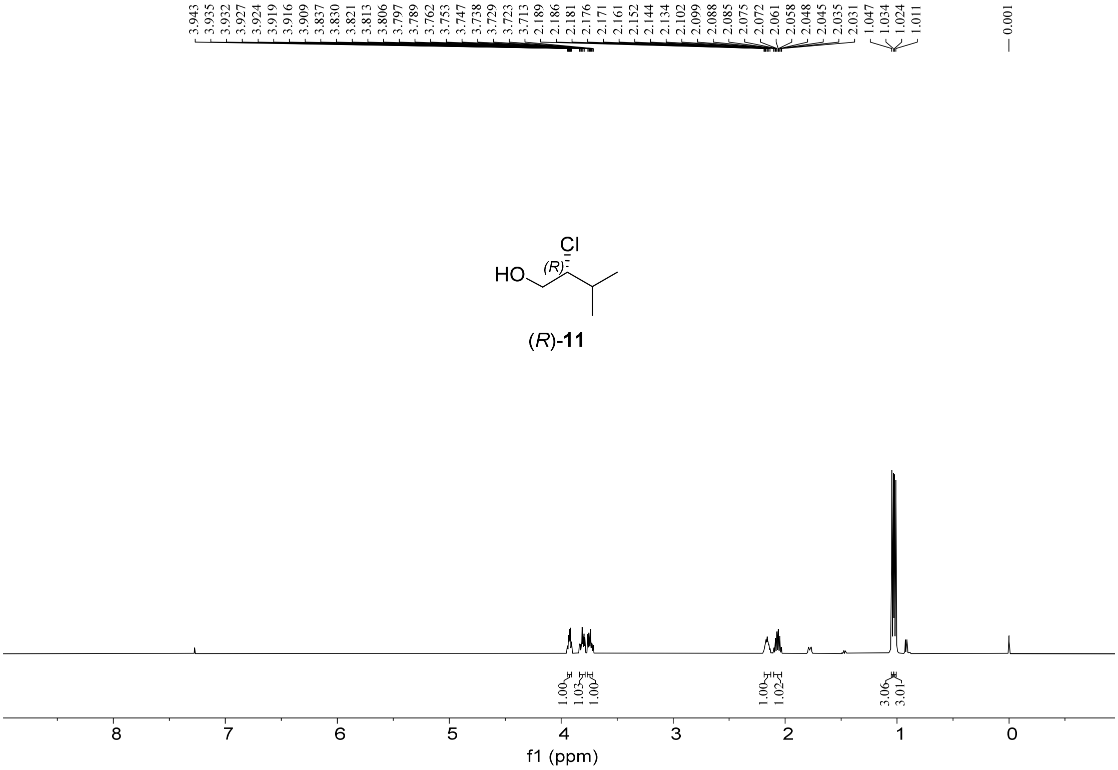

Figure S14. ^{13}C NMR Spectrum of (*R*)-2-chloro-3-methylbutan-1-ol ((*R*)-**11**) (126 MHz, CDCl3)

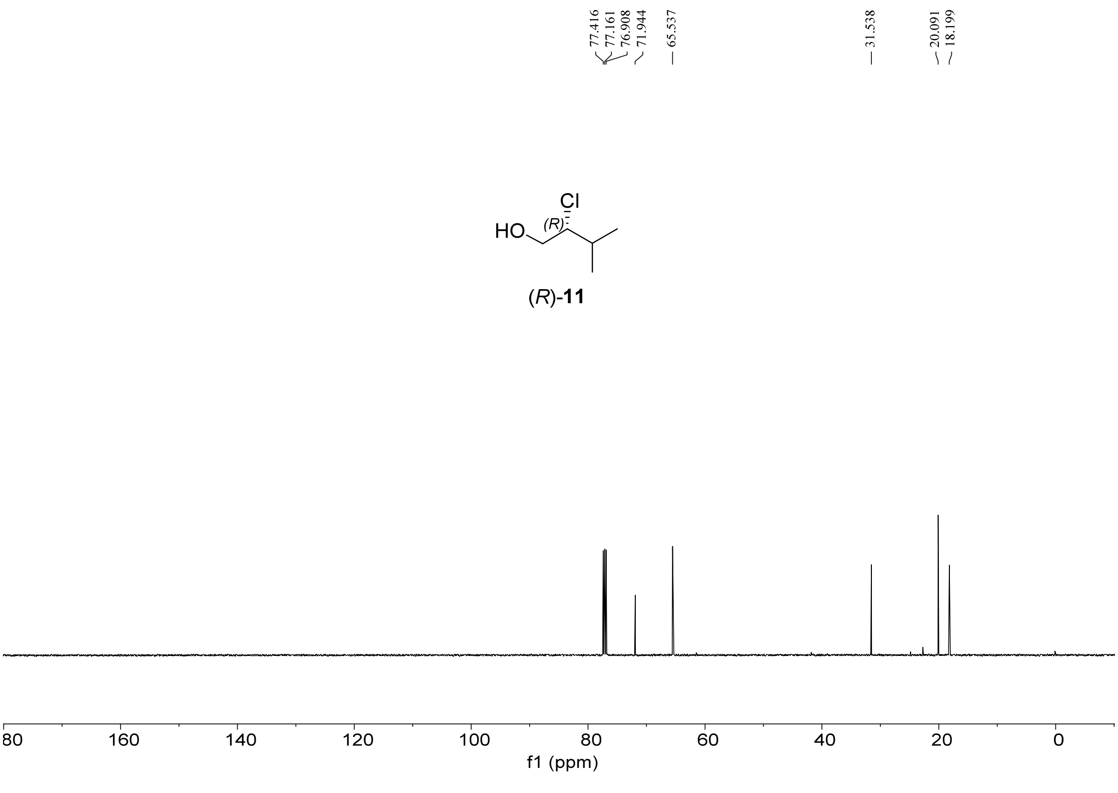

Figure S15. ^{1}H NMR Spectrum of (*S*)-2-isopropyloxirane ((*S*)-**12**) (500 MHz, $CDCl_3$)

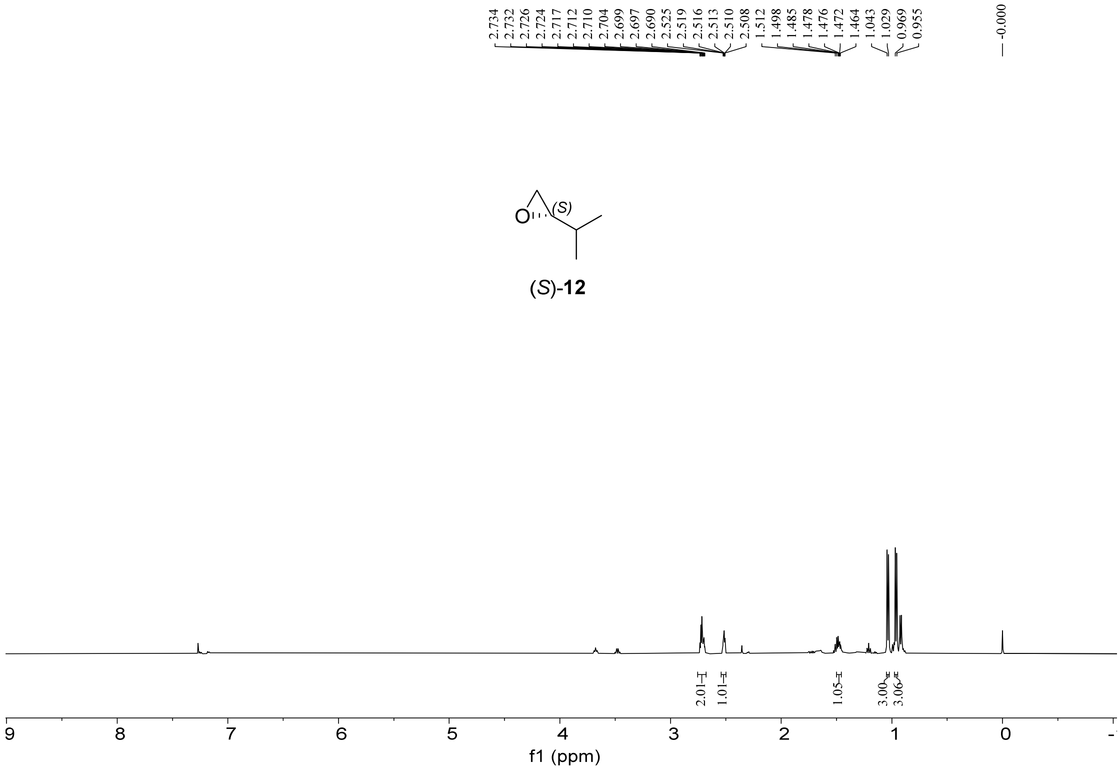

Figure S16. ^{13}C NMR Spectrum of (*S*)-2-isopropyloxirane ((*S*)-**12**) (126 MHz, $CDCl_3$)

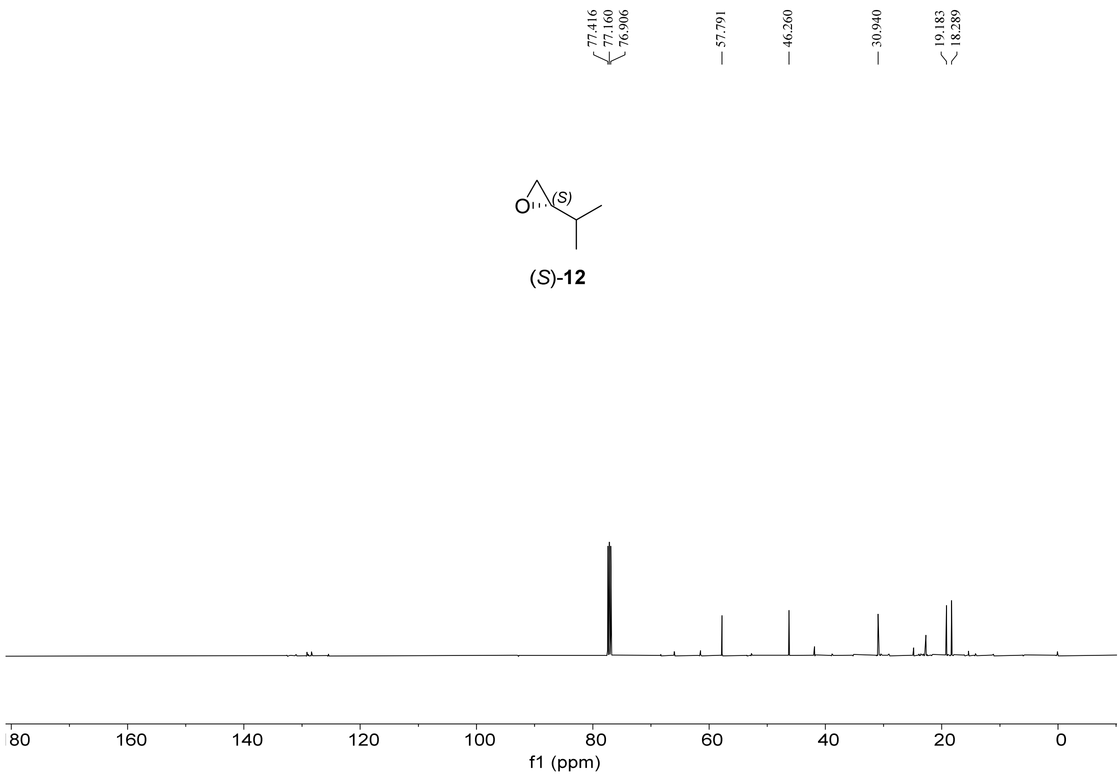

Figure S17. ^{1}H NMR Spectrum of (*R*)-2-methylpentan-3-ol ((*R*)-**13**) (500 MHz, $CDCl_3$)

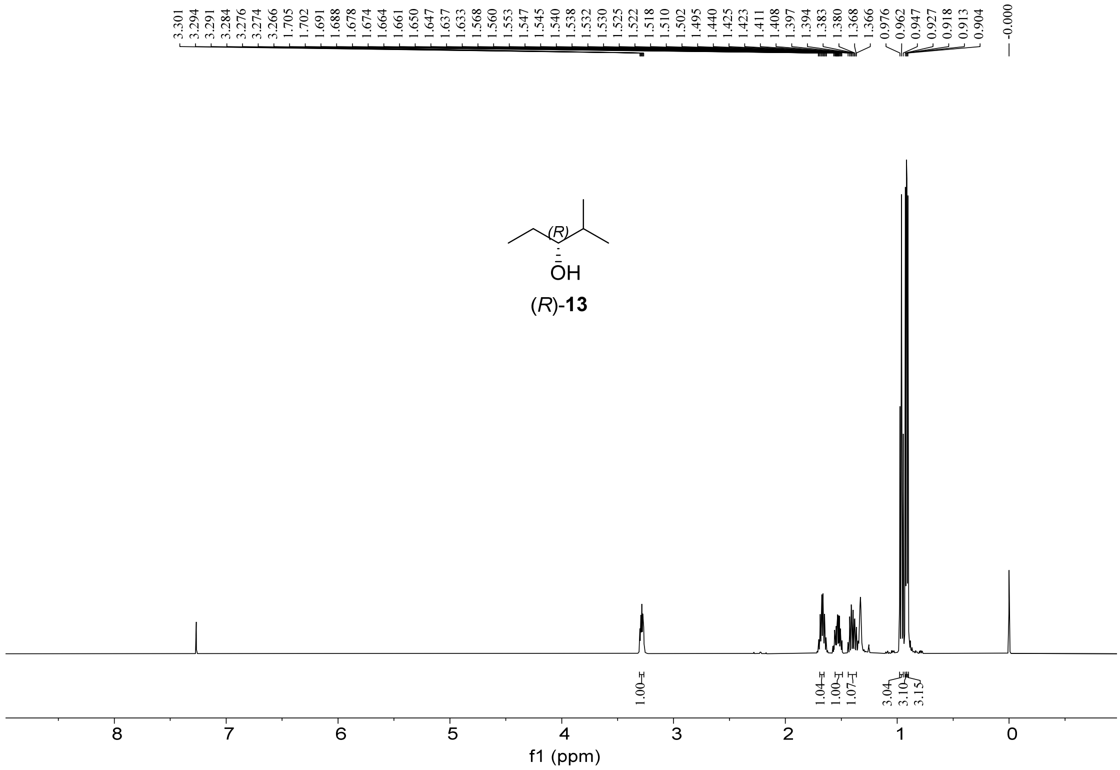

Figure S18. ^{13}C NMR Spectrum of (*R*)-2-methylpentan-3-ol ((*R*)-**13**) (126 MHz, $CDCl_3$)

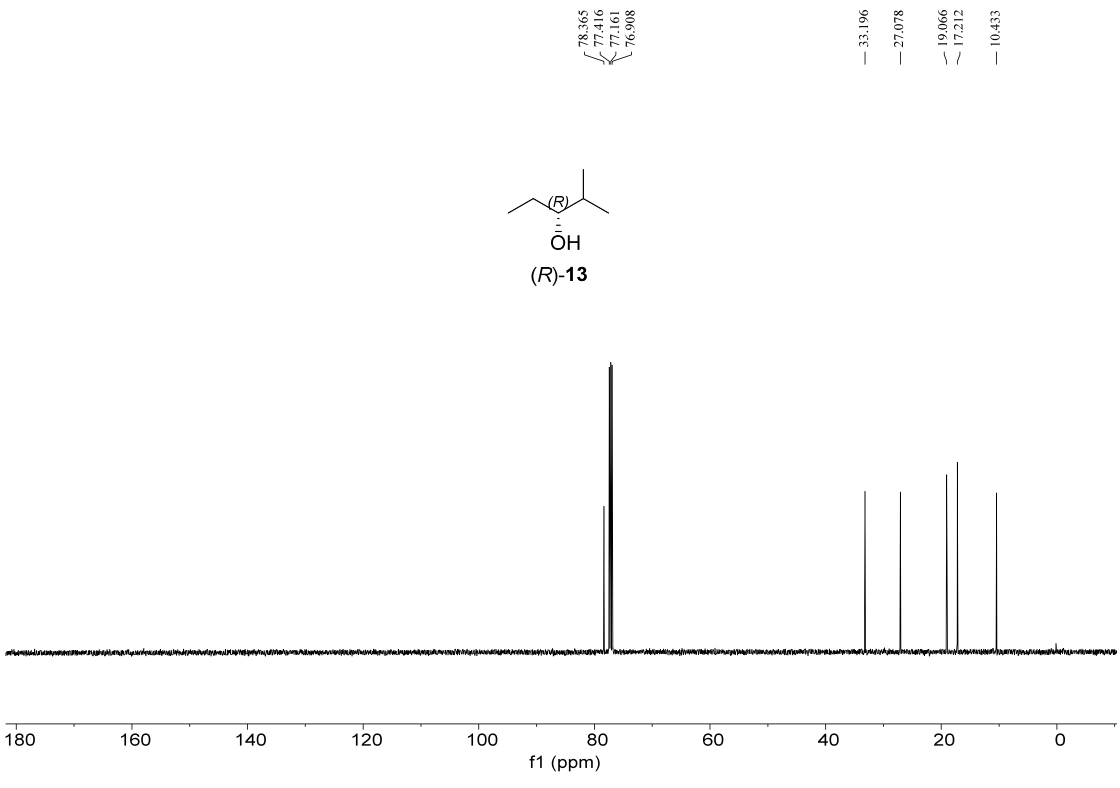

Figure S19. ^{1}H NMR Spectrum of (*S*)-2-chloro-3-methylbutanoic acid ((*S*)-**10**) (500 MHz, $CDCl_3$)

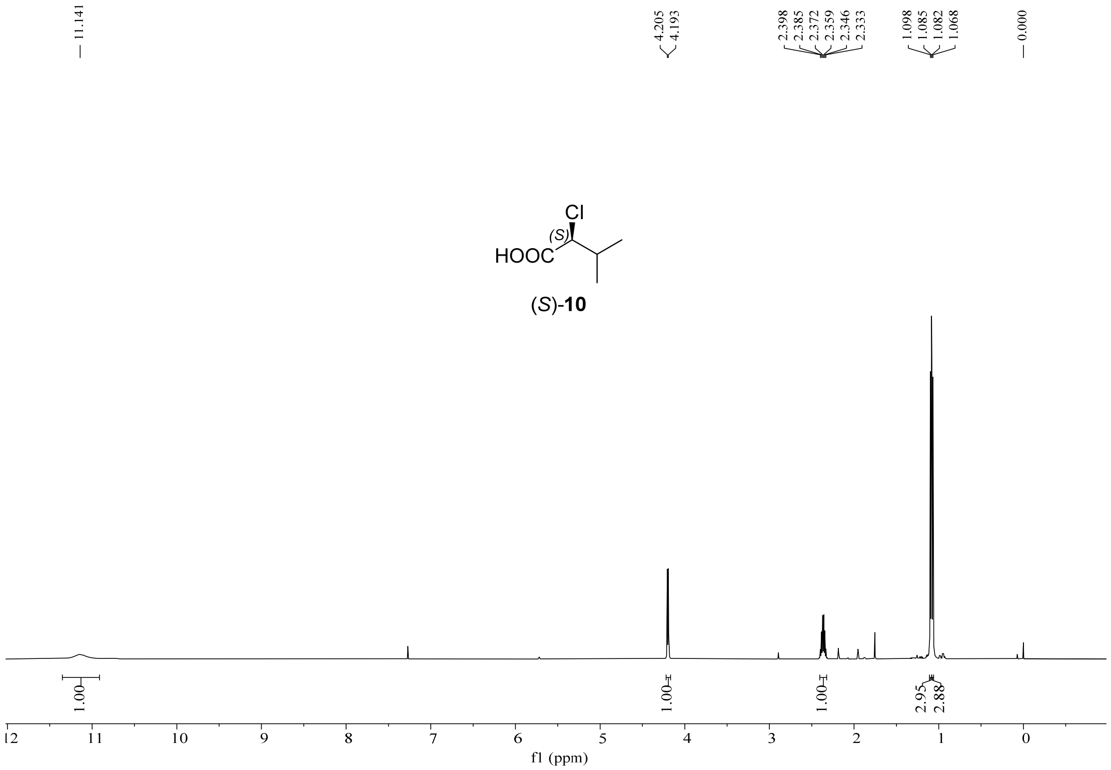

Figure S20. ^{13}C NMR Spectrum of (*S*)-2-chloro-3-methylbutanoic acid ((*S*)-**10**) (126 MHz, $CDCl_3$)

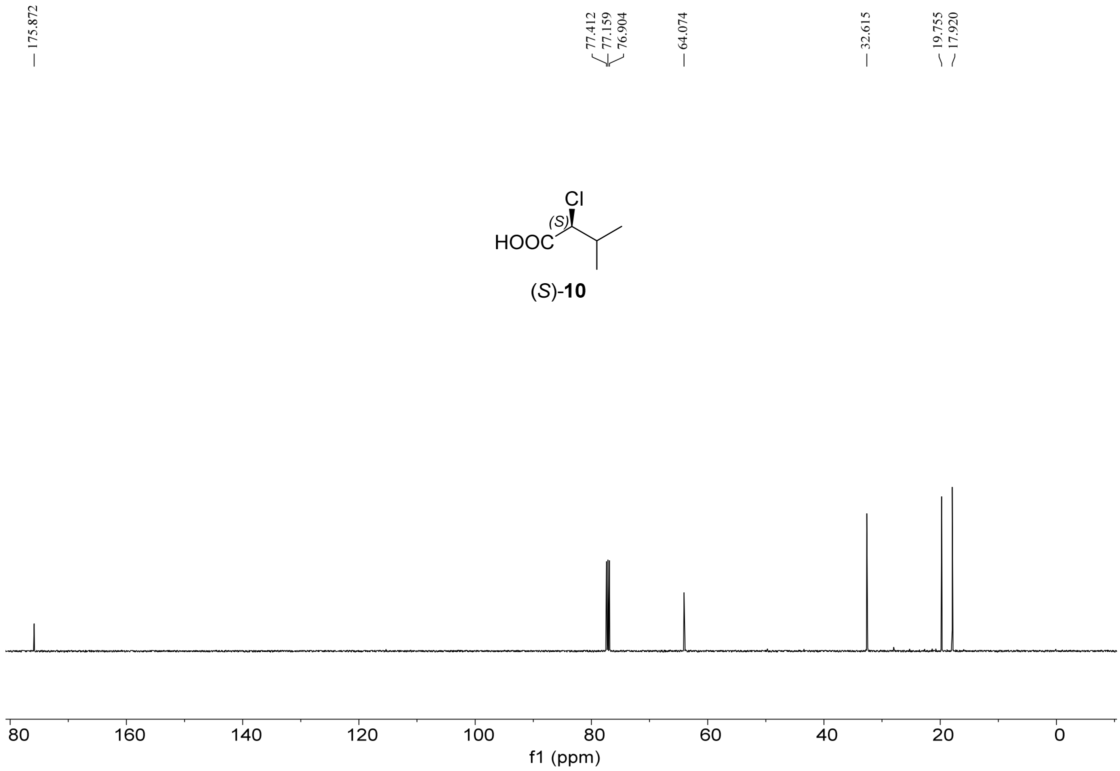

Figure S21. ^{1}H NMR Spectrum of (*S*)-2-chloro-3-methylbutan-1-ol ((*S*)-**11**) (500 MHz, $CDCl_3$)

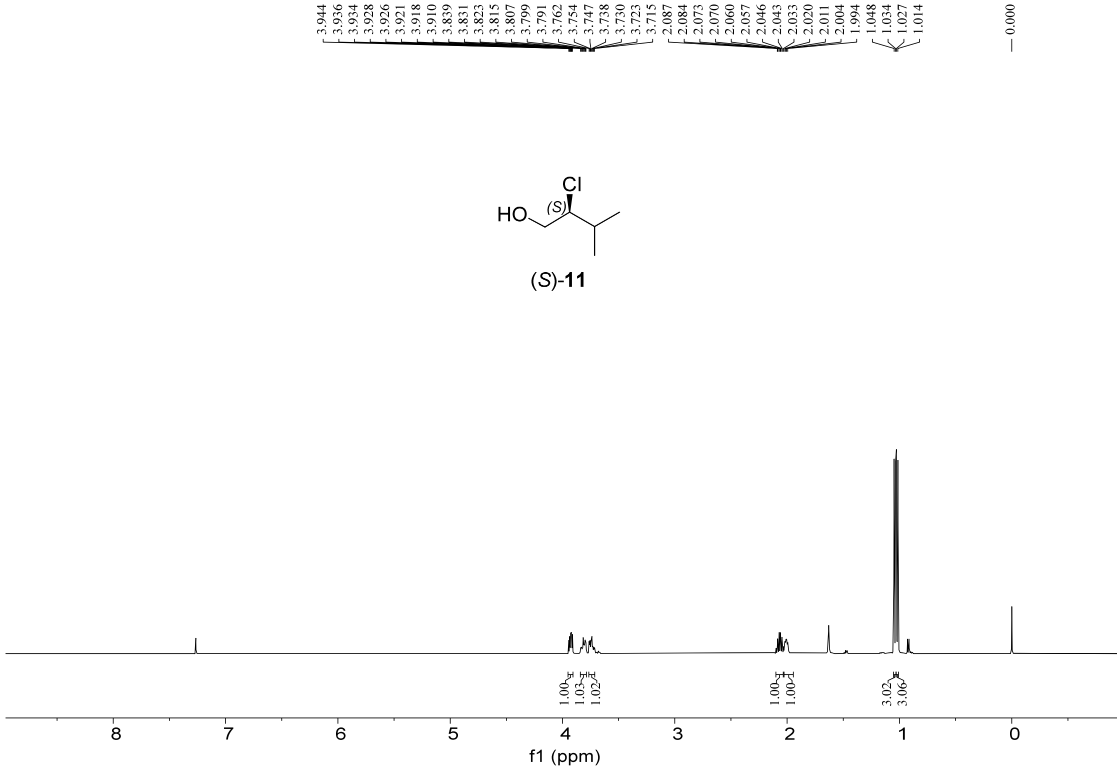

Figure S22. ^{13}C NMR Spectrum of (*S*)-2-chloro-3-methylbutan-1-ol ((*S*)-**11**) (126 MHz, $CDCl_3$)

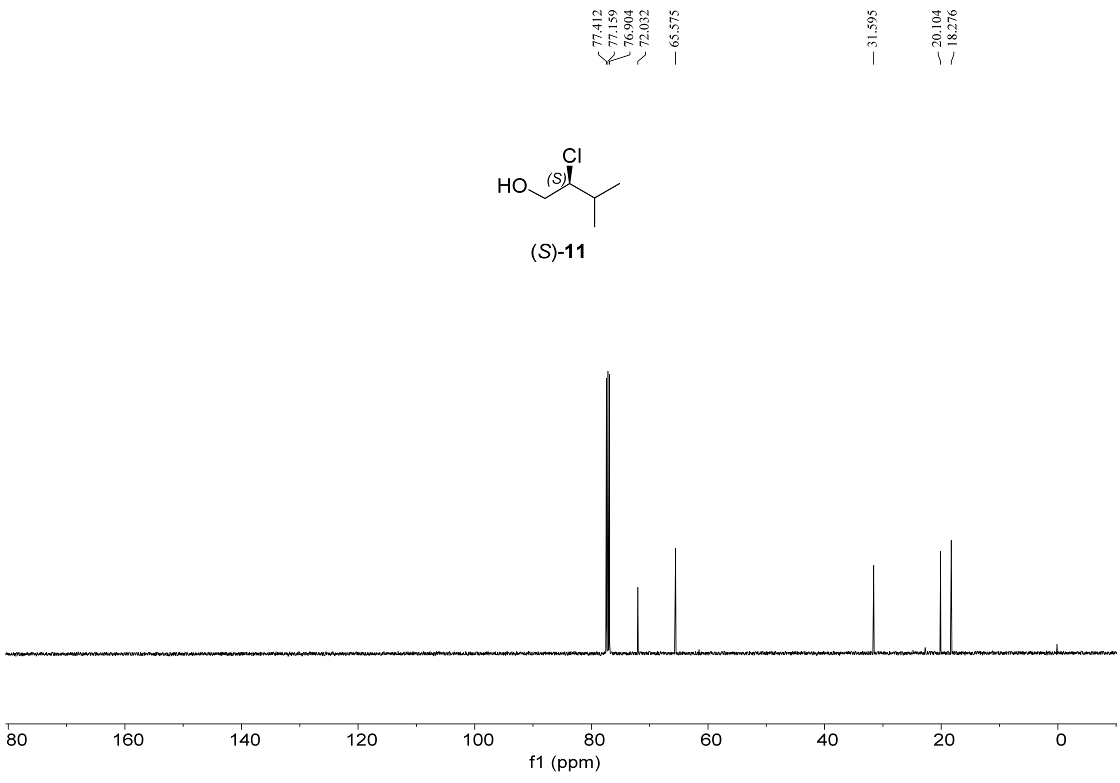

Figure S23. ¹H NMR Spectrum of (*R*)-2-isopropyloxirane ((*R*)-**12**) (500 MHz, $CDCl_3$)

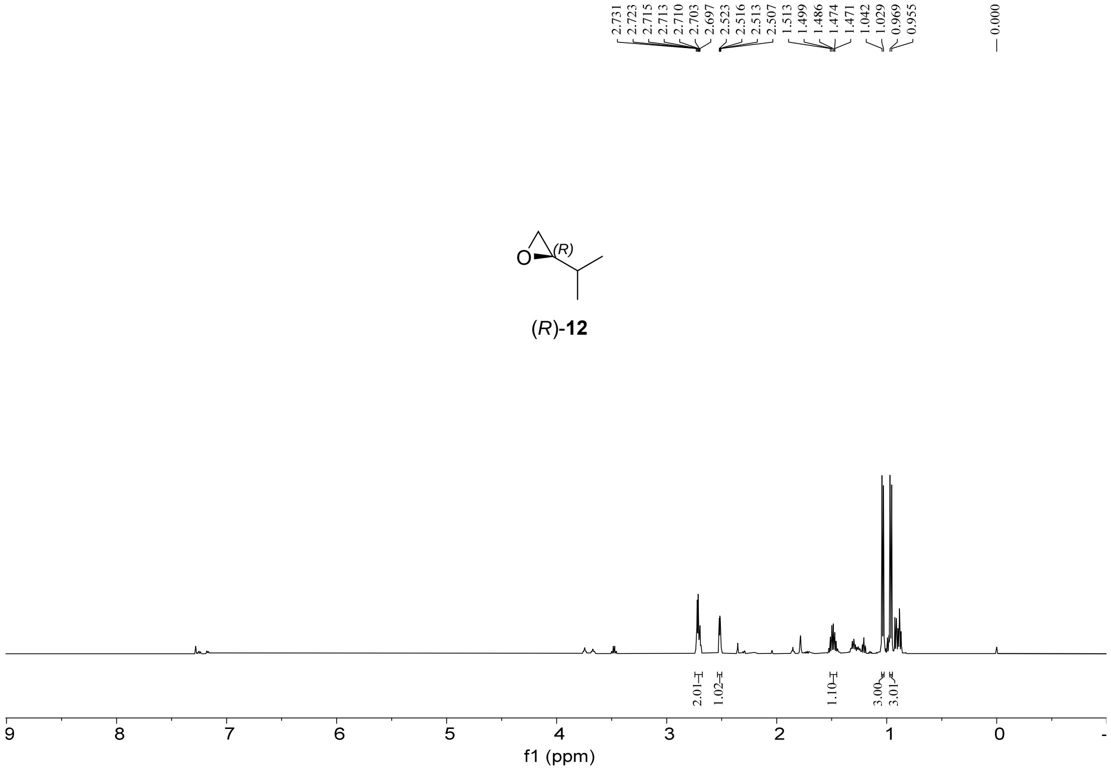

Figure S24. ¹³C NMR Spectrum of (*R*)-2-isopropyloxirane ((*R*)-**12**) (126 MHz, $CDCl_3$)

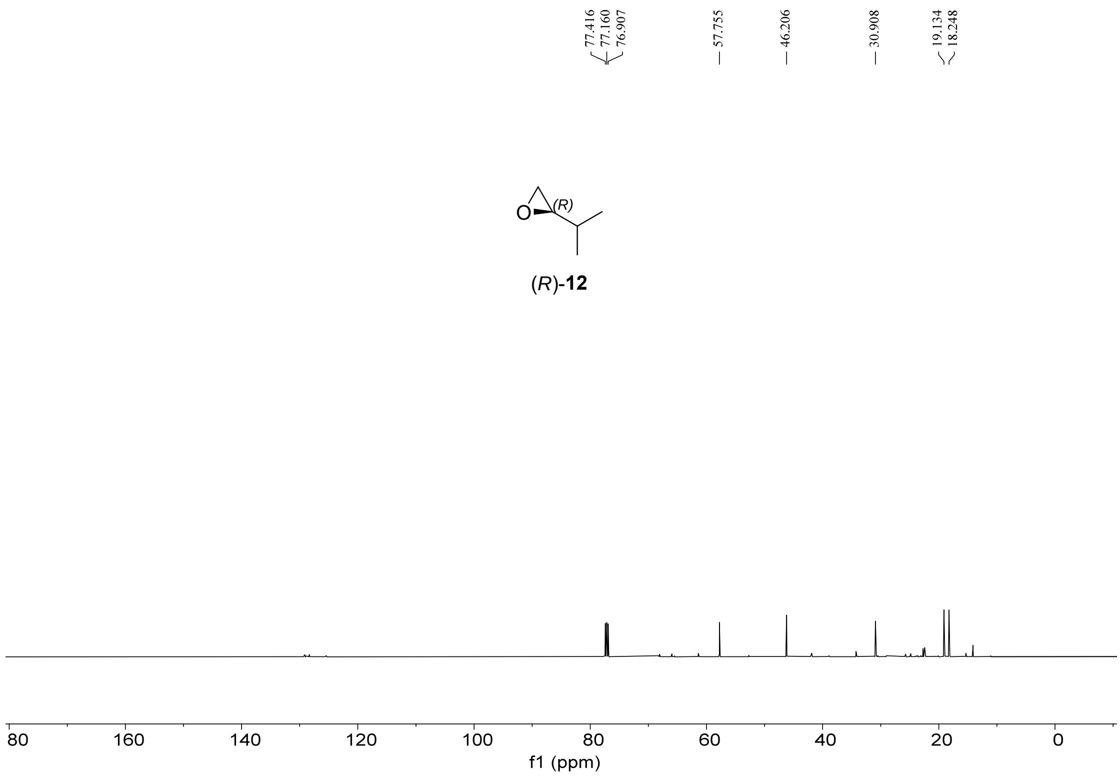

Figure S25. ^{1}H NMR Spectrum of (*S*)-2-methylpentan-3-ol ((*S*)-**13**) (500 MHz, $CDCl_3$)

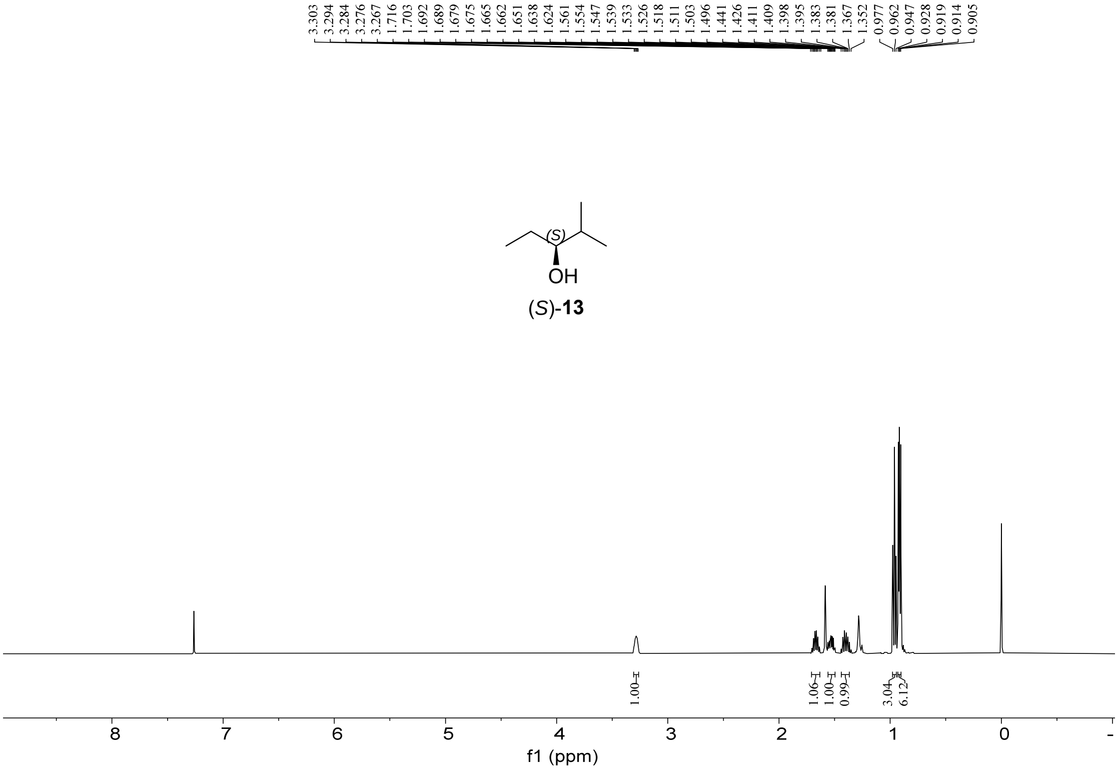

Figure S26. ^{13}C NMR Spectrum of (*S*)-2-methylpentan-3-ol ((*S*)-**13**) (126 MHz, $CDCl_3$)

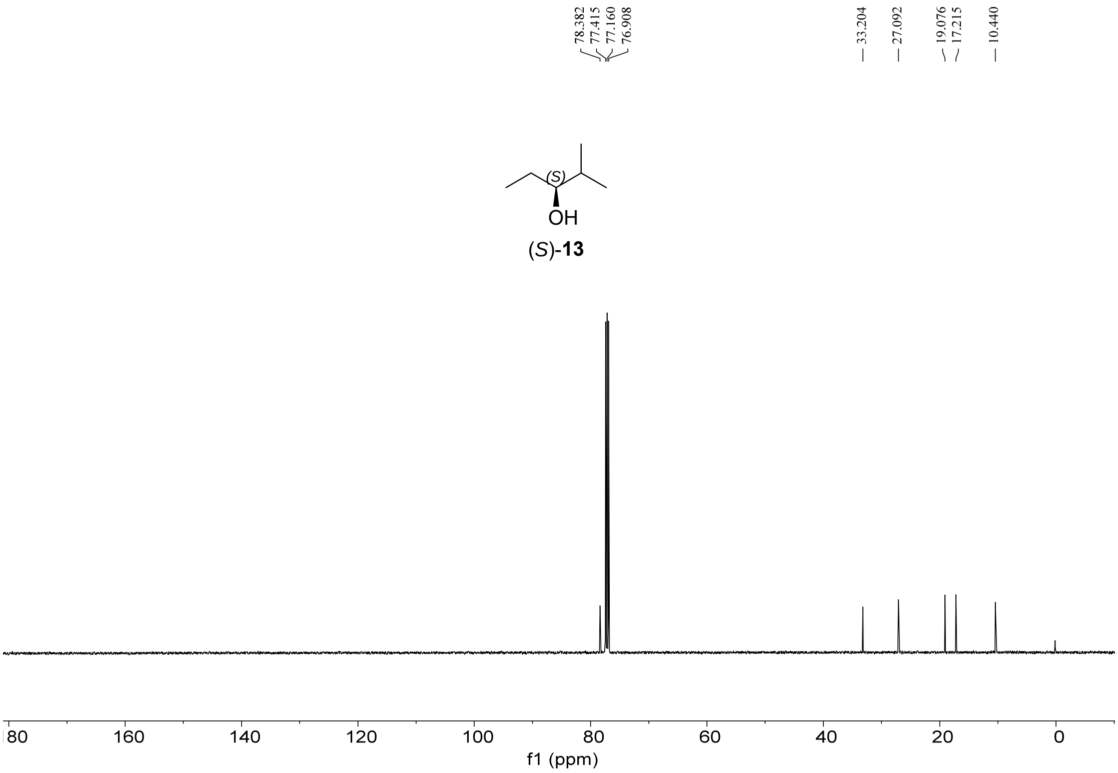

Figure S27. ^{1}H NMR Spectrum of (4*R*,14*R*)-4,14-dimethylhexadec-1-ene ((4*R*,14*R*)-**15**) (500 MHz, $CDCl_3$)

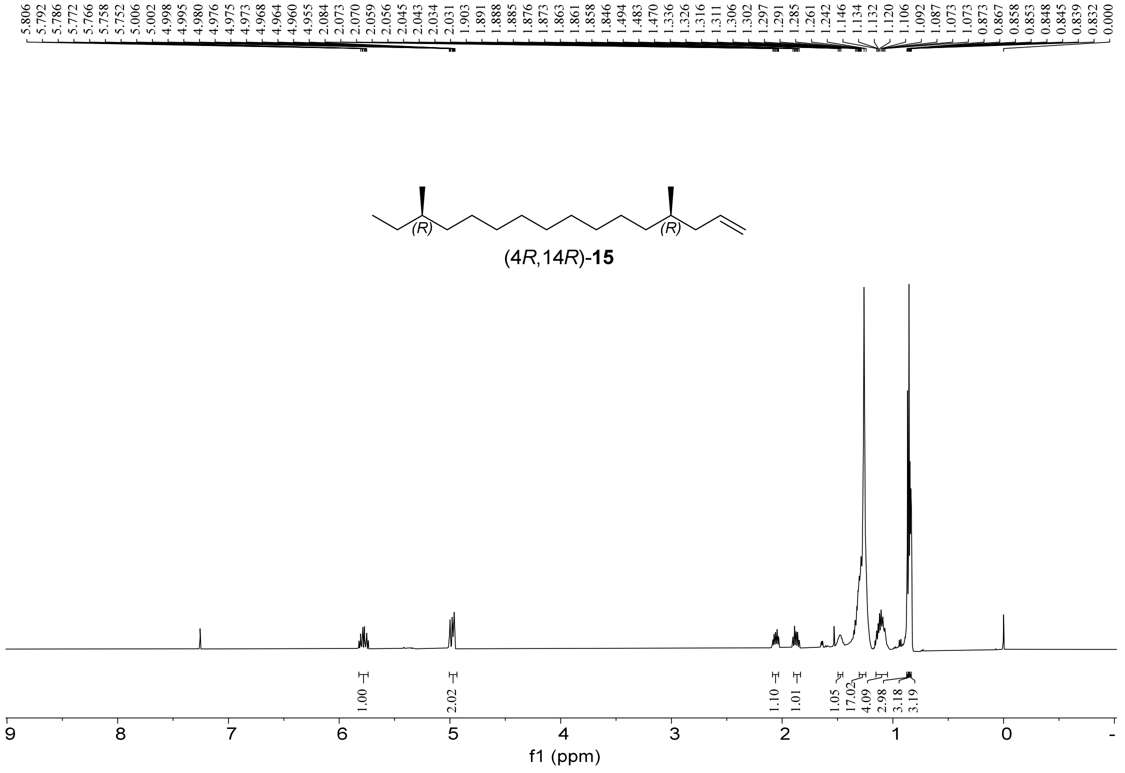

Figure S28. ^{13}C NMR Spectrum of (4*R*,14*R*)-4,14-dimethylhexadec-1-ene ((4*R*,14*R*)-**15**) (126 MHz, $CDCl_3$)

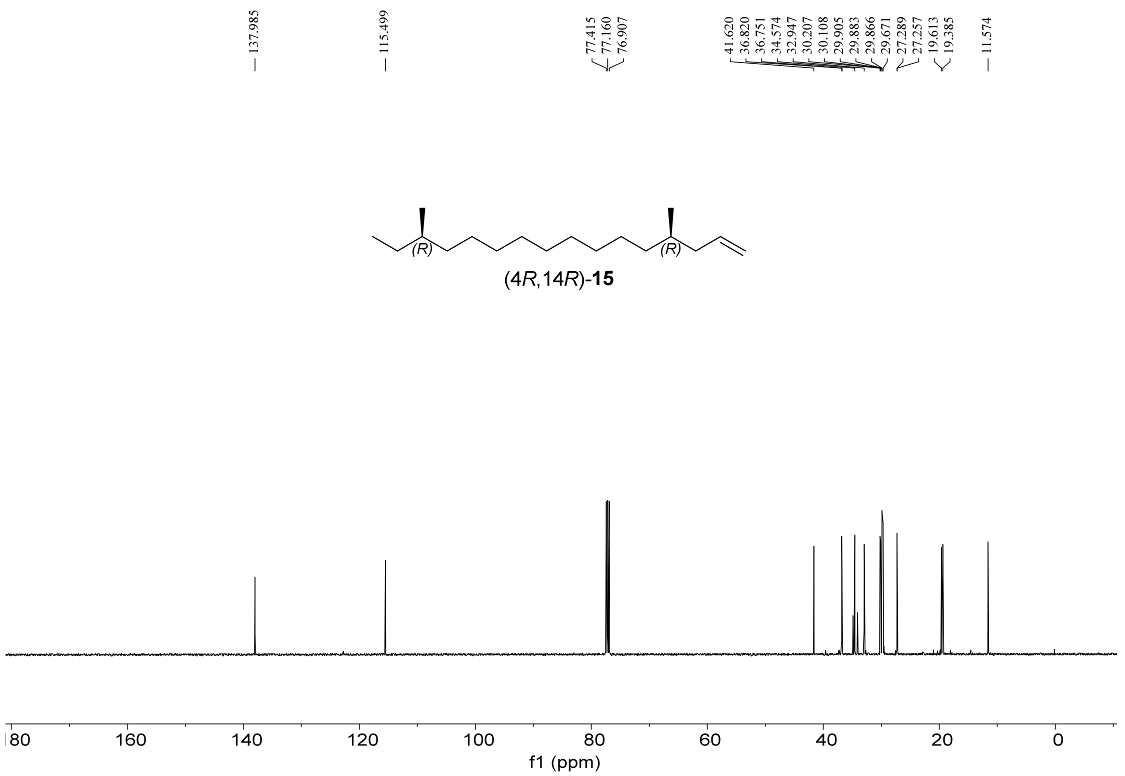

Figure S29. ^{1}H NMR Spectrum of (3*R*,13*R*)-3,13-dimethylpentadecanoic acid ((3*R*,13*R*)-**16**) (500 MHz, $CDCl_3$)

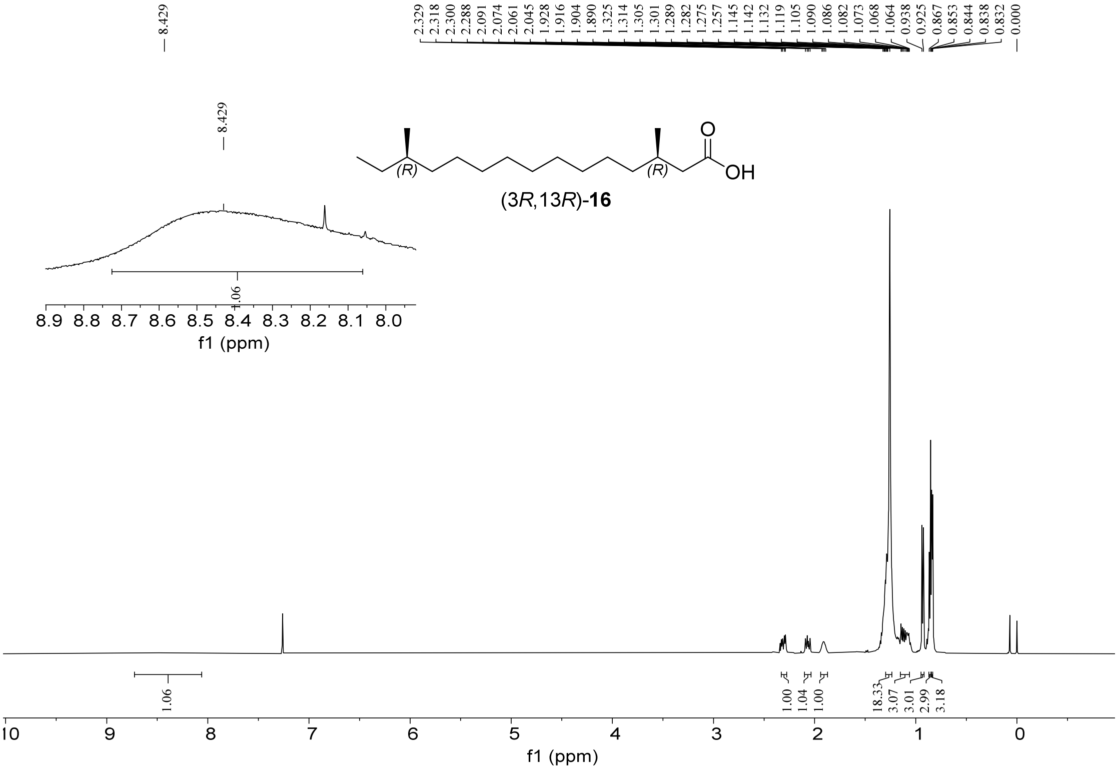

Figure S30. ^{13}C NMR Spectrum of (3*R*,13*R*)-3,13-dimethylpentadecanoic acid ((3*R*,13*R*)-**16**) (126 MHz, $CDCl_3$)

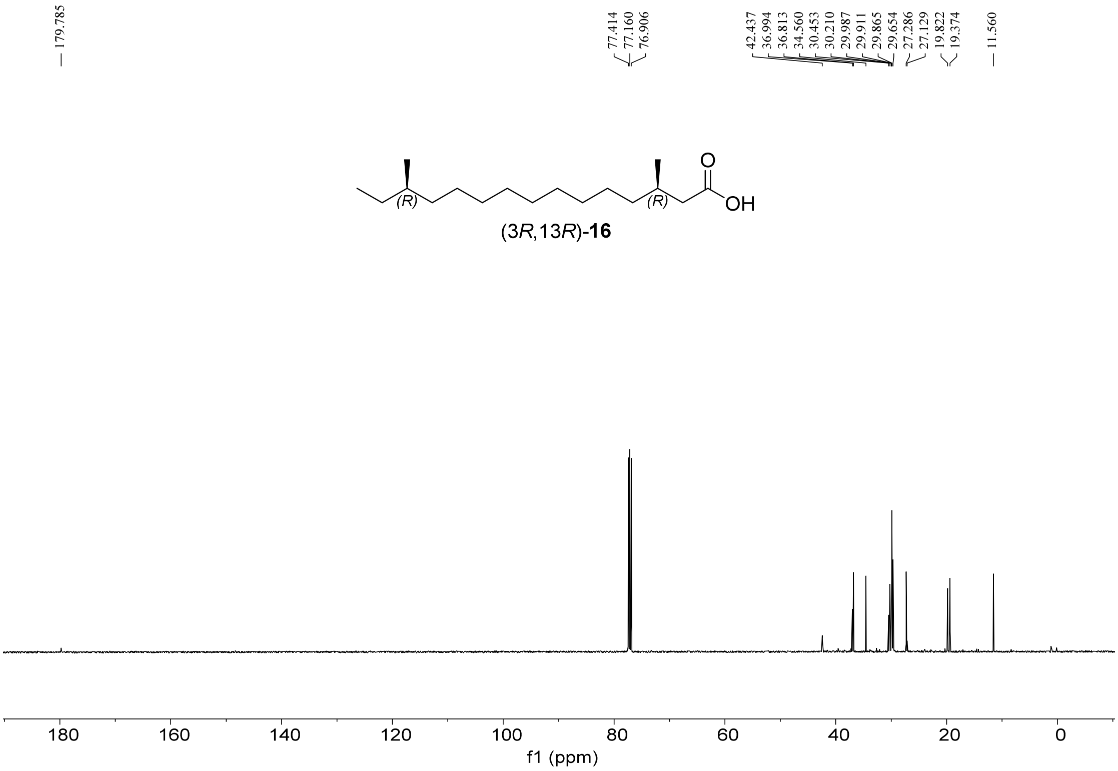

Figure S31. ^{1}H NMR Spectrum of (4*R*,14*S*)-4,14-dimethylhexadec-1-ene ((4*R*,14*S*)-**15**) (500 MHz, $CDCl_3$)

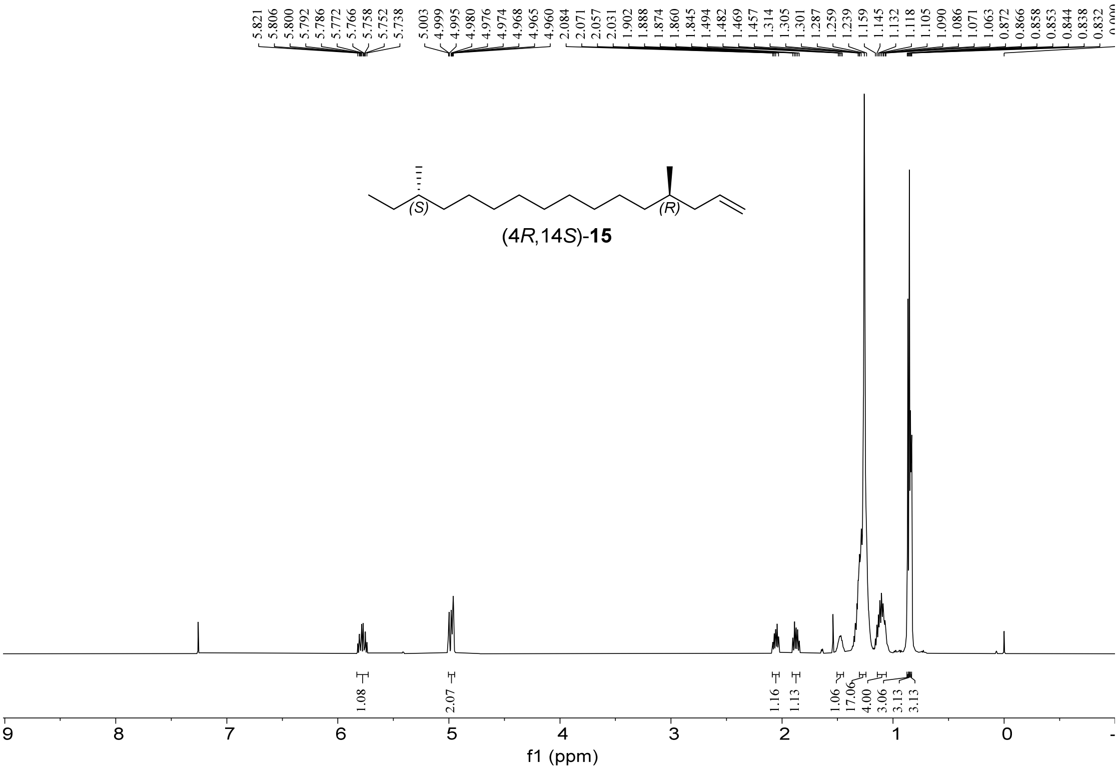

Figure S32. ^{13}C NMR Spectrum of (4*R*,14*S*)-4,14-dimethylhexadec-1-ene ((4*R*,14*S*)-**15**) (126 MHz, $CDCl_3$)

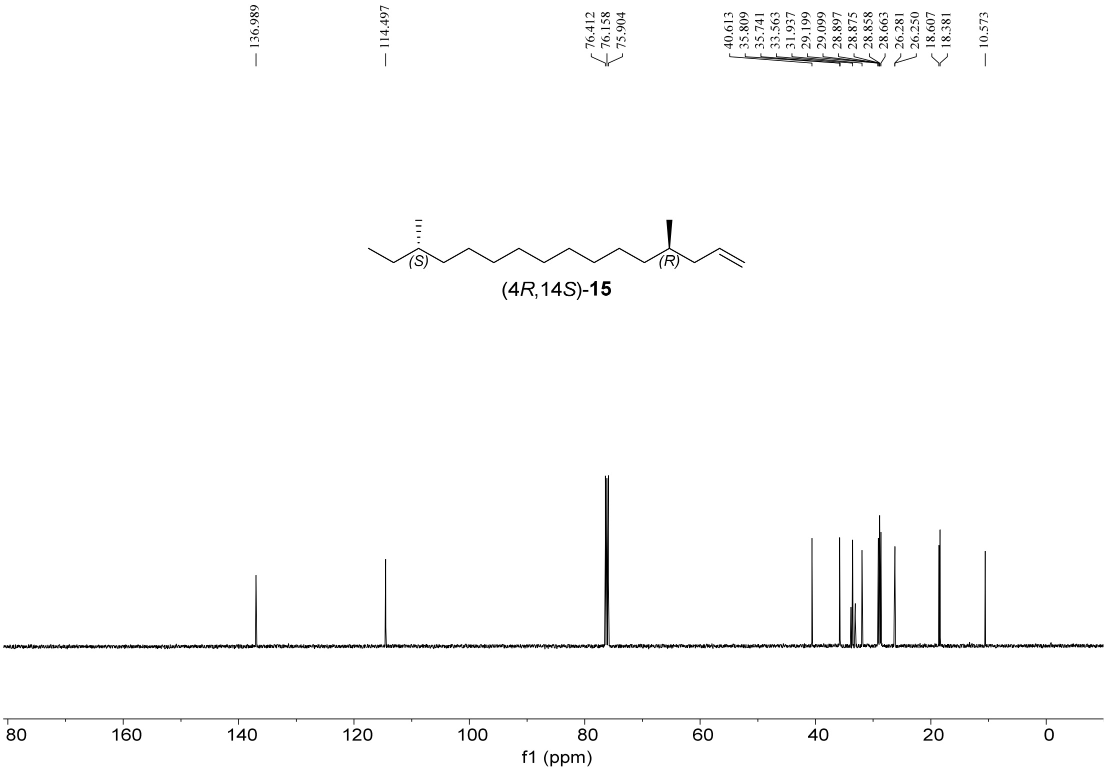

Figure S33. ^1H NMR Spectrum of (3*R*,13*S*)-3,13-dimethylpentadecanoic acid ((3*R*,13*S*)-**16**) (500 MHz, $CDCl_3$)

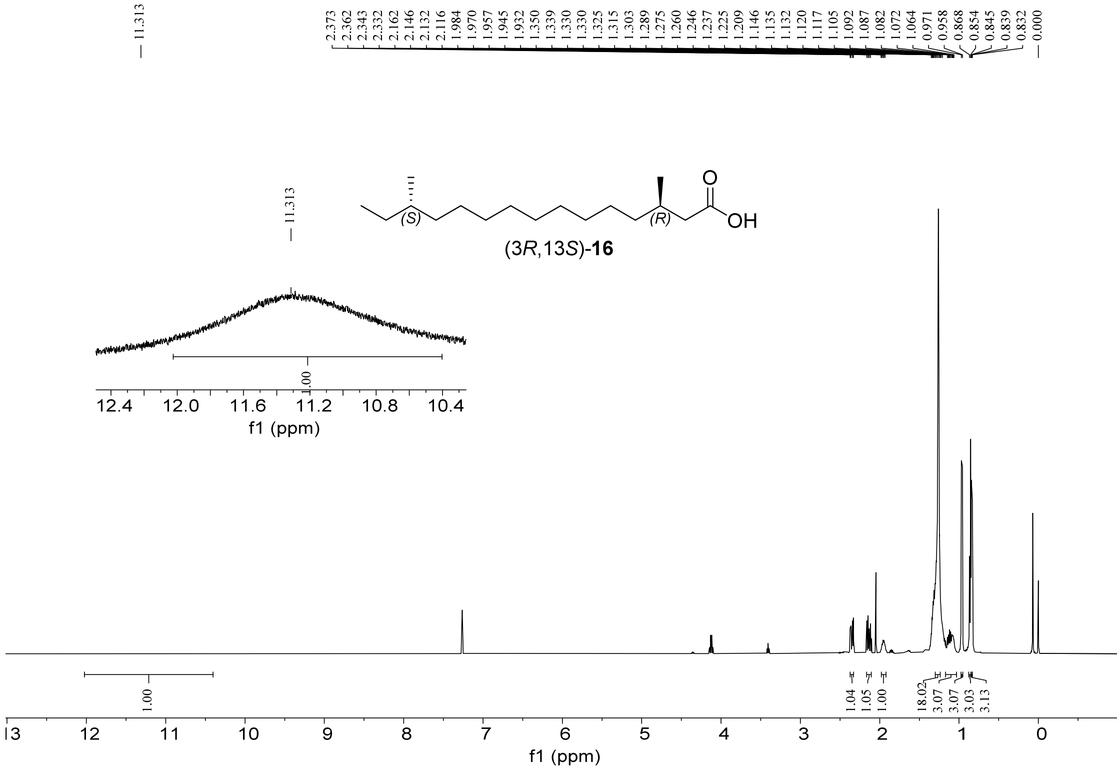

Figure S34. ^{13}C NMR Spectrum of (3*R*,13*S*)-3,13-dimethylpentadecanoic acid ((3*R*,13*S*)-**16**) (126 MHz, $CDCl_3$)

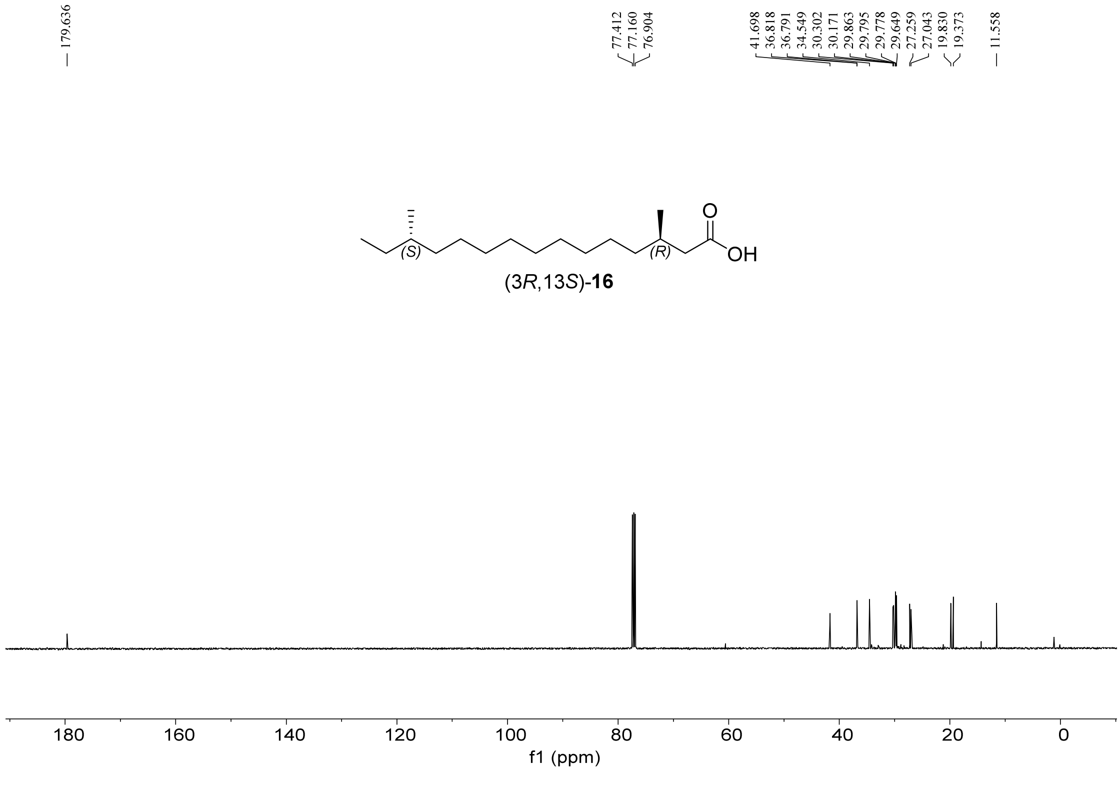

Figure S35. ^{1}H NMR Spectrum of (*S*)-2-methylpentan-3-yl (3*R*,13*R*)-3,13-dimethylpentadecanoate (**1a**) (500 MHz, $CDCl_3$)

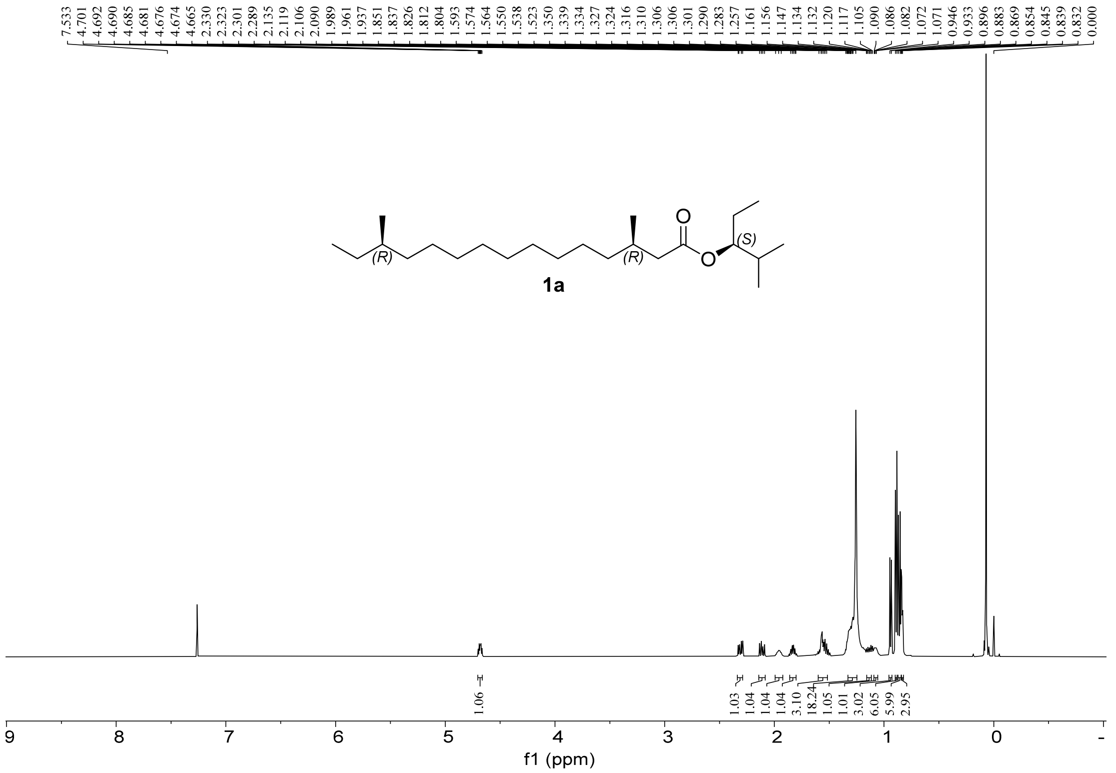

Figure S36. ^{13}C NMR Spectrum of (*S*)-2-methylpentan-3-yl (3*R*,13*R*)-3,13-dimethylpentadecanoate (**1a**) (126 MHz, $CDCl_3$)

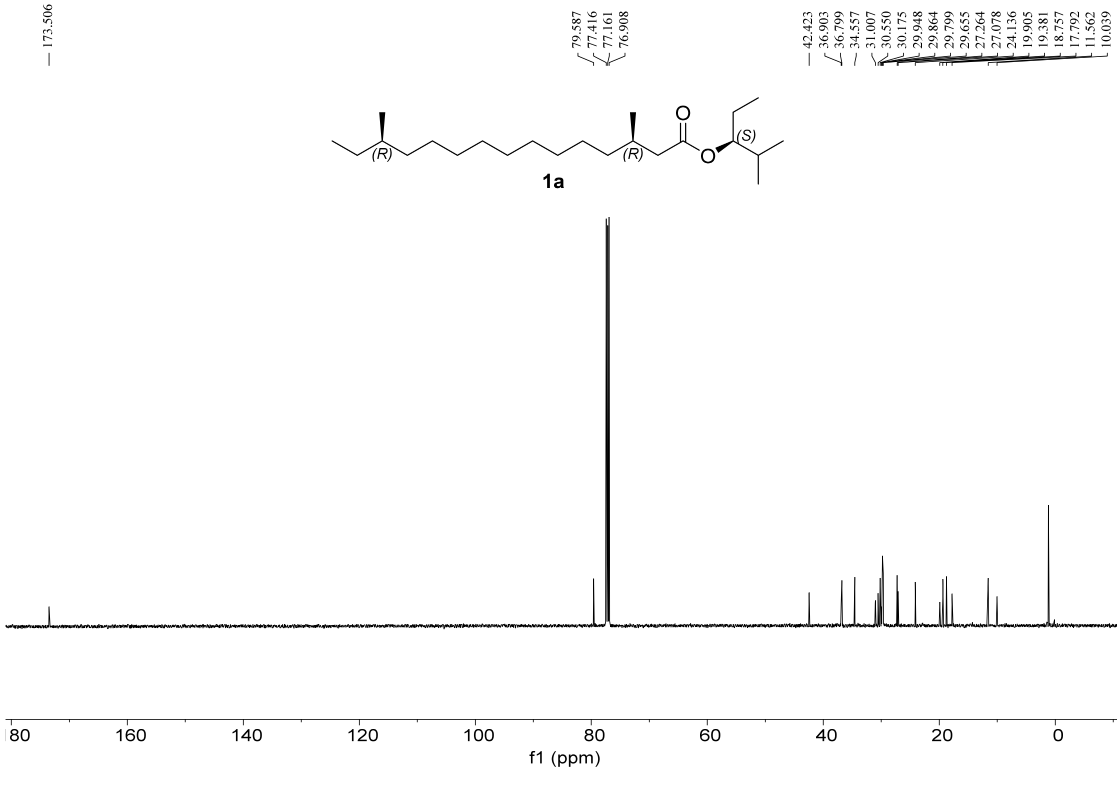

Figure S37. ^{1}H NMR Spectrum of (*S*)-2-methylpentan-3-yl (3*R*,13*S*)-3,13-dimethylpentadecanoate (**1b**) (500 MHz, $CDCl_3$)

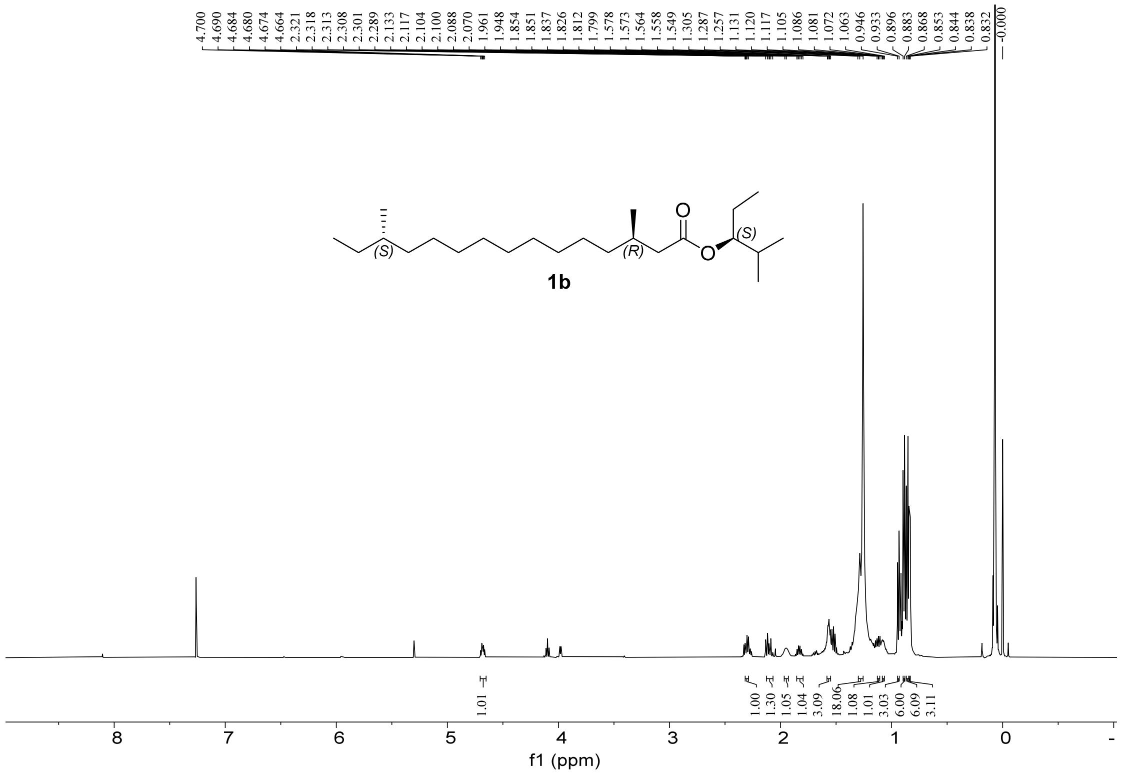

Figure S38. ^{13}C NMR Spectrum of (*S*)-2-methylpentan-3-yl (3*R*,13*S*)-3,13-dimethylpentadecanoate (**1b**) (126 MHz, $CDCl_3$)

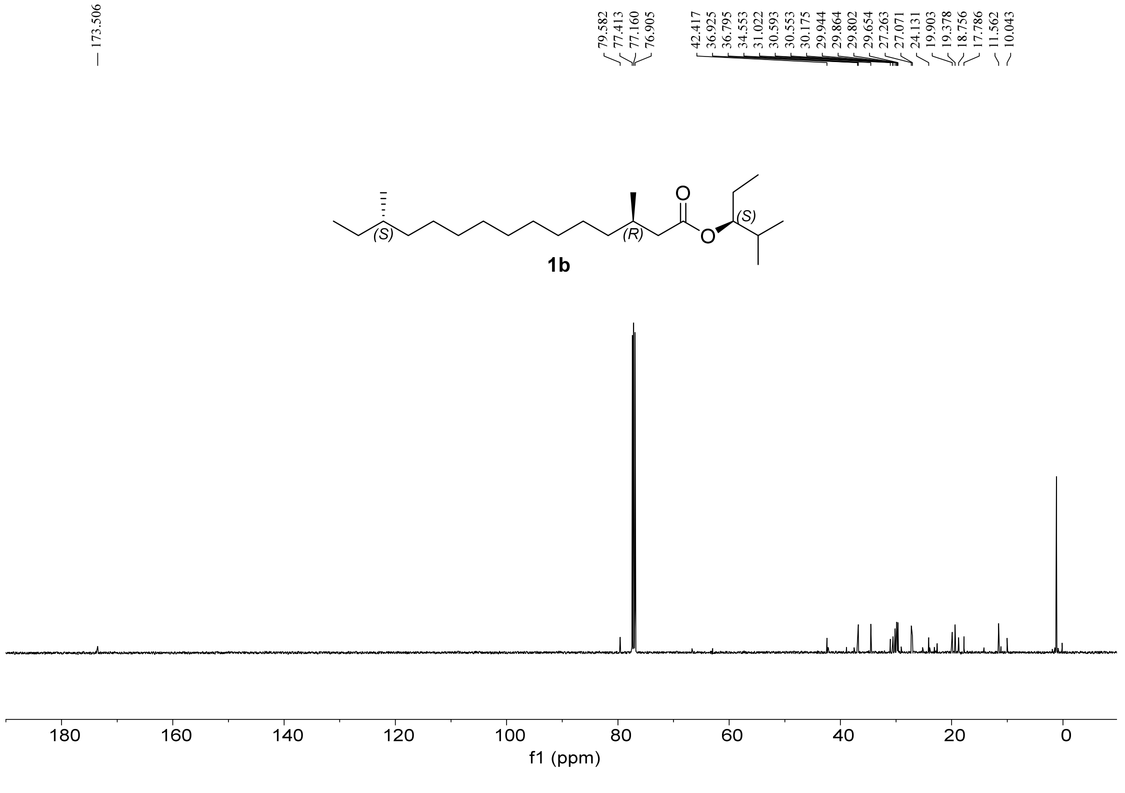

Figure S39. ^{1}H NMR Spectrum of (*R*)-2-methylpentan-3-yl (3*R*,13*R*)-3,13-dimethylpentadecanoate (**1c**) (500 MHz, $CDCl_3$)

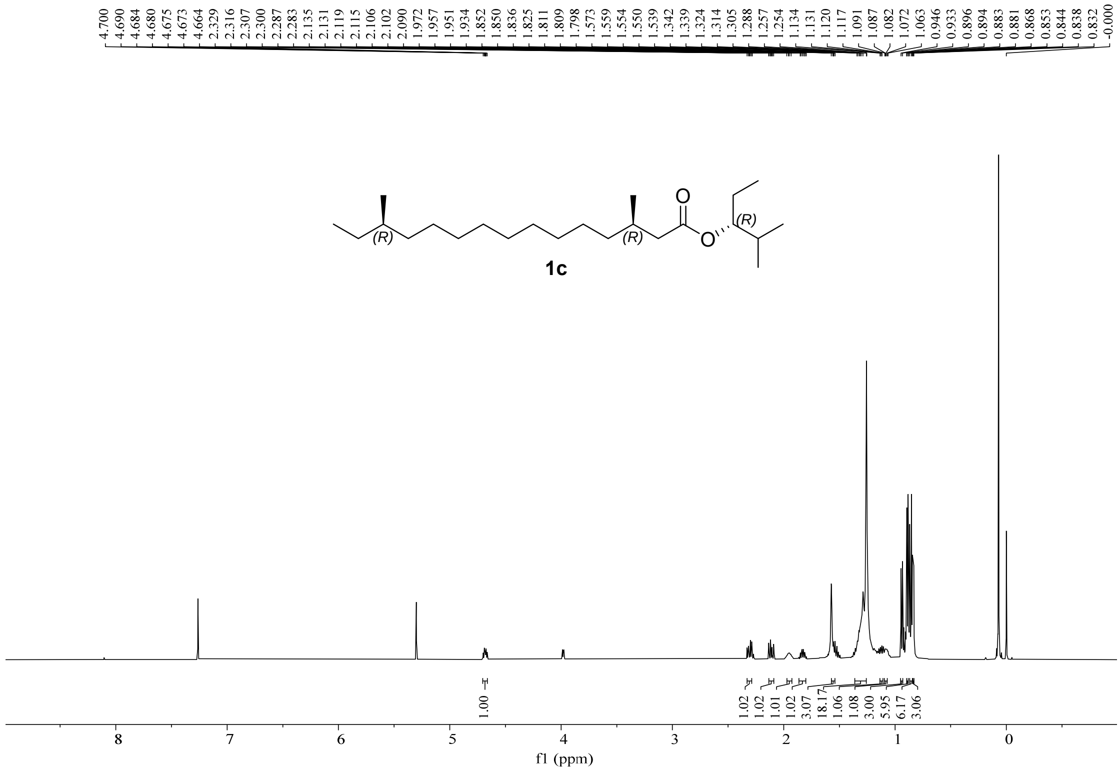

Figure S40. ^{13}C NMR Spectrum of (*R*)-2-methylpentan-3-yl (3*R*,13*R*)-3,13-dimethylpentadecanoate (**1c**) (126 MHz, $CDCl_3$)

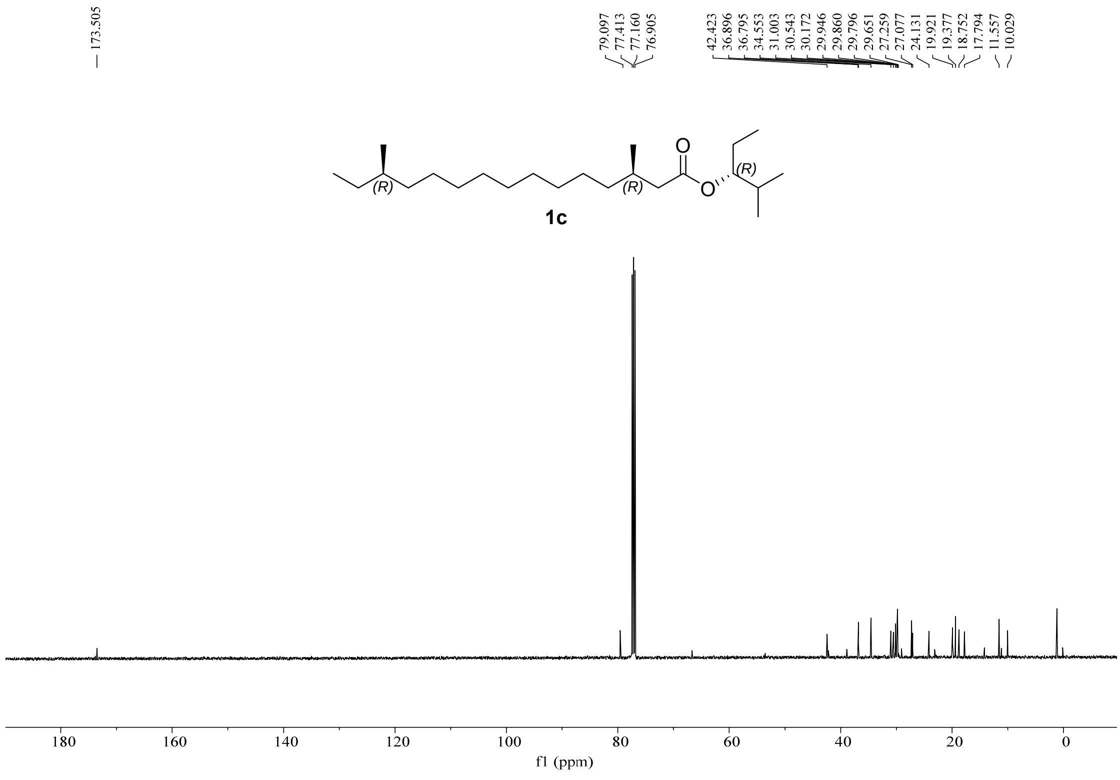

Figure S41. ^{1}H NMR Spectrum of (*R*)-2-methylpentan-3-yl (3*R*,13*S*)-3,13-dimethylpentadecanoate (**1d**) (500 MH z, $CDCl_3$)

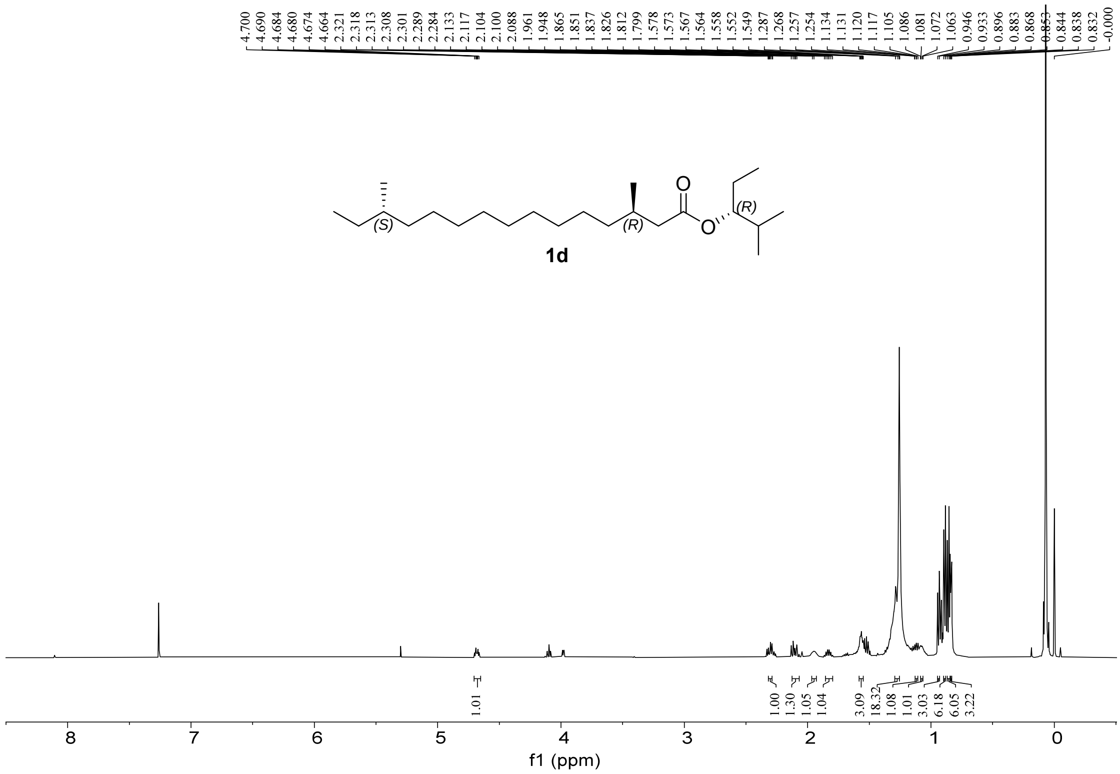

Figure S42. ^{13}C NMR Spectrum of (*R*)-2-methylpentan-3-yl (3*R*,13*S*)-3,13-dimethylpentadecanoate (**1d**) (126 MHz, $CDCl_3$)

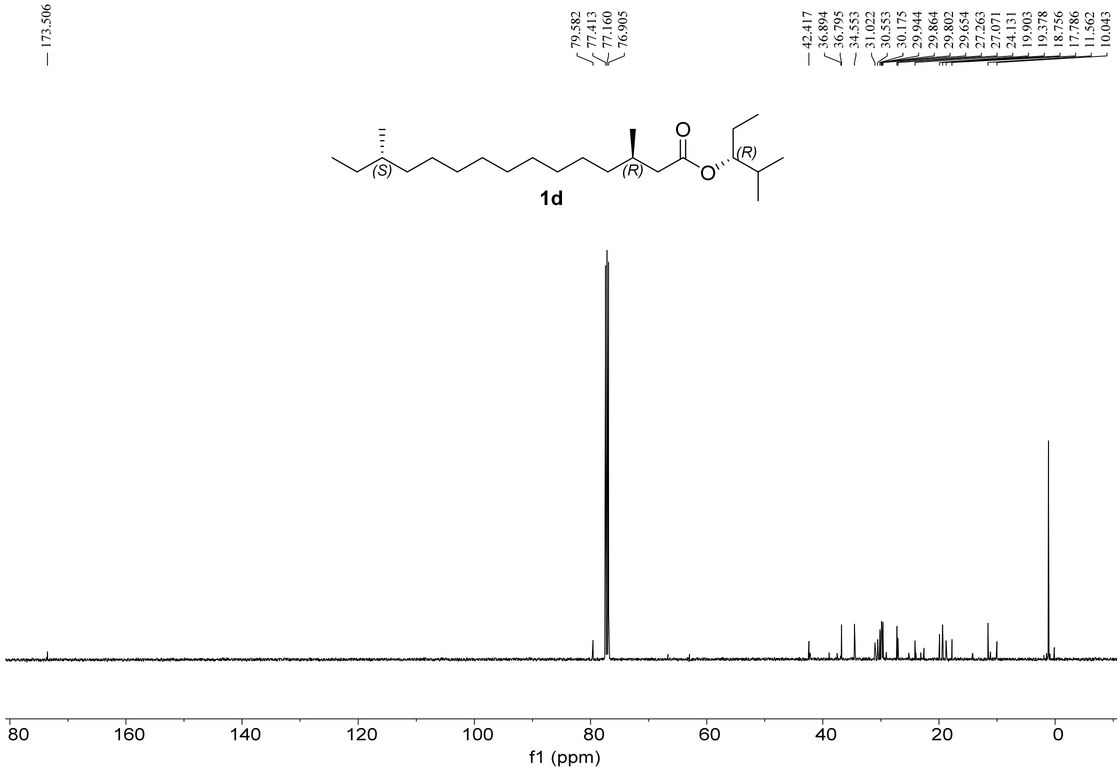

Figure S43. ^{1}H NMR Spectrum of 10-((tetrahydro-2*H*-pyran-2-yl)oxy)decan-2-yl (*S*)-3,3,3-trifluoro-2-methoxy-2-phenylpropanoate ((*S*)-**18**) (500 MHz, $CDCl_3$)

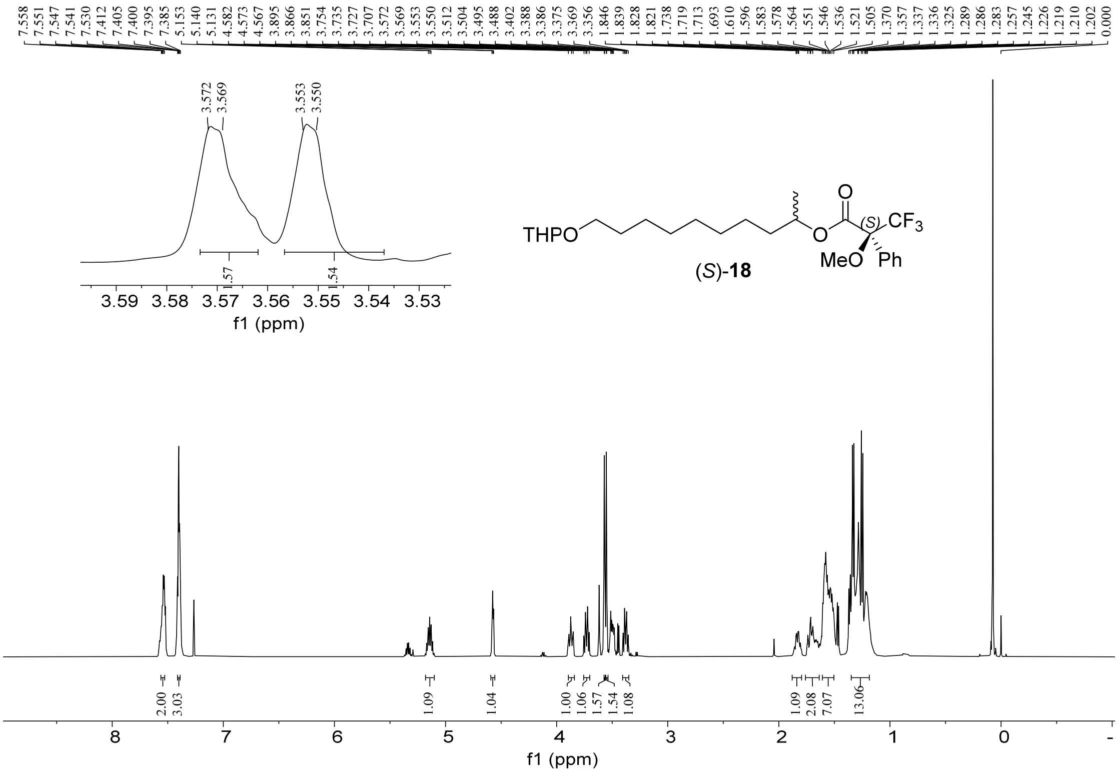

Figure S44. ^{13}C NMR Spectrum of 10-((tetrahydro-2*H*-pyran-2-yl)oxy)decan-2-yl (2*S*)-3,3,3-trifluoro-2-methoxy-2-phenylpropanoate ((*S*)-**18**) (126 MHz, $CDCl_3$)

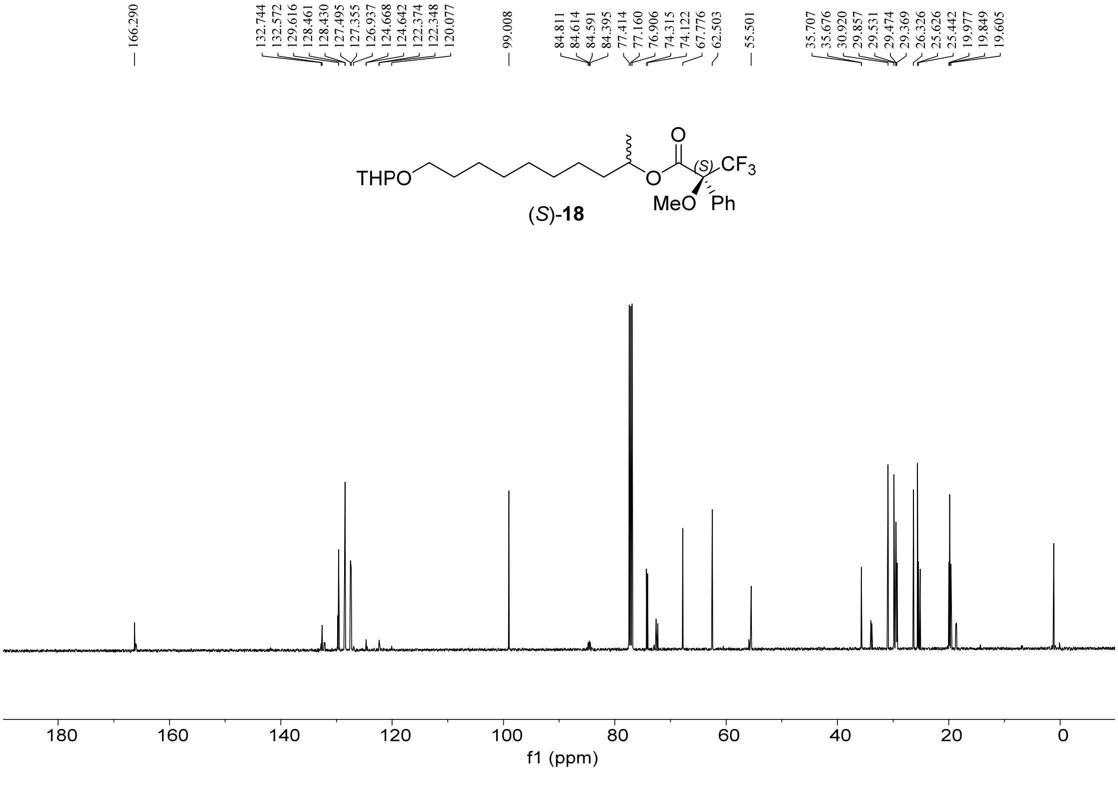

Figure S45. ^{19}F NMR Spectrum of 10-((tetrahydro-2*H*-pyran-2-yl)oxy)decan-2-yl (2*S*)-3,3,3-trifluoro-2-methoxy-2-phenylpropanoate ((*S*)-**18**) (471 M, $CDCl_3$)

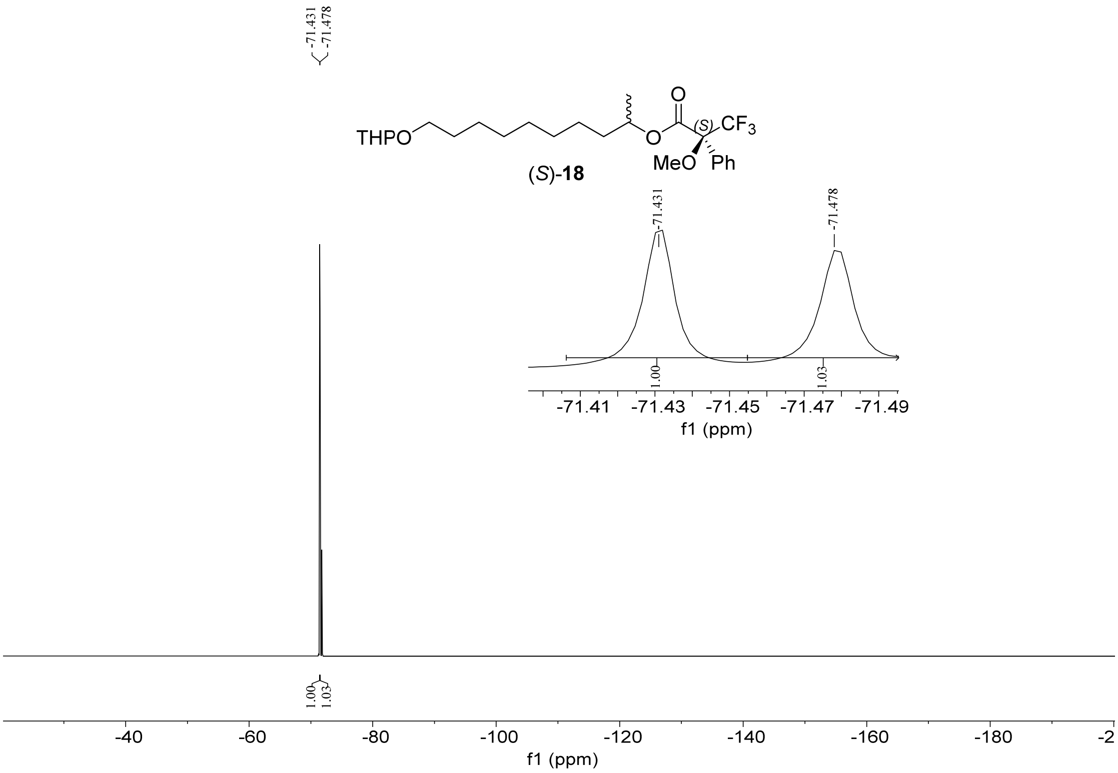

Figure S46. ^{1}H NMR Spectrum of (2*S*)-10-((tetrahydro-2*H*-pyran-2-yl)oxy)decan-2-yl (2*S*)-3,3,3-trifluoro-2-methoxy-2-phenylpropanoate ((*S*,*S*)-**18**) (500 MHz, $CDCl_3$)

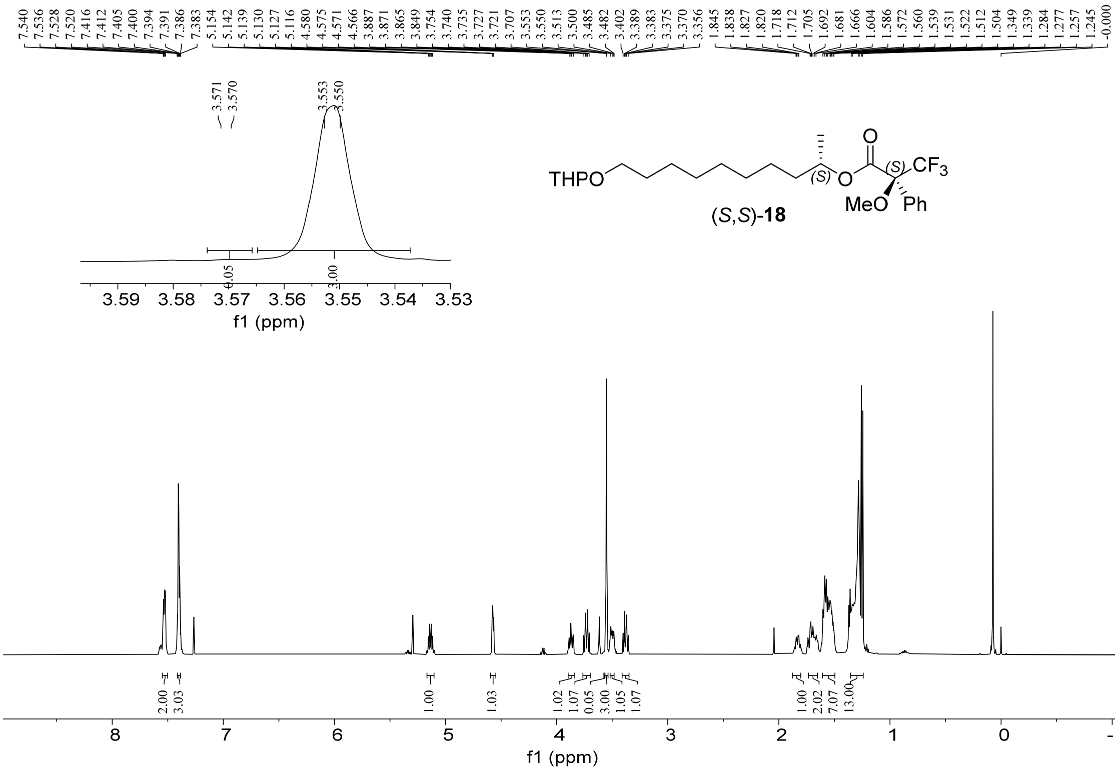

Figure S47. ^{13}C NMR Spectrum of (2*S*)-10-((tetrahydro-2*H*-pyran-2-yl)oxy)decan-2-yl (2*S*)-3,3,3-trifluoro-2-methoxy-2-phenylpropanoate ((*S*,*S*)-**18**) (126 MHz, CDCl3)

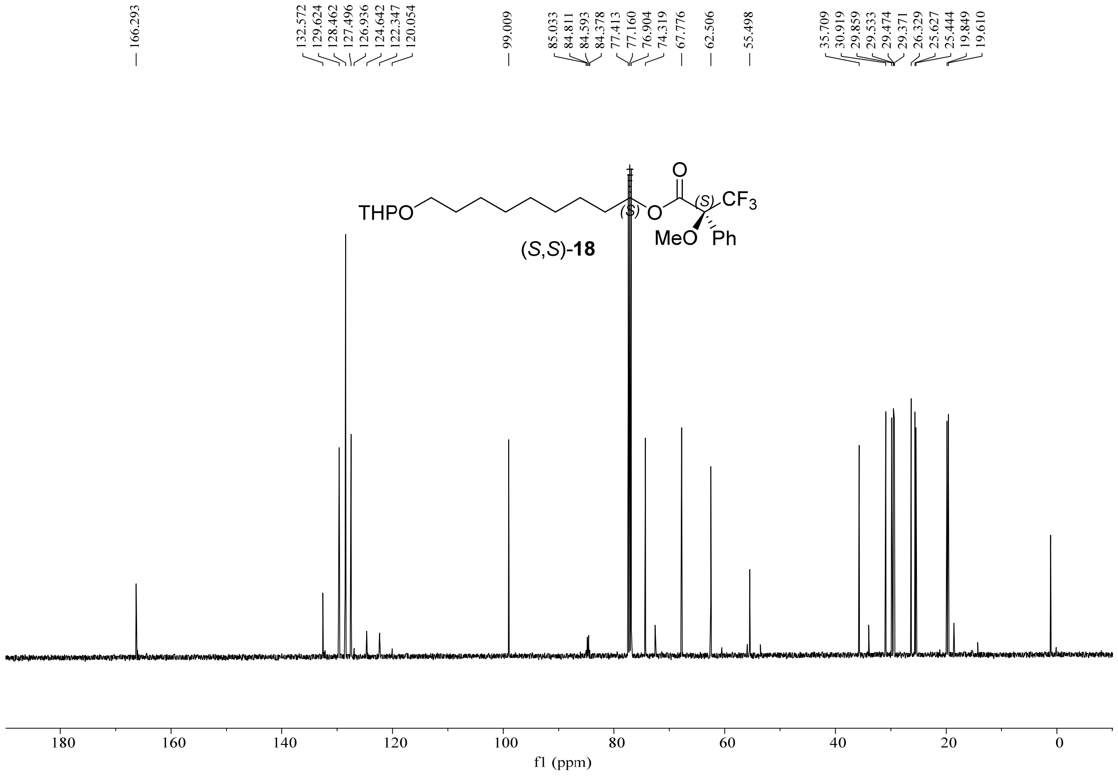

Figure S48. ^{19}F NMR Spectrum of (2*S*)-10-((tetrahydro-2*H*-pyran-2-yl)oxy)decan-2-yl (2*S*)-3,3,3-trifluoro-2-methoxy-2-phenylpropanoate ((*S*,*S*)-**18**) (471 M, CDCl3)

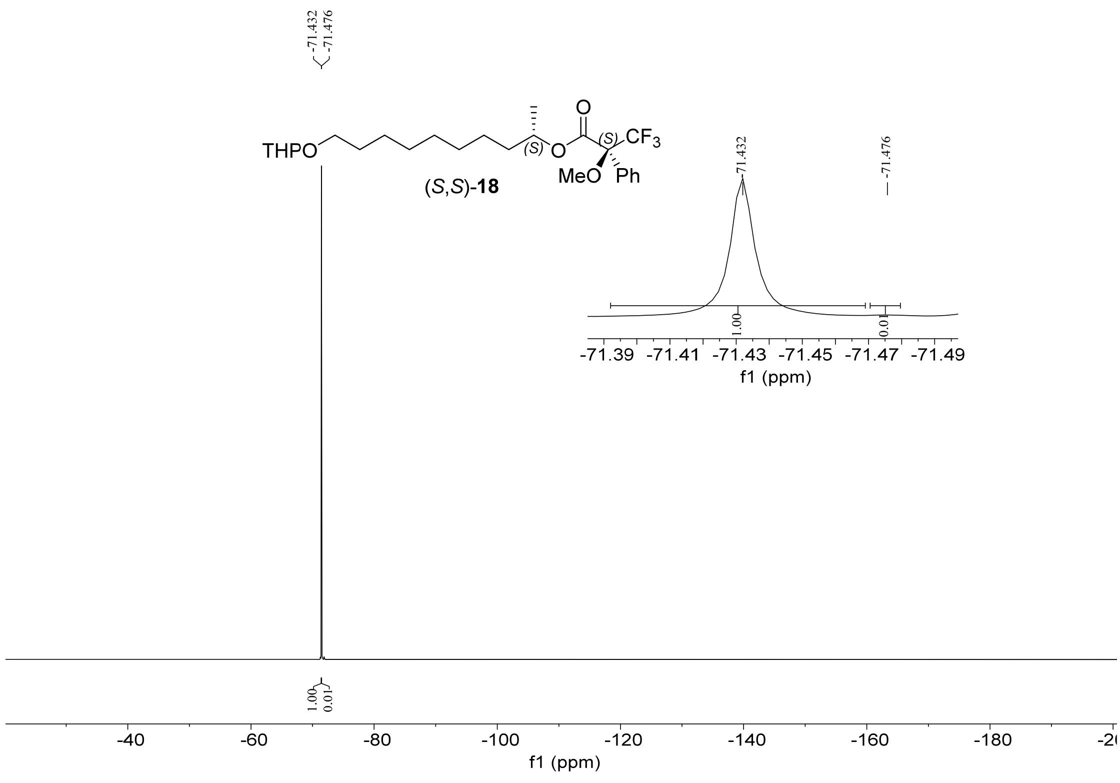

Figure S49. ^{1}H NMR Spectrum of 2-methylpentan-3-yl 3,5-dinitrobenzoate (*rac*-**20**) (500 MHz, $CDCl_3$)

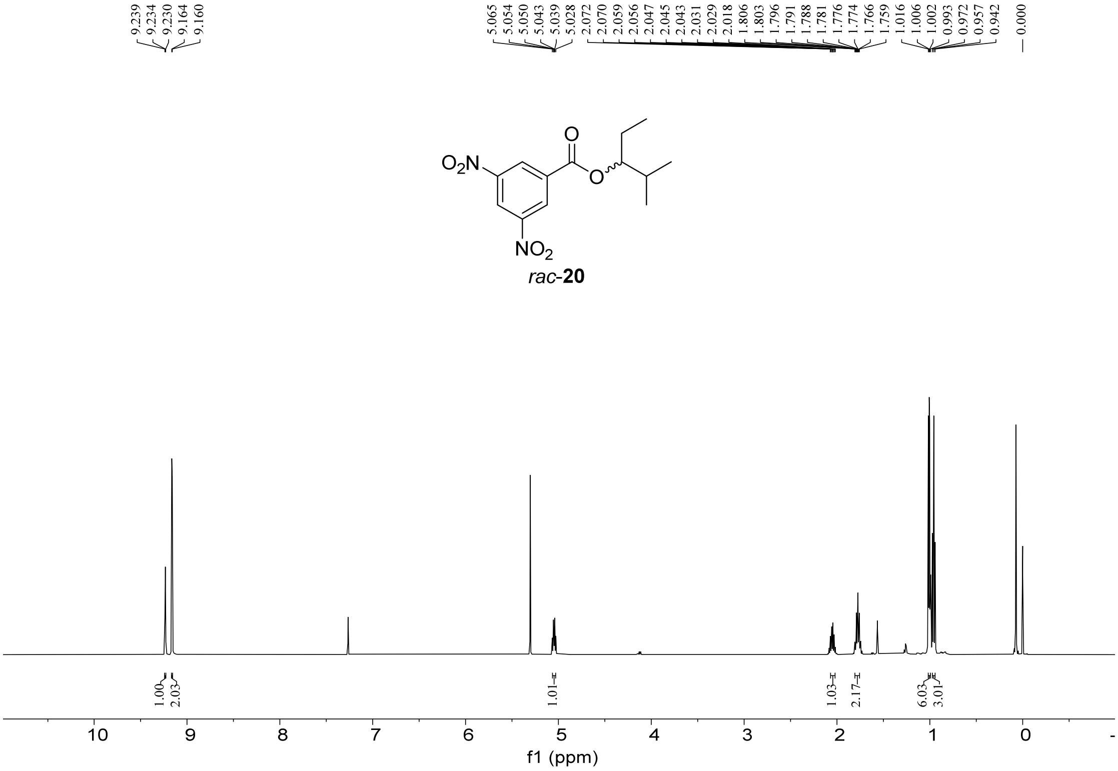

Figure S50. ^{13}C NMR Spectrum of 2-methylpentan-3-yl 3,5-dinitrobenzoate (*rac*-**20**) (126 MHz, $CDCl_3$)

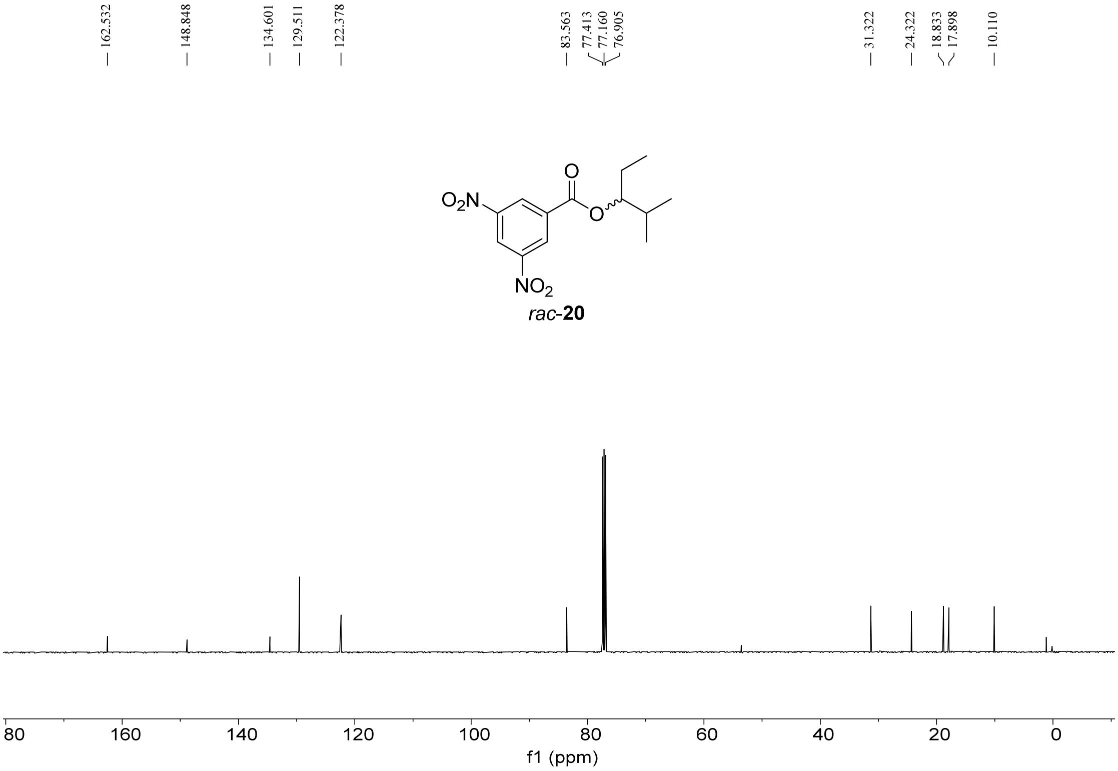

Figure S51. ^{1}H NMR Spectrum of (*S*)-2-methylpentan-3-yl 3,5-dinitrobenzoate ((*S*)-**20**) (500 MHz, $CDCl_3$)

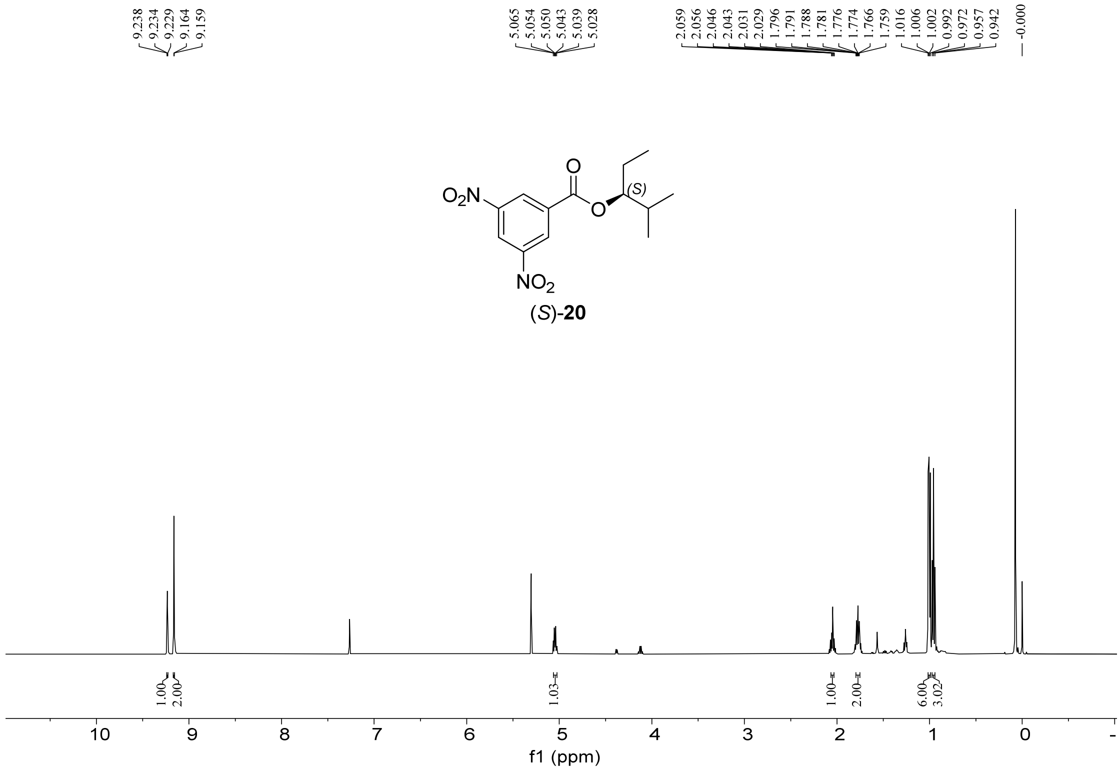

Figure S52. ^{13}C NMR Spectrum of (*S*)-2-methylpentan-3-yl 3,5-dinitrobenzoate ((*S*)-**20**) (126 MHz, $CDCl_3$)

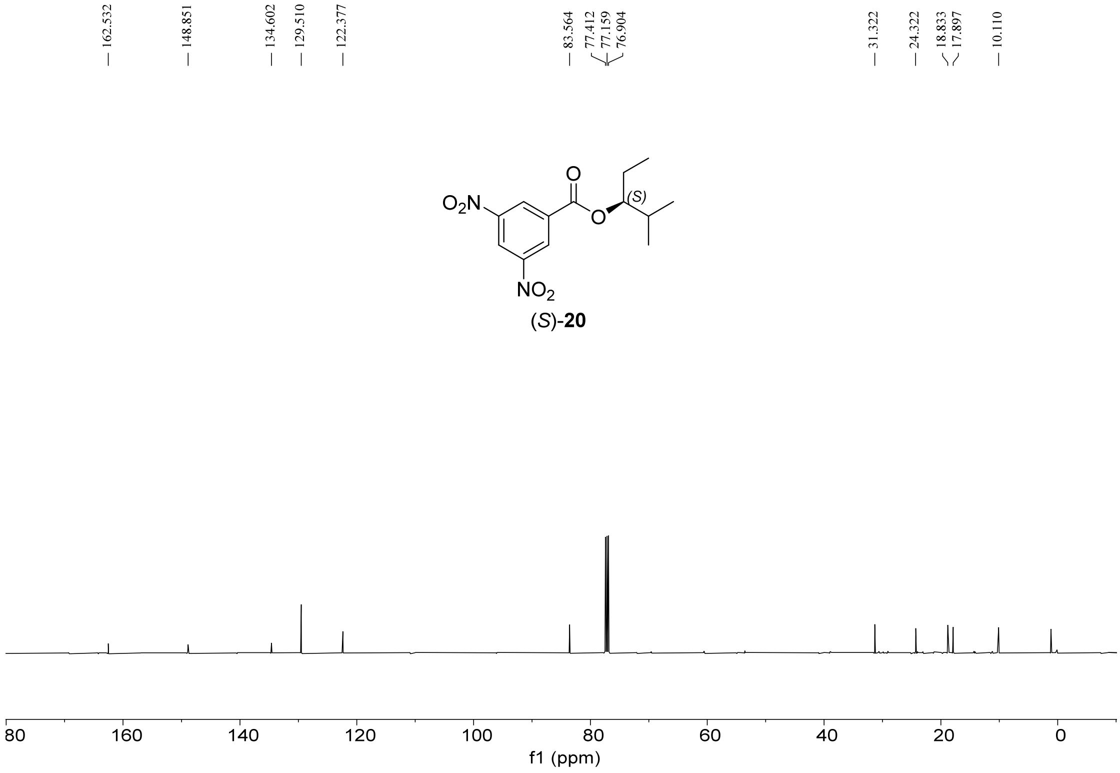

Figure S53. ^{1}H NMR Spectrum of (*R*)-2-methylpentan-3-yl 3,5-dinitrobenzoate ((*R*)-**20**) (500 MHz, $CDCl_3$)

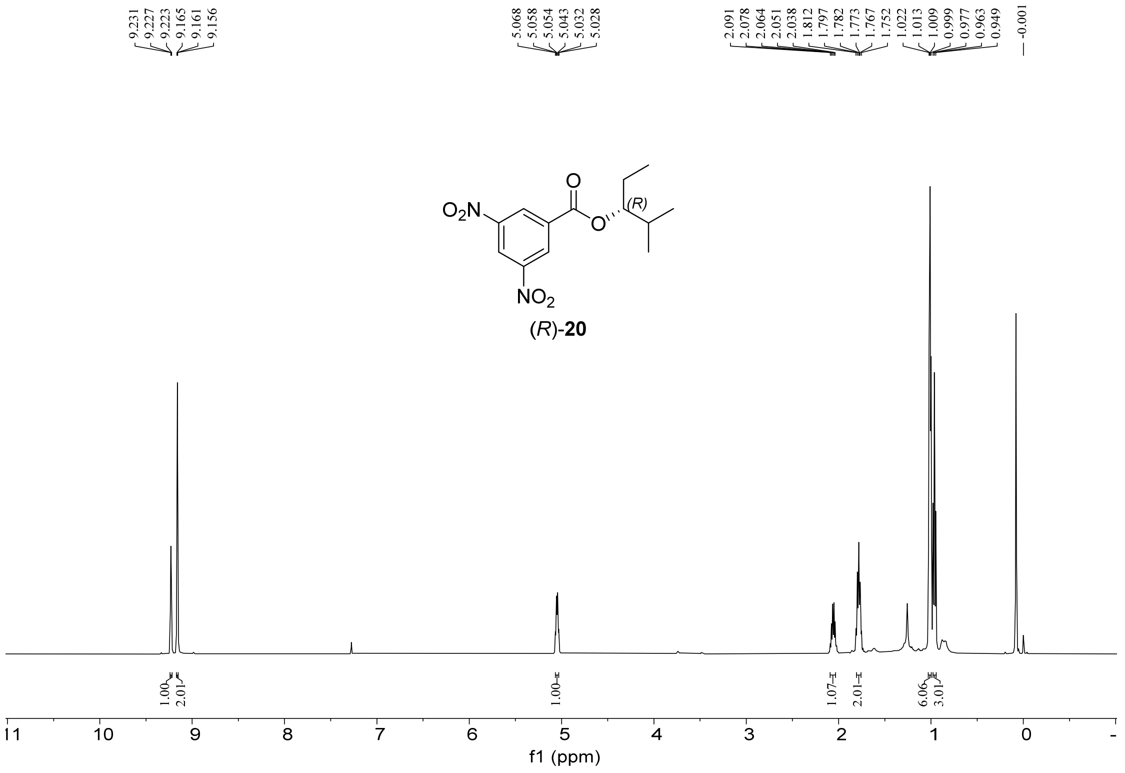

Figure S54. ^{13}C NMR Spectrum of (*R*)-2-methylpentan-3-yl 3,5-dinitrobenzoate ((*R*)-**20**) (126 MHz, $CDCl_3$)

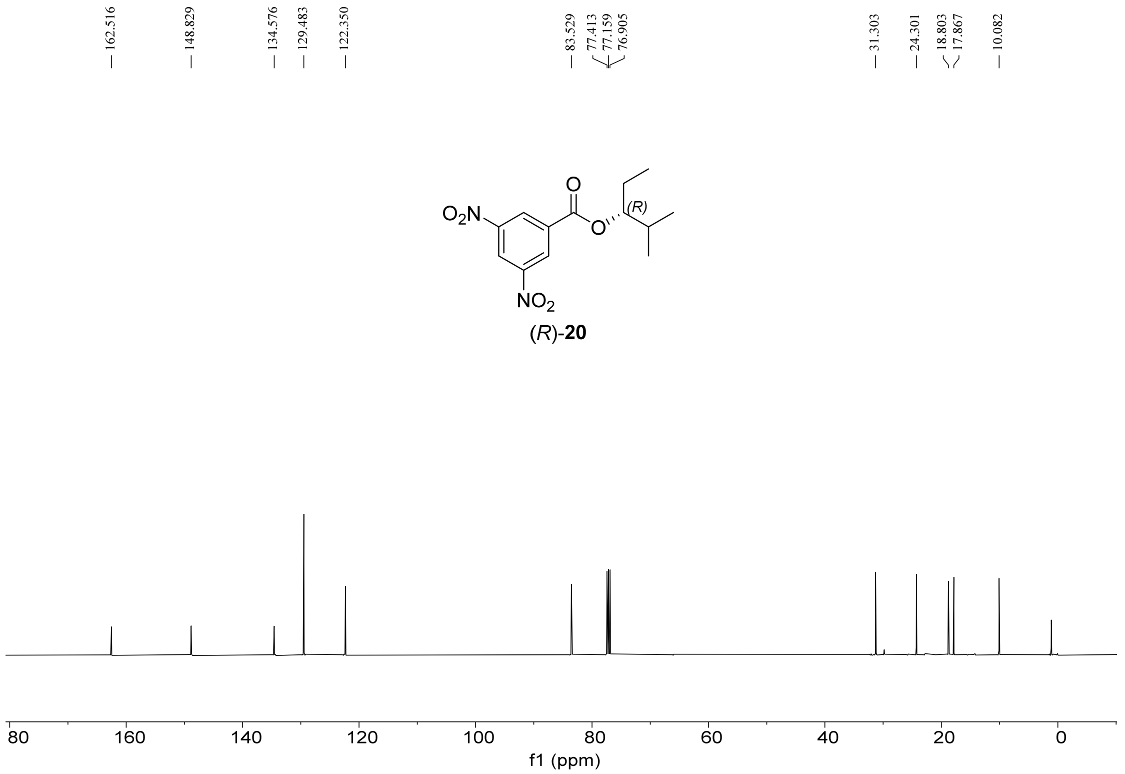

6. HPLC Chromatography of the Compounds

Figure S55. HPLC Chromatography of 2-methylpentan-3-yl 3,5-dinitrobenzoate ((*rac*)-**20**) (Daicel Chiralcel OJ-H column; *n*-hexane/2-propanol = 99.5:0.5, 1.0 mL/min, 254 nm)

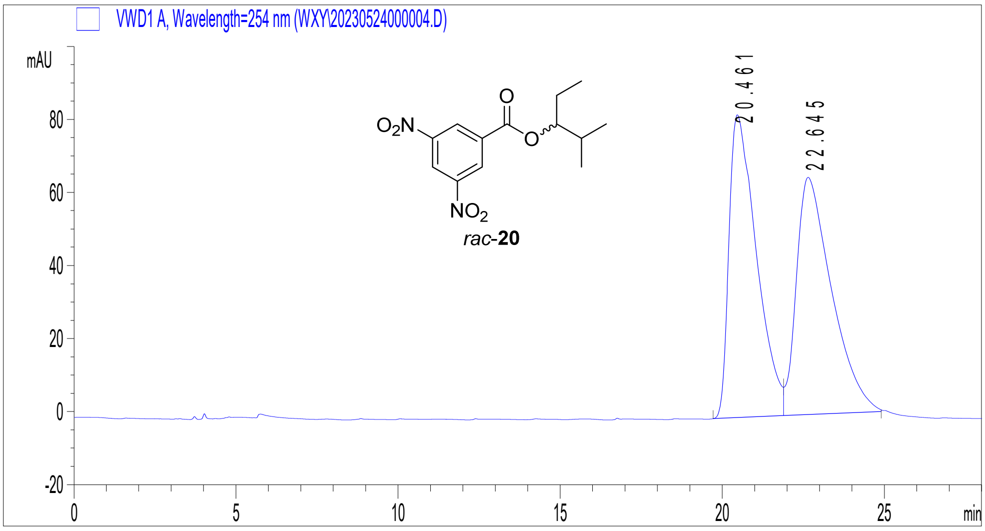

Peak #	RetTime [min]	Type	Width [min]	Area mAU*s	Height [mAU]	Area %
1	20.461	BV	0.8338	4895.17725	82.97945	49.8479
2	22.645	VB	1.1148	4925.05273	65.05032	50.1521

Figure S56. HPLC Chromatography of (*S*)-2-methylpentan-3-yl 3,5-dinitrobenzoate ((*S*)-**20**) (Daicel Chiralcel OJ-H column; *n*-hexane/2-propanol = 99.5:0.5, 1.0 mL/min, 254 nm)

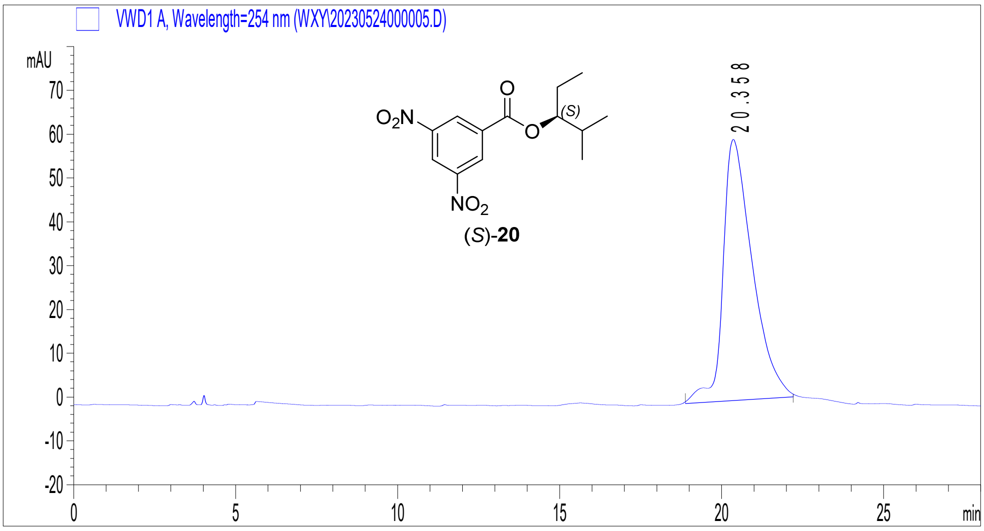

Peak #	RetTime [min]	Type	Width [min]	Area mAU*s	Height [mAU]	Area %
1	20.358	BB	0.9261	3767.01904	59.64439	100.0000

Figure S57. HPLC Chromatography of (*R*)-2-methylpentan-3-yl 3,5-dinitrobenzoate ((*R*)-**20**) (Daicel Chiralcel OJ-H column; *n*-hexane/2-propanol = 99.5:0.5, 1.0 mL/min, 254 nm)

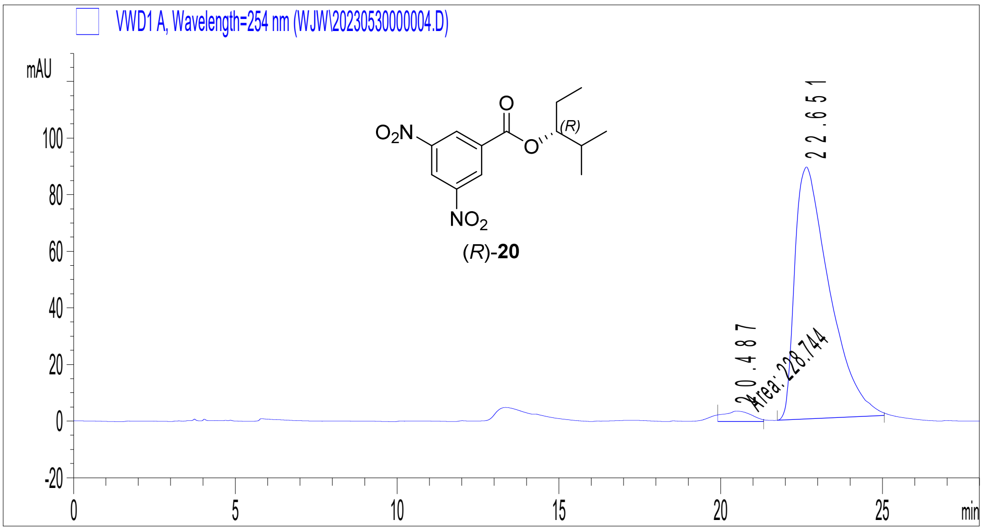

Peak #	RetTime [min]	Type	Width [min]	Area mAU*s	Height [mAU]	Area %
1	20.487	MM	1.0038	228.74382	3.79792	3.3283
2	22.651	BB	1.1169	6644.04541	89.01419	96.6717

7. References

1. Allu, S. R.; Banne, S.; Jiang, J.; Qi, N.; Guo, J.; He, Y. A unified synthetic approach to optically pure curvularin-type metabolites. *J. Org. Chem.* **2019**, *84*, 7227-7237.
2. Quinkert, G.; Fernholz, E.; Eckes, P.; Neumann, D.; Duerner, G. Synthesis of (+)-aspicillin using buildings blocks from renewable resources. *Helv. Chim. Acta* **1989**, *72*, 1753-1786.
3. Sargent, B. T.; Alexanian, E. J. Cobalt-catalyzed aminocarbonylation of alkyl tosylates: Stereospecific synthesis of amides. *Angew. Chem., Int. Ed.* **2019**, *58*, 9533-9536.
4. Mori, K.; Akasaka, K. Pheromone synthesis. Part 256: Synthesis of the four stereoisomers of 5,11-dimethylpentacosane, a new sex pheromone component of the male *Galleria mellonella* (L.), with high stereochemical purities as determined by the derivatization-HPLC analysis of the eight stereoisomers of 5,11-dimethyl-8-pentacosanol. *Tetrahedron* **2015**, *71*, 4102-4115.
5. Horner, J. H.; Newcomb, M. (ω-2,ω-2,ω-3,ω-3)-Tetradeuterio-fatty acids for mechanistic studies of enzyme-catalyzed hydroxylation reactions. *J. Labelled Compd. Radiopharm.* **2012**, *55*, 406-410.
6. Koppenhoefer, B.; Schurig, V. (*S*)-2-Chloroalkanoic acids of high enantiomeric purity from (*S*)-2-amino acids: (*S*)-2-chloropropanoic acid. *Org. Synth.* **1988**, *66*, 151-156.
7. Zhang, J.; Sheng, W.; Gholami, H.; Nehira, T.; Borhan, B. Di(1-naphthyl) methanol ester of carboxylic acids for absolute stereochemical determination. *Chirality* **2018**, *30*, 141-146.
8. Hattori, H.; Roesslein, J.; Caspers, P.; Zerbe, K.; Miyatake-Ondozabal, H.; Ritz, D.; Rueedi, G.; Gademann, K. Total synthesis and biological evaluation of the glycosylated macrocyclic antibiotic mangrolide A.

Angew. Chem., Int. Ed. **2018**, *57*, 11020-11024.

9. Koppenhoefer, B.; Schurig, V. (*R*)-Alkyloxiranes of high enantiomeric purity from (*S*)-2-chloroalkanoic acids via (*S*)-chloro-1-alkanols: (*R*)-methyloxirane. *Org. Synth.* **1988**, *66*, 160-172.
10. Sunagawa, S.; Yamada, H.; Naito, M.; Yasui, E.; Mizukami, M.; Miyashita, M.; Nagumo, S. Synthetic study of arenicolide C: Stereoselective synthesis of the C19-C36 segment. *Tetrahedron Lett.* **2015**, *56*, 6693-6695.
11. Taguri, T.; Yamamoto, M.; Fujii, T.; Muraki, Y.; Ando, T. Synthesis of four stereoisomers of (*S*)-2-methylpent-3-yl 3,13-dimethylpentadecanoate, a sex pheromone of the bagworm moth *Clania variegata*, using stereospecific inversion of secondary sulfonates as a key step. *Eur. J. Org. Chem.* **2013**, *2013*, 6924-6933.
12. Dale, J. A.; Dull, D. L.; Mosher, H. S. A-methoxy-α-trifluoromethylphenylacetic acid, a versatile reagent for the determination of enantiomeric composition of alcohols and amines. *J. Org. Chem.* **1969**, *34*, 2543-2549.
13. Hamada, T.; Daikai, K.; Irie, R.; Katsuki, T. Insect pheromone synthesis using Mn-salen catalyzed asymmetric epoxidation as a key step. *Tetrahedron: Asymmetry* **1995**, *6*, 2441-2451.
